# Feces Derived Allergens of *Tyrophagus putrescentiae* Reared on Dried Dog Food and Evidence of the Strong Nutritional Interaction between the Mite and *Bacillus cereus* Producing Protease Bacillolysins and Exo-chitinases

**DOI:** 10.3389/fphys.2016.00053

**Published:** 2016-02-24

**Authors:** Tomas Erban, Dagmar Rybanska, Karel Harant, Bronislava Hortova, Jan Hubert

**Affiliations:** ^1^Laboratory of Proteomics, Biologically Active Substances in Crop Protection, Crop Research InstitutePrague, Czech Republic; ^2^Department of Plant Protection, Faculty of Agrobiology, Food and Natural Resources, Czech University of Life Sciences PraguePrague, Czech Republic; ^3^Biology Section, Laboratory of Mass Spectrometry, Service Labs, Faculty of Science, Charles University in PraguePrague, Czech Republic

**Keywords:** *Tyrophagus putrescentiae*, allergen, *Bacillus cereus*, bacillolysin, protease, exochitinase, symbiosis, nutrition

## Abstract

*Tyrophagus putrescentiae* (Schrank, 1781) is an emerging source of allergens in stored products and homes. Feces proteases are the major allergens of astigmatid mites (Acari: Acaridida). In addition, the mites are carriers of microorganisms and microbial adjuvant compounds that stimulate innate signaling pathways. We sought to analyze the mite feces proteome, proteolytic activities, and mite-bacterial interaction in dry dog food (DDF). Proteomic methods comprising enzymatic and zymographic analysis of proteases and 2D-E-MS/MS were performed. The highest protease activity was assigned to trypsin-like proteases; lower activity was assigned to chymotrypsin-like proteases, and the cysteine protease cathepsin B-like had very low activity. The 2D-E-MS/MS proteomic analysis identified mite trypsin allergen Tyr p3, fatty acid-binding protein Tyr p13 and putative mite allergens ferritin (Grp 30) and (poly)ubiquitins. Tyr p3 was detected at different positions of the 2D-E. It indicates presence of zymogen at basic pI, and mature-enzyme form and enzyme fragment at acidic pI. Bacillolysins (neutral and alkaline proteases) of *Bacillus cereus* symbiont can contribute to the protease activity of the mite extract. The bacterial exo-chitinases likely contribute to degradation of mite exuviae, mite bodies or food boluses consisting of chitin, including the peritrophic membrane. Thus, the chitinases disrupt the feces and facilitate release of the allergens. *B. cereus* was isolated and identified based on amplification and sequencing of 16S rRNA and *motB* genes. *B. cereus* was added into high-fat, high-protein (DDF) and low-fat, low-protein (flour) diets to 1 and 5% (w/w), and the diets palatability was evaluated in 21-day population growth test. The supplementation of diet with *B. cereus* significantly suppressed population growth and the suppressive effect was higher in the high-fat, high-protein diet than in the low-fat, low-protein food. Thus, *B. cereus* has to coexist with the mite in balance to be beneficial for the mite. The mite-*B. cereus* symbiosis can be beneficial-suppressive at some level. The results increase the veterinary and medical importance of the allergens detected in feces. The *B. cereus* enzymes/toxins are important components of mite allergens. The strong symbiotic association of *T. putrescentiae* with *B. cereus* in DDF was indicated.

## Introduction

During digestion, the digestive enzymes enter the peritrophic space and are mixed to ingest food inside a food bolus (Terra, 2001; Sobotnik et al., 2008a). In insects, and probably in mites, a portion of the enzymes is recycled, but a significant portion remains present in the feces (Terra, 1990). Digestive enzymes make up a significant proportion of feces proteins (Ortego et al., 2000; Erban and Hubert, 2008, 2010b, 2015; Erban et al., 2009a). Mites are associated with microorganisms, including bacteria and fungi. The bacteria and fungi contribute to mite nutrition by producing exo-enzymes that predigest the substrate by serving directly as a food source (Sinha, 1966; Hubert et al., 2003, 2012a,b; Smrz, 2003; Erban and Hubert, 2008; Naegele et al., 2013). From a sanitary view, the mite feces are the most significant contaminants of the human environment because they contain major allergens that accumulate and persist in the environment (Tovey et al., 1981; de Boer et al., 1995; Sidenius et al., 2002; Platts-Mills and Woodfolk, 2011). The mites carry microorganisms that have adjuvant functions and stimulate innate signaling pathways leading to allergy (Jacquet, 2013). Specific microbiota are associated with and may modulate inflammatory processes in patients with severe asthma and related phenotypes (Huang et al., 2015).

*Tyrophagus putrescentiae* (Schrank, 1781) is a generalist feeder that appears in diverse habitats (Hughes, 1976; Smrz and Jungova, 1989; Athanassiou et al., 2002; Klimov and OConnor, 2015), but it prefers a source of high fat and protein, such as dry dog food (DDF), where it rapidly reproduces (Brazis et al., 2008; Erban et al., 2015b; Rybanska et al., 2016). It is considered the most common pest in DDF, so *T. putrescentiae* is of high veterinary importance. In dogs, it causes atopic dermatitis (Brazis et al., 2008; Gill et al., 2011). The mite is not only found in stored products but also in house dust. Therefore, *T. putrescentiae* is a domestic mite as well as a stored-product mite (SPM) (Colloff and Spieksma, 1992). The antigenic properties of *T. putrescentiae* to human and dogs have been studied, and allergenic cross-reactivity between *T. putrescentiae* and house-dust mites (HDMs) has been shown (Arlian et al., 1984; Park et al., 1999; Saridomichelakis et al., 2008; Marsella and Saridomichelakis, 2010). The physiological characteristics of mite gut pH (Erban and Hubert, 2010b) and digestive enzymes (Ortego et al., 2000; Erban and Hubert, 2008, 2010a; Erban et al., 2009a) indicate similar digestive and enzyme properties of *T. putrescentiae* as in other species of SPMs and HDMs.

The *in vitro* study of gut derived enzymes in acaridid mites is limited, due to the size of the mites, to analysis of spent growth medium extract (SGME) comprising the mites, remaining food and mite feces (Stewart et al., 1991) or feces extract (Erban and Hubert, 2015). Study of the feces proteome of *Dermatophagoides farinae* using a 2D-E-MS/MS proteomic approach detected and thereby increased the quantitative allergenic importance of six proteins/allergens, Der f1, Der f2, Der f3, Der f6, Der f15, and ferritin (Der f30) (Erban and Hubert, 2015). In addition, proteomic analysis enables the detection of proteins of microbial origin in a heterogeneous sample, providing additional information about the proteins of the investigated organism (Erban and Hubert, 2015; Erban et al., 2015a). The database of allergens currently (to date 23/01/2016 at www.allergen.org) registers some equally allergenic groups in HDMs and SPMs. In particular, in *T. putrescentiae*, there are currently five known allergens belonging to mite allergenic groups 2 (NPC2 family), 3 (Trypsin), 10 (Tropomyosin), 13 (Fatty-acid binding protein), and 34 (Troponin C). A proteomic analysis of *T. putrescentiae* feces might identify the gut-derived allergens/proteins and show their quantitative importance for allergy and/or contribution to digestive physiology of the mite.

In this study, the *T. putrescentiae* feces extract was analyzed via enzymatic and proteomic methods to identify the feces allergens with primary focus on proteolytic activities. The proteomic analysis identified bacillolysins, exo-chitinases and other proteins that originate from *Bacillus cereus*. The bacterial symbiont was isolated and identified using amplification and sequencing of the 16S rRNA and *motB* genes. This study demonstrates mite-bacterial symbiosis. The mite feces are not only a source of mite major allergenic proteins; they also contain bacterial compounds that may significantly contribute to allergy.

## Materials and methods

### Experimental mite

A laboratory strain of *T. putrescentiae* collected in 1996 by Eva Zdarkova in Bustehrad, Czechia, was used in the study. The mite has been kept and reared in laboratory colonies in the Crop Research Institute in Prague since collection (Erban and Hubert, 2008). Prior to the experiment, the mites were removed from the standard rearing diet and fed DDF that was crushed by using a mortar and pestle (brown kernels, Friskies Life Plus Nutrition Junior, Nestle Purina, Buk, Hungary) for nutritional adaptation (Erban et al., 2015b). The mites were cultivated on an IWAKI 25-cm^2^ surface area in 70-mL-capacity tissue culture flasks (IWAKI flasks; Cat No. 3100-025; Sterilin, Newport, UK). Constant 85% relative humidity (RH) was maintained with saturated KCl solution in Secador desiccator cabinets (Bel-Art Products, Pequannock, NJ, USA), and the air-conditioned dark room was maintained at 25 ± 1°C.

The mites previously reared for a long period on the DDF were collected. Then, the mites were placed into culture flasks with 50 mg of the DDF diet and reared for 10 weeks for feces production. The mite culture was controlled periodically twice weekly, and 20–40 mg feed was added as needed. The flasks were turned 180° at each inspection to facilitate fecal coverage of all flask surfaces (Erban and Hubert, 2015). The flask contents were processed for protein extraction.

### Preparation of feces, spent growth medium, and control extracts for protein analysis

Spent growth medium (SGM) was removed from the flasks and placed in 50 mL centrifugal tubes (Orange Scientific, Braine-l'Alleud, Belgium) for SGME preparation. Briefly, 0.1 g of SGM was used per 1 mL of HPLC grade water (ddH_2_O). The feces-coated tissue culture flasks after SGM and mite removal were filled with 5 mL of ice-cold (4°C) ddH_2_O. The flasks were placed on ice and mixed on a ProBlot Rocker (Labnet International, Woodbridge, NJ, USA) for 15 min per side at 100 rpm. The feces extract was removed to 50 mL centrifugal tubes and stored on ice. The extraction step was repeated twice with 10 and 5 mL of the ddH_2_O per step. For SGME preparation, the centrifugal tube with content was mixed on ProBlot Rocker for 50 min on ice. The extracts were centrifuged using a MR 23i centrifuge (Jouan Industries, France) at 10,000 g and 4°C for 15 min. After centrifugation, the supernatants were removed with a 10-mL pipette. For control samples, the mite feed was extracted analogously to the feces; specifically, 0.1 g of DDF was used per 1 mL of ddH_2_O. SGME was directly used for enzymatic analysis. Feces extract was divided into aliquots and added to 50-mL centrifuge tubes, which were covered with a 0.22-μm PTFE filter (Cat No. TR-200210, Teknokroma, Spain) and fixed with a vented cap. The tubes were frozen and lyophilized in PowerDry LL3000 (Thermo, Shanghai, China) and stored at −40°C for later use. The protein content was determined by Bradford assay (B6916, Sigma-Aldrich, St. Louis, MO, USA) with BSA as the standard.

### Profiling of optimal proteolytic pH

Azoalbumin (Cat. No.: A2382; Sigma-Aldrich) was used as a substrate for screening general protease activity. The pH optimum was screened in the pH range from 3.5 to 8.0. The assay was performed on a microplate organized into four replicates of each enzymatic assay and two replicates of each control sample at different pH values. Universal Britton–Robinson buffer I (BR-I) at a concentration of 0.125 M and supplemented with 0.2% azocalbumin was applied to the 96-well microplates (Gama, Ceske Budejovice, Czechia). Reactions were started by adding 50 μL of SGME containing 20 μg total proteins. The microplates were covered with adhesive seals (Simport, Beloeil, QC, Canada) and incubated for 1 h at 37°C in a thermoshaker (DTS-2) at 700 rpm. After removal of the seals, reactions were stopped by adding 50 μL of 0.3 M trichloroacetic acid (TCA). The microplates were centrifuged at 3500 rpm for 20 min at 4°C in an MR 23i centrifuge. Seventy microliters of each supernatant was transferred into a 384-well microplate, and the absorbance was read at 405 nm (Multiskan Ascent, Thermo, USA). Two different controls were analyzed, one for mite homogenate and one for the enzyme substrate.

### Specific protease activity levels at three pH values

The specific protease activity was measured at pH 5.75, 6.75, and 8.00. The following 4-nitroaniline-bound specific substrates were used: N_α_-Benzoyl-DL-arginine 4-nitroanilide hydrochloride (BapNA, Cat. No. B4875, Sigma-Aldrich), Ala-Ala-Phe p-nitroanilide (AAPpNA, Cat. No. A9148, Sigma-Aldrich) and Z-Arg-Arg p-nitroanilide (ZRRpNA, Cat. No. P-138, Biomol, Exeter, UK) were used to screen (i) trypsin, (ii) chymotrypsin, and (ii) cysteine protease activity, respectively. Trypsin inhibitor N_α_-Tosyl-L-lysine chloromethyl ketone hydrochloride (TLCK; Cat. No. T7254, Sigma-Aldrich) dissolved in HPLC grade methanol, chymotrypsin inhibitor N-p-Tosyl-L-phenylalanine chloromethyl ketone (TPCK; Cat. No. T4376, Sigma-Aldrich) dissolved in Dimethyl sulfoxide (DMSO; Cat. No. D8414, Sigma-Aldrich), and cysteine protease inhibitor *trans*-Epoxysuccinyl-L-leucylamido(4-guanidino)butane (E-64, Cat. No. E3132, Sigma-Aldrich) dissolved in ddH_2_O, and reagent for the selective reduction of disulfides tris(2-carboxyethyl)phosphine hydrochloride (TCEP; Cat. No. C4706, Sigma-Aldrich) dissolved in ddH_2_O were used to determine the specificity of the proteolytic activity. Ten microliters of TLCK, TPCK, E64, or TCEP was applied to the appropriate wells of the microplate in four replicates; control wells contained only 10 μL of ddH_2_O or DMSO with inhibitors. Then, 190 μL of substrate solution BapNA, AAPpNA or ZRRpNA in 0.132 M BR-I was added to each well. The reaction was started by adding 50 μL of mite homogenate containing 20 μg total proteins. The microplates were covered with adhesive seals and incubated for 15 min in the case of ZRRpNA and BApNA and for 35 min in the case of AAPpNA at 37°C in a DTS-2 thermoshaker at 700 rpm. After removal of the seals, reactions were stopped by adding 50 μL of 0.3 M TCA. The microplates were centrifuged at 3500 rpm for 20 min at 4°C in an MR 23i centrifuge. Seventy microliters of each supernatant was transferred into a 384-well microplate, and the absorbance was read at 405 nm. Two different controls were analyzed, one for mite homogenate and one for the enzyme substrate. Enzyme activity was expressed as the percentage of inhibition; the control was considered to have 100% activity. The effects of inhibitors or TCEP on enzyme activity at each pH and substrate concentration were evaluated using *t*-tests. An ANOVA was used to compare activities on the three substrates within the three tested pHs. The statistical analysis was performed with Statistica 12 (Statsoft, USA).

### Protease analysis by zymography in feces extract

The lyophilized and BSA-quantified feces extract was dissolved in sample buffer lacking reducing agent (2% SDS, 0.0625 M Tris-HCl, 10% Glycerol, pH 6.8). Protein (10, 20 or 30 μg) was separated on a 4–12% SDS-PAGE gel with the Mini Protean Tetra Cell (Bio-Rad, Hercules, CA, USA) with a 1.5 mm spacer width. Electrophoresis was run at a constant voltage of 25 V for 1 h, after which the proteins were separated at a constant voltage of 77 V in a cooled apparatus. The gel was rinsed with ddH_2_O and placed in phosphate buffered saline (PBS; pH 7.4). Then, the gel was placed on a glass transilluminator with a documentation system (InGenius, Syngene, Cambridge, UK) and was overlaid with fluorescence substrate solution dissolved in PBS. For zymography detection of protease activities, the following fluorogenic substrates (analogous to chromogenic substrates used in microplate based enzyme analysis) were used: trypsin substrate *N*_α_-benzoyl-L-arginine-7-amido-4-methylcoumarin hydrochloride (BAAMC; Cat. No. B7260, Sigma-Aldrich), chymotrypsin substrate Ala-Ala-Phe-7-amido-4-methylcoumarin (AAAMC; Cat. No. A3401, Sigma-Aldrich), and cathepsin B Z-Arg-Arg-7-amido-4-methylcoumarin hydrochloride (ZRRAMC; Cat. No. C5429, Sigma-Aldrich). The results were visualized with the InGenius documentation system with 360 nm transillumination and a 440 nm filter.

### 2D-E-MS/MS analysis of feces extract and the control dry dog food extract

The feces and control extracts were cleaned using a 2D Clean-Up Kit (Cat. No. 80-6484-51, GE Healthcare Lifesciences, Uppsala, Sweden). Isoelectric focusing (IEF) was performed on an Ettan IPG Phor 3 instrument (GE). The separation was performed in 13-cm ceramic strip holders, and Immobiline dry strips with pH ranges 3–10 (Cat. No. 17-6001-14, GE). A DeStreak Rehydration Solution (Cat No. 18-1168-31, GE) containing 0.5% pH 3–10 IPG buffer (Cat No. 17-6000-87, GE) were used for active rehydration. The separation program was as follows: (1) Step, 30 V, 10H (active rehydration); (2) Step, 500 V, 500 Vh; (3) Grad, 1000 V, 800 Vh; (4) Grad, 6000 V, 15,000 Vh and (5) Step 6000 V, 16,000 Vh. The isoelectric focusing program with active rehydration ran for 19 h and a total of 32,600 Vh. Immediately following IEF, the strips were equilibrated for 15 min in an equilibration buffer containing dithiothreitol (Cat No. 43817, Sigma-Aldrich), followed by 15 min in a buffer containing iodoacetamide (Cat No. 57670, Sigma-Aldrich). The strips were placed over an SDS-PAGE gel and fixed with 1% agarose (Cat No. A7431, Sigma-Aldrich). Electrophoresis was run at a constant voltage of 30 V for 50 min, after which the proteins were separated at a constant voltage of 300 V in a cooled apparatus. The gel was stained with 0.02% PhastGel Blue R (Cat. No. 17-0518-01, GE).

For MALDI TOF/TOF protein identification, spots with 0.5 mm to 1 mm inner diameters were selected from the Coomassie stained gels. The protein identification followed a described methodology (Erban and Hubert, 2015). The spectra were then subjected to searches against the non-redundant NCBI database (35,149,712 sequences; 12,374, 887,350 residues) using Mascot 2.2 (Matrix Science, Boston, MA, USA). The database search criteria were as follows: enzyme–trypsin; taxonomy–NCBInr; fixed modification–carbamidomethylation (C); variable modifications–deamidated (NQ), methionine oxidation (M); protein mass: unrestricted; peptide mass tolerance: ±125 ppm; fragment mass tolerance: ±0.3 Da and one missed cleavage allowed. The protein scores were derived from the ion scores as a non-probabilistic basis for ranking the protein hits.

### Isolation of bacteria strains

For bacterial isolation, six culture flasks of *T. putrescentiae* were selected. Before the extraction, mites were removed from the culture flasks without including the surface-adhered material. The feces-coated tissue culture flasks were filled with 3 mL sterile ddH_2_O and shaken. The extract was removed and placed in sterile 15 mL centrifugal tubes. Bacteria were isolated by serial dilutions of feces solutions and plated on nutrient agar (Difco, Franklin Lakes, NJ, USA). After 24 h of incubation at 25°C, single colonies of bacteria were selected and identified.

### DNA extraction, PCR amplification, and sequence determination of the bacterial strains

DNA was extracted from single colonies of bacteria using an Ultra Clean Microbial DNA Isolation Kit (Cat. No. 12224, MoBio Laboratories, Carlsbad, CA, USA) according to the manufacturer's instructions. For 16S rRNA analysis, the following primers were used: 27F (5′-AGAGTTTGATCMTGGCTCAG-3′) and 1492R (5′-GGTTACCTTGTTACGACTT-3′; Jiang et al., 2006). For identification within the *B. cereus* group, the following primers that target the *motB* gene were used: BCFomp1 (5′-ATCGCCTCGTTGGATGACGA-3′) and BCRomp1 (5′-CTGCATATCCTACCGCAGCTA-3′) (Oliwa-Stasiak et al., 2010). PCR amplification was performed using a C1000 thermal cycler (Bio-Rad, Hercules, CA, USA), and PCR mixtures in a total volume of 25 μL contained the following components: 2 mM MgCl_2_, 200 μM dNTPs, forward and reverse primers (100 nM each), 0.5U Taq polymerase (all Promega, Madison, WI, USA) and 1 μl DNA. For amplification of 16S rRNA the following conditions were used: 2 min at 94°C, and 30 cycles of 90 s at 94°C, 90 s at 50°C, and 60 s at 72°C, followed by final extension for 10 min at 72 and 4°C hold (Barbieri et al., 2001). PCR conditions for the gene *motB* were as follows: 30 cycles of 30 s at 94°C, 60 s at 54.5°C, and 60 s at 72°C, with an initial denaturation step for 5 min and a final extension step of 7 min (Oliwa-Stasiak et al., 2010). PCR products were purified and sequenced at Macrogen (Seoul, South Korea). The sequences were assembled and manually edited with CodonCode Aligner version 2.0.6. (CodonCode, Dedham, MA, USA). DNA sequences of 16S rRNA were obtained by using the Basic Local Alignment Search Tool (BLAST) (http://blast.ncbi.nlm.nih.gov/; Altschul et al., 1990). Nucleotide sequences of the *motB* gene were translated to protein sequences using the ExPASy Translate tool (Gasteiger et al., 2003) and further searched with BLASTP. All of the obtained sequences were deposited in the GenBank database.

### The effect of addition of *Bacillus cereus* on the population growth of *Tyrophagus putrescentiae*

This experiment tested whether the population growth of *T. putrescentiae* is influenced by different enrichment of two nutritionally different diets with lyophilized cells of *B. cereus*. The diet prepared of the DDF kernels provided a high-fat and high-protein diet, whereas whole-meal spelt flour (WSF; Bioharmonie, Czechia) provided a low-protein and low-fat diet. For preparation of *B. cereus* enriched diet, the isolate was cultivated on Petri dishes (90 mm) with nutrient agar (Difco). After 24 h of incubation at 25°C, were the bacterial colonies collected and transferred to 15 mL centrifugal tubes with 5 mL sterile ddH_2_O. The liquid was discarded after centrifugation at 788 g and 4°C for 5 min. Then were the bacteria washed two times with physiological saline solution and once with sterile ddH_2_O and centrifuged at 788 g and 4°C for 5 min. The supernatant was removed and bacteria were lyophilized. The lyophilized bacteria were weighted and dissolved in ddH_2_O and incorporated into the mite food; the calculated volume was 3 mL of solution per 1 g of food. Control diet was prepared analogously to the experimental diet, but the solution consisted only of ddH_2_O. The experimental diets were derived from DDF (see section experimental mite) or WSF and the diet was enriched by 1, 5, or 0% (control treatment) (w/w) with the lyophilized cells of *B. cereus*. The resulting combination was properly mixed using a MS1 Minishaker (IKA, Staufen, Germany) and lyophilized. The lyophilized material was ground into a powder in a pottery grinding mortar.

Prior to experiment, 6 mg (lyophilized weight) of WSF-type diet or 9 mg (lyophilized weight) of the DDF-type diet was placed to into 70-mL IWAKI flasks. The material was rehydrated 24 h before the experiment in a desiccator containing distilled water. The experiment was begun by placing 10 mite individuals of good condition and similar size from each of the treatments into the flasks. The experimental design included in total 12 replicates per treatment. The flasks were incubated for 21 days in the Secador desiccator cabinets (Bel-Art Products) under constant environmental conditions of 85% RH and 25°C. The experiment was terminated by the addition of 10 mL of 80% ethanol to the flasks. The mites were then counted using a dissection microscope (Carl Zeiss, Jena, Germany).

We applied Fisher's F-test for comparison of variance in final population density (N) on control treatments (DDF and WSF) without *B. cereus* additive. The data did not meet normal distribution; the nonparametric tests were used. The N on control treatment on both types of diet was compared by nonparametric Mann-Whitney test. Next, the effect of *B. cereus* additive diet on N was compared by Kruskal-Wallis test, separately. The data were analyzed using XLSTAT 2015 (Addinsoft, New York, NY, USA).

## Results

### Enzymatic analysis of SGME in microplate assays

**Figure 1:**
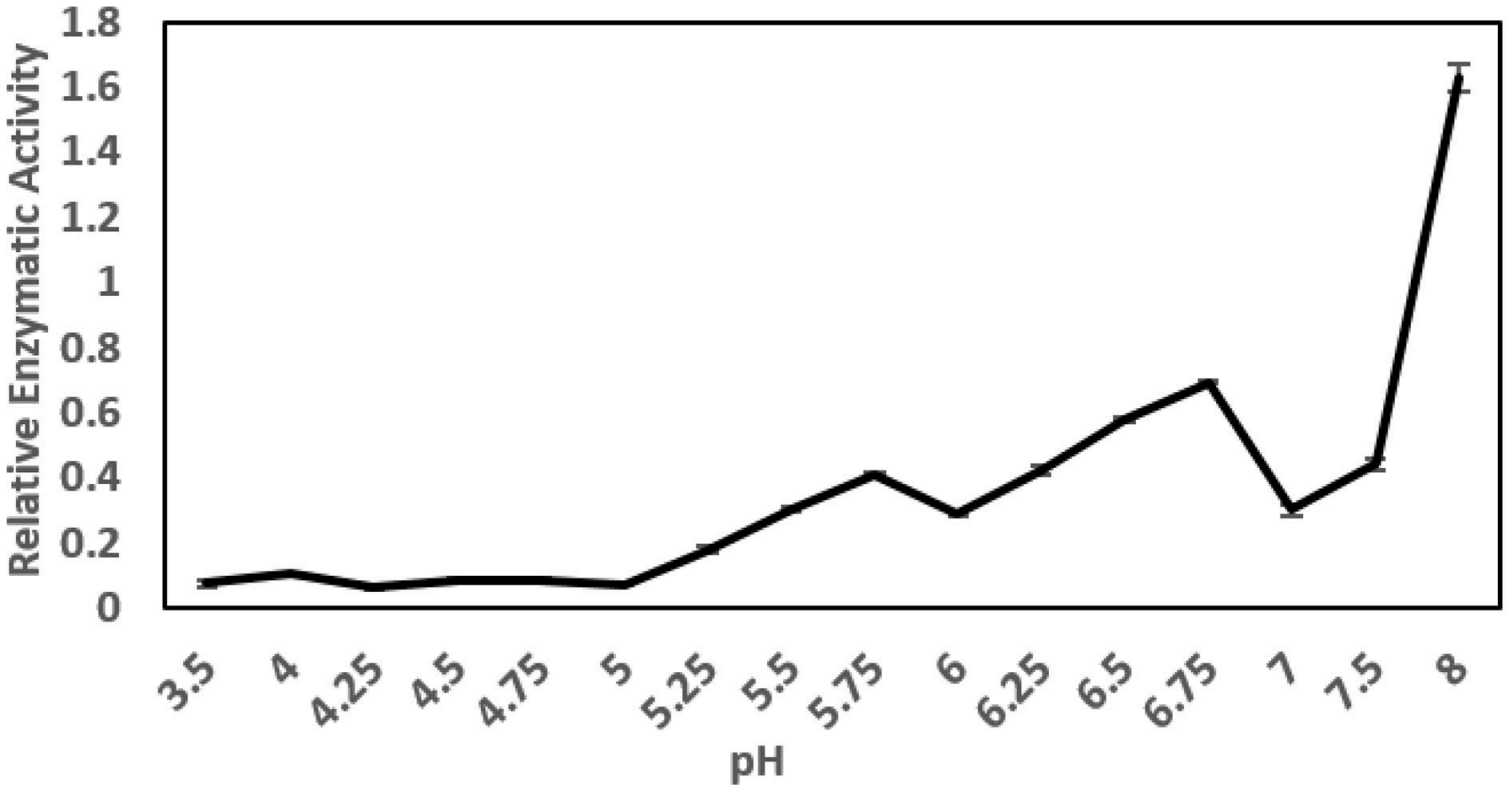
**General protease activity screening**. Three peaks of protease activity at pH 5.75 and 6.75 and at basic pH were observed. The relative enzymatic activity is expressed as ΔA/h.

**Figure 2:**
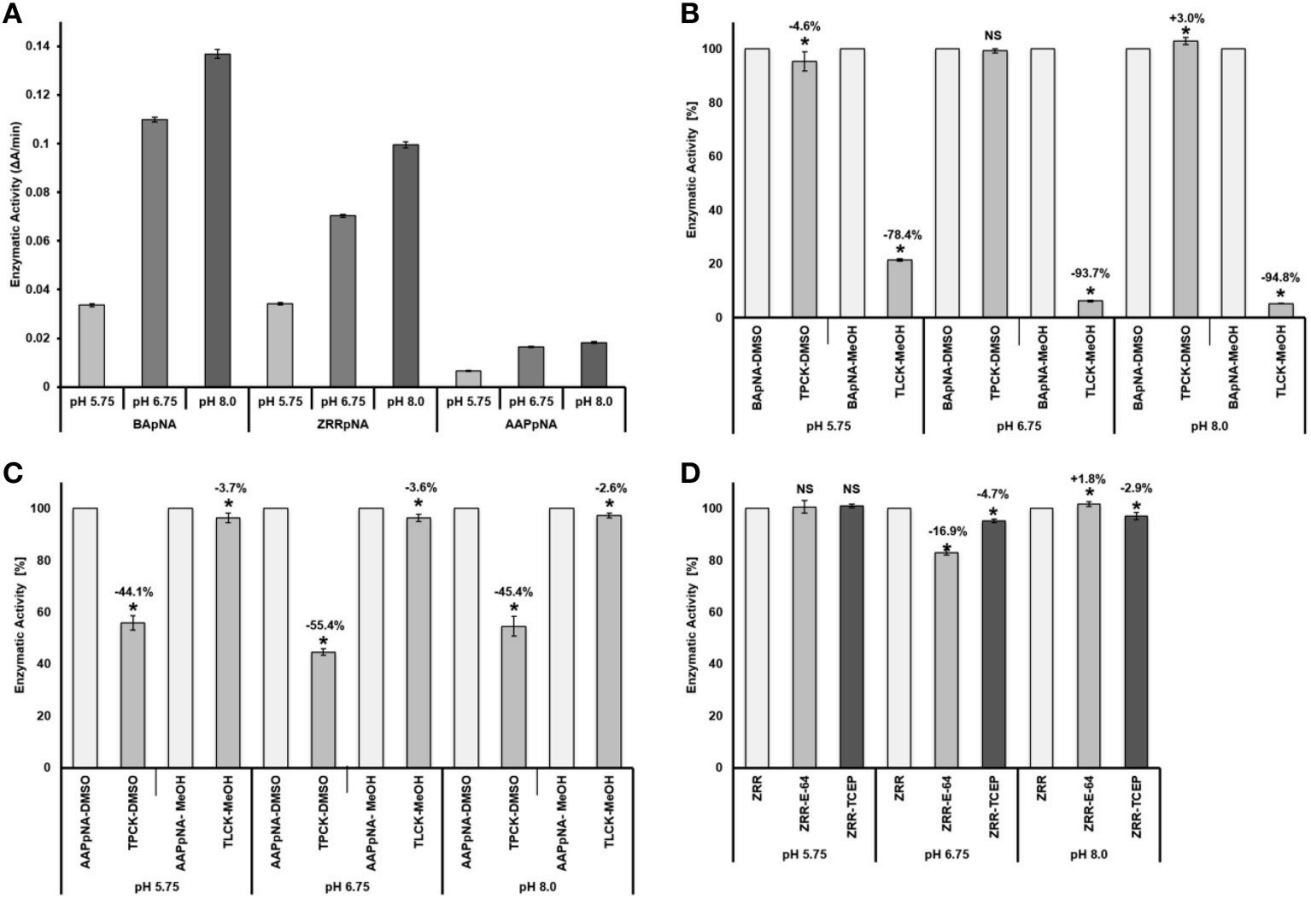
**Comparison and specify of the protease activities toward BApNA, ZRRpNA, and AApNA at pH 5.75, 6.75, and 8.0. (A)** Protease activities toward BApNA, ZRRpNA, and AApNA at pHs 5.75, 6.75, and 8.0; **(B)** Specifity of proteolytic activity on trypsin substrate BApNA; **(C)** Specifity of proteolytic activity on chymotrypsin substrate AAPpNA; **(D)** Specifity of proteolytic activity on cathepsin B substrate ZRRpNA.

The pH optimum screening of general protease activity between the pH range 3.5 to 8.0 showed three peaks of protease activity at pH 5.75, 6.75, and 8.00 (Figure 1). Both the general protease activity (Figure 1) and activity toward the three specific protease substrates BApNA, ZRRpNA, and AApNA (Figure 2A) had similar trend at all three pH values tested and increased in the following order: 5.75, 6.75, and 8.00. Specific protease activities were highest toward BApNA, followed by ZRRpNA and AApNA (Figure 2A). The one-way ANOVA model showed that enzymatic activities on the three substrates at all the three pHs tested were significantly different at the 0.05 level. The parameters of ANOVA for each substrate were: (i) BApNA: *F*_(2, 9)_ = 7243; *P* < 0.0001; (ii) ZRRpNA: *F*_(2, 9)_ = 6532; *P* < 0.0001; (iii) AAPpna: *F*_(2, 9)_ = 1838; *P* < 0.001.

A specific trypsin inhibitor, TLCK, strongly inhibited (in a range from 78 to 95%) the proteolytic activity toward the trypsin substrate BApNA at all three pHs (Figure 2B). The inhibition effect of TLCK on BApNA was higher at pH 6.75 and 8.00 compared to 5.75. The chymotrypsin inhibitor TPCK was also found to slightly influence the proteolytic activity toward BApNA. At pH 5.75, the activity decreased, but at pH 8.0, the enzyme activity increased.

The specific chymotrypsin inhibitor TPCK decreased the proteolytic activity (in the range of 44–55%) toward the chymotrypsin substrate AAPpNA at all three pHs (Figure 2C). The inhibition effect of TLCK on AAPpNA was higher at pH 6.75 compared to pH 5.75 and 8.00. TLCK was also found to mildly inhibit the proteolytic activity toward AApNA at all three pH levels.

Both the specific cathepsin B inhibitor E-64 and the reducing agent TCEP mildly influenced the proteolytic activity toward cathepsin B substrate ZRRpNA (Figure 2D). Both E-64 and TCEP had no significant effect on enzyme activity at pH 5.75. TCEP slightly decreased the proteolytic activity toward ZRRpNA at pH 6.75 and 8.00. The inhibitory effect of E-64 on proteolysis of ZRRpNA was found to be 16.9% at pH 6.75, whereas at pH 8.0 it slightly increased the proteolytic activity.

### Protease analysis of feces extract by zymography

**Figure 3:**
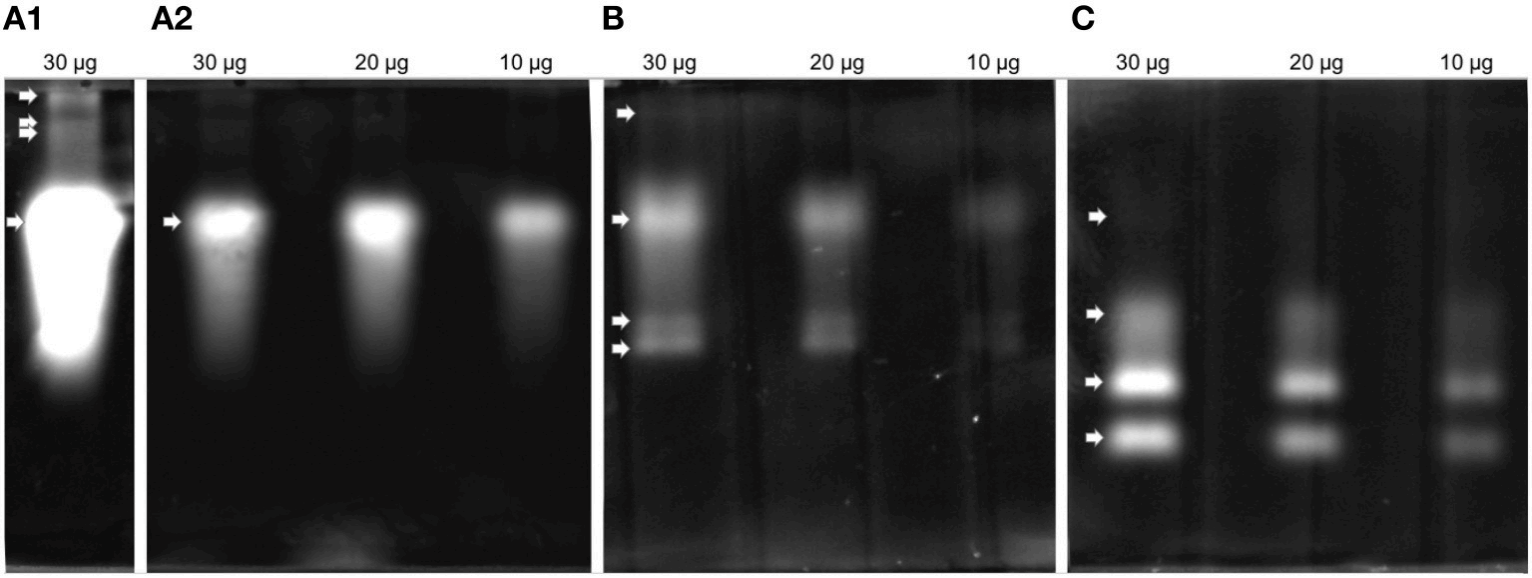
**Protease analysis by zymography in feces extract. (A)** BAAMC–trypsin substrate, **(A1)**, long time exposition; **(A2)**, accurate capture time; **(B)** ZRRAMC–cathepsin B substrate; **(C)** AAAMC–chymotrypsin substrate.

Proteolytic activity was detected in all four gel enzyme assays. Zymogram patterns differed from each other (Figure 3). The trypsin substrate BAAMC produced one strong band (Figure 3A2) and three weak bands of higher molecular weight (Figure 3A1). The cathepsin B substrate ZRRAMC (Figure 3B) produced four bands, two of similar size to BAAMC. The chymotrypsin substrate AAAMC (Figure 3C) produced four bands, two strong bands of lower MW than BAAMC and ZRRAMC and one band which had the weakest signal and was of similar size to the strong signal of BAAMC and ZRRAMC.

### 2D-E-MS/MS analysis of feces extract

**Figure 4:**
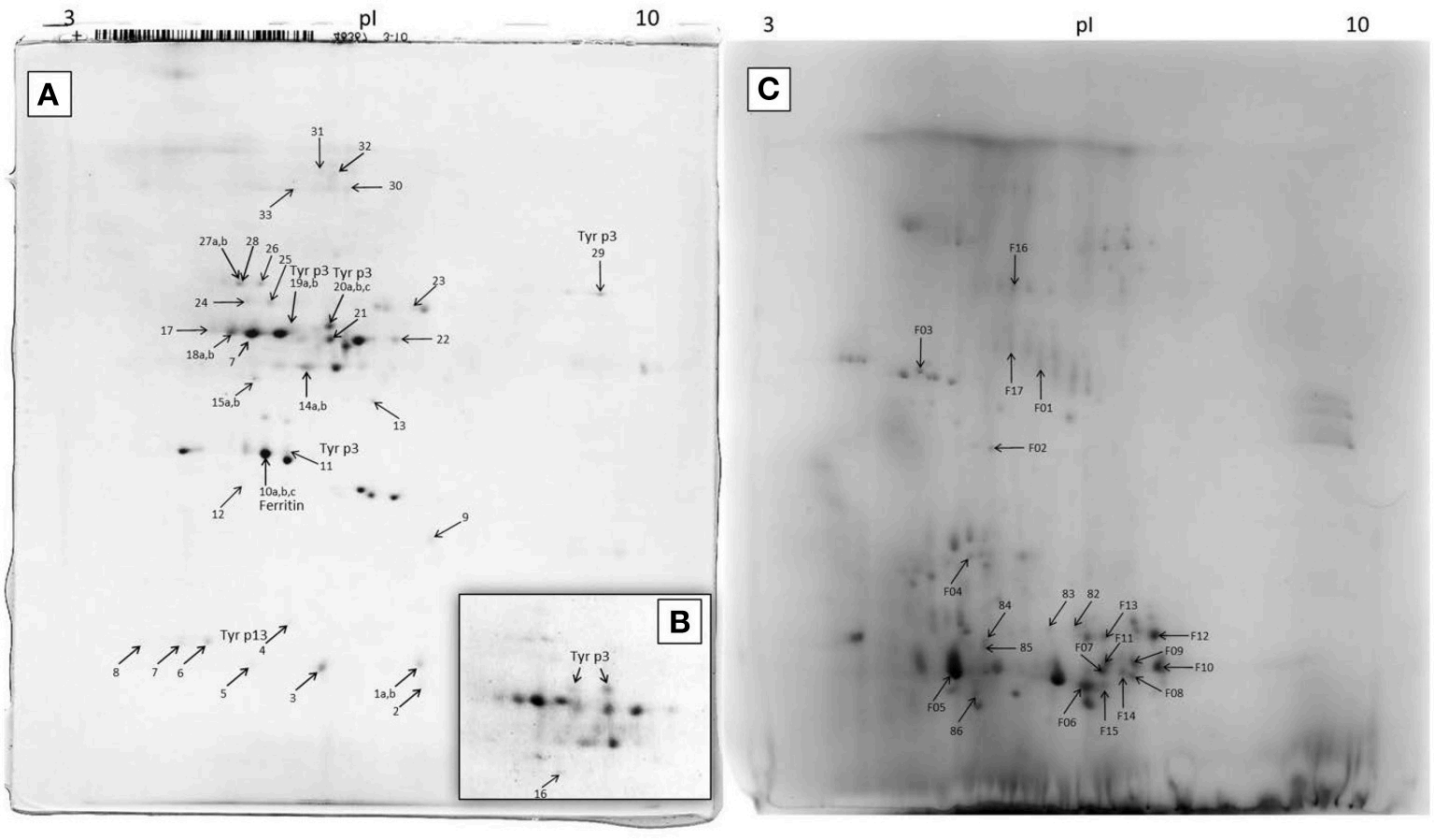
**2D-E image of ***Tyrophagus putrescentiae*** feces extract and control dry dog food extract with MS/MS identified proteins. (A)** entire representative 2D-E; **(B)** detail of spots corresponding to Tyr p3 from another sample; **(C)** 2D-E image of control dry dog food protein extract of mite “food” proteins. Spots No. 19 and 20 are mature trypsin Tyr p3, spot No. 29 at the basic pI is apparently proenzyme of Tyr p3, and spot No. 11 is fragment of Tyr p3. The qualitatively different 2D-E patterns of images **(A,B)** show protein disappearance from the control DDF extract and demonstrate the powerful mite-bacterial digestive proteolytic activity in our experiment. See Table 1 for the list of MS/MS identified proteins.

The 2D-E of the feces extract (Figure 4A) qualitatively differed from the 2D-E of the control extract of untreated DDF (Figures 4A,C). Comparison of the two 2D-E patterns and MS/MS results revealed almost complete depletion of proteins of the DDF after passing through the mite gut. An exception was spot 9 in the 2D-E of feces extract that was the *Triticum aestivum* α-amylase inhibitor (α-AI), and it was remainder of the food source.

**Figure 5:**
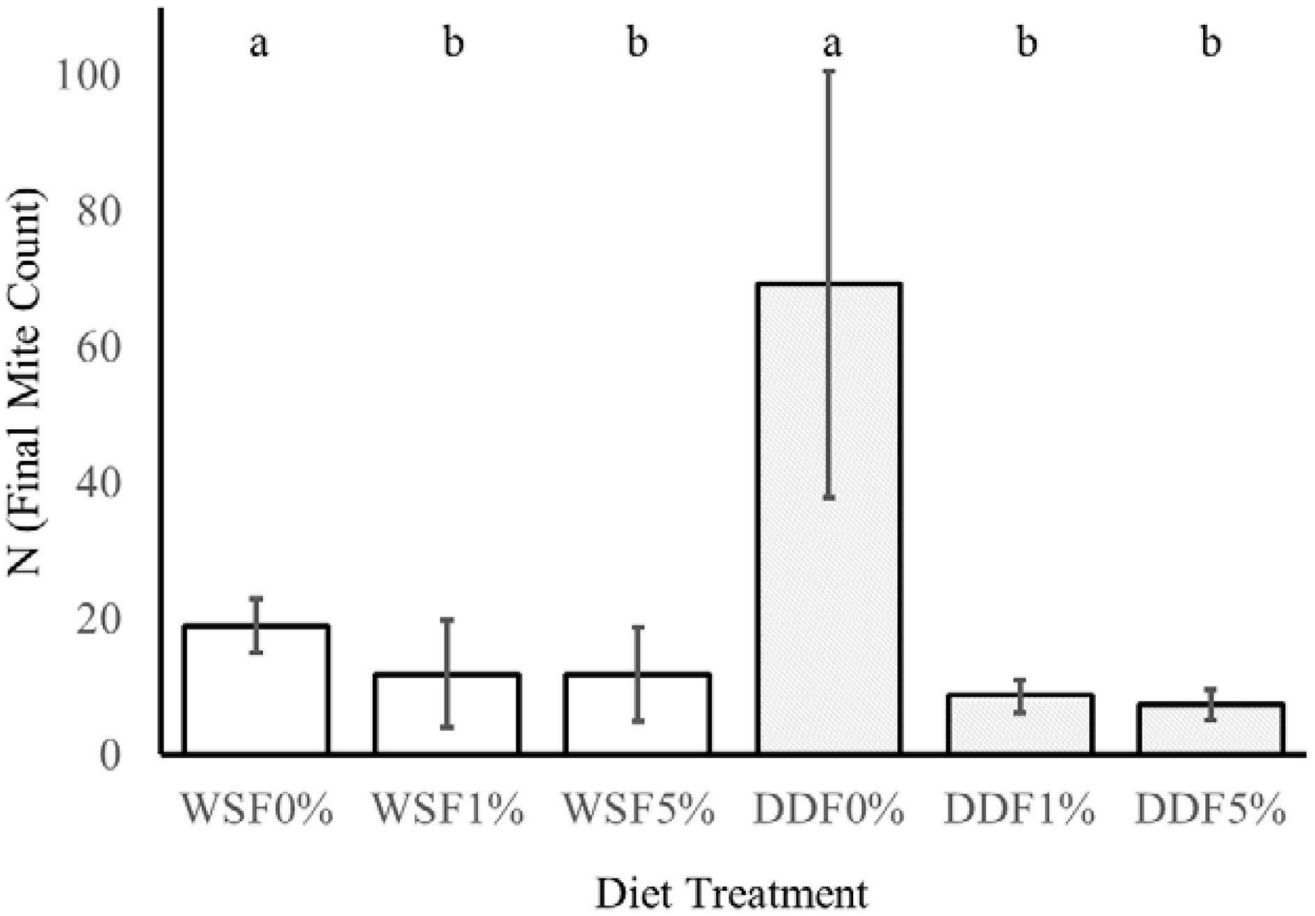
**The effect of ***Bacillus cereus*** addition to the diet on the growth of mites**. This experiment tested the influence of different additive (0, 1, 5% w/w) of lyophilized *B. cereus* isolate to diet on two nutritionally different diets whole-meal spelt flour (WSF) and DDF. The lower case letters a and b indicate statistical difference (Kruskal-Wallis) between the control and bacterial treatments within diet.

In the 2D-E of the feces extract, there were proteins exactly assigned to mite and bacterium (Figure 4; Table 1). The fatty acid-binding protein, a Tyr p13 allergen, and trypsin protease, a Tyr p3 allergen, were assigned to the mite *T. putrescentiae* according to the current NCBI records. The proteins suggested for the mite by metazoan similarity were ubiquitin/polyubiquitin spots and ferritin. Some other protein spots (e.g., 17 and 18) were also significant by Mascot; however, whether these proteins are produced by the mite is unclear though result indicated sequence similarity to protein of another invertebrate *Caenorhabditis*. In the 2D-E of the control sample of DDF, MS/MS identified 22 spots (Figure 5; Table 1); most of these results were assigned to the *Triticum aestivum* α-amylase inhibitor (α-AI).

**Table 1:** **Proteins identified in ***T. putrescentiae*** feces extract by 2D-E-MS/MS**.

**Spot No**.	**Protein name**	**Origin**
1ab, 2, 3, 5	Ubiquitin	“Suggest TP”
4	Tyr p13–fatty acid-biding protein	*T. putrescentiae*
19ab, 20abc, 29, 11	Tyr p3–trypsin	*T. putrescentiae*
10abc	Ferritin	“Suggest TP”
6, 7, 8	Cold-shock protein	*Bacillus cereus*
12	Nucleoside diphosphate kinase	*Bacillus cereus*
13, 15ab	superoxide dismutase	*Bacillus cereus*
22, 23	Bacillolysin–alkaline serine protease	*Bacillus cereus*
24, 25	Bacillolysin–neutral protease	*Bacillus cereus*
26, 27ab	Bacillolysin–neutral protease	*Bacillus cereus*
28	Extracellular exo-chitinase	*Bacillus cereus*
30, 31	Peptide ABC transporter	*Bacillus cereus*
32, 33	1-pyrroline-5-carboxylate dehydrogenase	*Bacillus cereus*
9	α-AI inhibitor [*T. aestivum*]	Dry dog food
14a	Triosephosphate isomerase [*Xiphophorus*]	?
14b	Ribonuclease [*Geobacillus*]	?
16	Superoxide dismutase [*Candida*]	?
17, 18a	Hypothetical protein CRE_08947 [*Caenorhabditis*]	?
18b	Hypothetical protein RB5404 [*Rhodopirellula*]	?
21	hbt1p [*Saccharomyces*]	?
F01	Alpha S1 casein, partial [*Bos taurus*]	Dry dog food
F02	Hypothetical protein F775_08244 [*Aegilops tauschii*]	Dry dog food
F03	Hypothetical protein F775_14150 [*Aegilops tauschii*]	Dry dog food
F04	Globulin 3 [*Triticum aestivum*]	Dry dog food
F05	Dimeric alpha-amylase inhibitor [*Triticum dicoccoides*]	Dry dog food
F06	Monomeric alpha-amylase inhibitor [*Triticum monococcum*]	Dry dog food
F07	Alpha-amylase inhibitor 0.19 [*Triticum aestivum*]	Dry dog food
F08	0.19 dimeric alpha-amylase inhibitor [*Triticum aestivum*]	Dry dog food
F09	0.19 dimeric alpha-amylase inhibitor [*Triticum aestivum*]	Dry dog food
F10	Alpha-amylase inhibitor 0.19 [*Triticum aestivum*]	Dry dog food
F11	Alpha-amylase inhibitor 0.19 [*Triticum aestivum*]	Dry dog food
F12	Alpha-amylase inhibitor 0.19 [*Triticum aestivum*]	Dry dog food
F13	Alpha-amylase inhibitor 0.19 [*Triticum aestivum*]	Dry dog food
F14	RecName: Full = Wheatwin-1	Dry dog food
F15	Monomeric alpha-amylase inhibitor [*Triticum aestivum*]	Dry dog food
F16	Regulator [*Bacillus macauensis*]	Dry dog food
F17	Argininosuccinate synthase [*Sinorhizobium medicae*]	Dry dog food

*All the proteins listed were MS/MS identified. The T. putrescentiae mite allergens and enzymes of Bacillus cereus bacterial symbiont are of key interest regarding medical importance. The spots 1-33 denote results of the mite feces extract (see Figures 4A,B), while spots F01–F17 denote control dry dog food protein extract of mite “food” identified proteins (see Figure 4C). Lower case letters at Spot. No. indicate independent replicates of MS/MS identifications from different 2D-E gels. For the details of protein identifications see the Tables S1, S2*.

### Isolation and identification of the bacteria strain

Six bacterial isolates (denoted as TPFDDF-15-001—TPFDDF-15-006) were obtained from the six flasks coated with feces of *T. putrescentiae* cultured on DDF. The plated colonies were morphologically uniform: irregular, flat with a lobate margin and cream color. The 16S rRNA gene sequence analysis (GenBank Accession Numbers: KT619128, KT619129, KT619130, KT619131, KT619132, KT619133) of the obtained sequences showed 99% identity to *B. cereus*. The sequences of the *motB* gene (GenBank Accession Numbers: KT868888, KT868889, KT868890, KT868891, KT868892, KT868893) from the six isolates showed 100% identity with the *B. cereus* chemotaxis protein MotB (WP016077917.1). No sequence variation in sequences of 16S rRNA and *motB* genes was observed between the six isolates. Therefore, we considered the six isolates to be a single strain.

### The effect of *Bacillus cereus* addition to the diet on the growth of mites

On control diets without any addition of *B. cereus*, the final numbers of mites *T. putrescentiae* (N) significantly differed in their variances [*F*_(1, 22)_ = 61, *P* = < 0.0001; Figure 5]. The higher variance was in DDF-type diet than on WSF-type diet. The significant differences were found in the final population on control treatments (DDF and WSF) [*U*_(1, 22)_ = 0, *P* = < 0.0001]. The final population density was 3.6 times higher on DDF-type control diet than on the WSF-type control diet.

The addition of *B. cereus* significantly reduced the final population density of mites on both diets tested, i.e., [*K*_(2, 32)_ = 10, *P* = 0.007] for WSF-type diet and DDF-type diet [*K*_(2, 32)_ = 24, *P* = 0.0001]. In the case of DDF treatment, the population growth decreased 8 and 9 times after 1 and 5% diet enrichment of *B. cereus*. In the WSF treatment, the decrease of final mite population was 1.5 times after both 1 and 5% diet enrichment of *B. cereus*.

## Discussion

### Proteolytic activities in *T. putrescentiae* feces supplemented by bacillolysins

The ascertained protease activities in mite feces and overall protein disappearance from the 2D-E pattern of control DDF extract compared to the 2D-E of feces extract demonstrated the powerful mite-bacterial digestive proteolytic activity in our experiment. Although DDF is made via the process of extrusion (Thompson, 2008), we were able identify some proteins, with highest success to *Triticum* α-AI, via the mass spectrometry approach. The digestion of the abundant α-AI is consistent with the fact that these proteinaceous inhibitors are low effective to mites (Hubert et al., 2014). The digestive pH in the gut of SPMs including *T. putrescentiae* ranges between 4.5 and 7.0 (Erban and Hubert, 2010b). Thus, the peaks of general and specific protease activities found in the mite gut pH range but not at alkaline pH can be considered the mite digestive enzymes. The results indicated that the protease activities detected were not only of mite origin but were influenced by the bacillolysins. The enzymatic activity at pH 6.75 was likely affected by neutral bacillolysins, and the activity measured at pH 8.0 was probably affected by the alkaline bacillolysins. Similarly, in a study by Ortego et al. (2000), the general and specific protease activities at pH 7.0, 7.5, 9.5, and 10 in the mite feces could be influenced by the bacterial proteases (Erban and Hubert, 2010a). The activities at pH 5.75 corresponded mostly to the digestive proteases of the mite, and fit best with pH in the middle or posterior midgut (Erban and Hubert, 2010b). The Tyr p3 trypsin activity in feces could correspond to the peak of general protease activity at pH 5.75. Such optimal enzyme activity suggests activation in the middle or posterior midgut. This is supported by the fact that trypsin Der f3 is localized in the postcolon (posterior midgut) of *D. farinae* (Zhan et al., 2010) and that trypsin activity is localized *in vivo* in the colon and postcolon but not the anterior midgut of *L. destructor* (Erban and Hubert, 2011). Herein, Tyr p3 was detected at different positions of the 2D-E. This result indicated presence of zymogen at basic pI, and mature-enzyme form and enzyme fragment at acidic pI. However, the activation of protease in the mite gut needs to be investigated further.

### Detection of *T. putrescentiae* trypsin, but almost lack of mite cathepsin B

Cysteine protease activity is of great interest in the acaridid mites because those enzymes are abundant in feces, and they are the most studied Grp 1 allergens of HDMs (Tovey et al., 1981; Thomas et al., 2010; Erban and Hubert, 2015). It was indicated that the cathepsin B-like cysteine protease activity corresponding to Grp 1 allergens of HDMs is low abundance in the feces/gut digestive pH of *T. putrescentiae* (Ortego et al., 2000; Erban and Hubert, 2010a). In this study, we confirmed that the cathepsin B-like activity in *T. putrescentiae* feces was only low (at the pH 6.75). Despite the zymographic analysis indicating specific cathepsin B-like activity, it needs to be determined whether the proteases are of mite or symbiont origin. Our results suggest that *T. putrescentiae* does not share digestive/allergen importance with the gut derived Grp 1 allergens of *Dermatophagoides* sp. This is different from the Grp 3 mite allergens. The presence and important abundance of Der f3 was confirmed in feces of *D. farinae* (Erban and Hubert, 2015), and here we demonstrated the presence of Tyr p3 in the feces of *T. putrescentiae*. The difference exists in comparison these mites with *D. pteronyssinus* because this mite almost lacks Der p3 in feces (Erban et al., under review). Der p3 was detected in feces of *D. pteronyssinus* only at a basic pI (Erban et al., under review). It is similar to herein suggested zymogen of Tyr p3 detected at the basic pI of the 2D-E. Overall, the result raises the possibility of Tyr p3 allergenic importance and suggests that it contributes to protein digestion in the mite.

### Chymotrypsin-like activity in feces extract

The chymotrypsin Grp 6 HDM allergens were the other candidates for protease activities/allergens in *T. putrescentiae* feces. We confirmed the presence of chymotrypsin-like activity in the feces using both enzymatic and zymographic analysis. However, the chymotrypsin activity was relatively low compared to trypsin activity. The digestive potential of chymotrypsin-like activity has been suggested similar to trypsin in *T. putrescentiae* (Ortego et al., 2000). Although our enzyme results partially support this, the lack of *T. putrescentiae* or similar chymotrypsin sequence records in the current version of Genbank did not allow us to confirm or exclude this suggestion by MS/MS identification.

### Symbiotic *Bacillus cereus* bacterium producing exo-proteases and exo-chitinases

The detection of numerous spots in 2D-E assigned with highest Mascot scores to *B. cereus*/*thuringiensis*/*anthracis* indicated the presence of a *B. cereus* group symbiont. The analysis of 16S rRNA from the bacterium isolated from the feces confirmed the identity to the *B. cereus* group, and the analysis of the *motB* gene confirmed this result. Previously, cultivation and cultivation independent approaches indicated *Bacillus* association with laboratory populations of *T. putrescentiae* (Hubert et al., 2012b). The mite-bacteria association was not disrupted by the addition of antibiotics to the diet (Kopecky et al., 2014). These findings, together with results of this study, indicate a strong association between *Bacillus* and the laboratory *T. putrescentiae* population. The isolation of the bacterium from the feces demonstrated the dominance of the bacterium, which was the only colony morphotype among the isolates. It similarly indicates the great abundance of *B. cereus* proteins in the 2D-E of the feces. Therefore, it appears that our isolated bacterium is the mite-relevant symbiont that suppresses other bacteria. We showed long-term association of the mite with this bacterial species because the bacterium has been present in our *T. putrescentiae* culture for ca. 10 years (Hubert et al., 2012a; Kopecky et al., 2014). Moreover, the first proteomic *B. cereus* detection via the 2D-E-MS/MS was in 2011, and 16S rRNA identification and isolation were performed in 2015.

### Mutualism between *T. putrescentiae* cultured on dry dog food and *Bacillus cereus*

Bacterial symbionts are important for metazoans. They cover the whole spectrum from beneficial mutualists to infectious, disease-causing pathogens. Benefits that the host derives from mutualists are diverse and include extracting essential nutrients from food (Nicholson et al., 2012; Staubach et al., 2013). *T. putrescentiae* can benefit from the *Bacillus* bacterial cells; however, previously was demonstrated for the model bacterium *Micrococcus lysodeikticus* as mite food enrichment (5 w/w) that this mite was not influenced by this treatment and bacteriolytic activity was relatively low compared to other species of mites (Erban and Hubert, 2008). *Bacillus* species are known to produce a wide variety of extracellular enzymes, and the enzymes are of industrial interest (Priest, 1977; Gupta et al., 2002). Two major types of proteases are secreted by bacilli, a subtilisin or alkaline protease and a thermolysin-like metalloprotease or neutral protease (Morihara et al., 1968; Feder et al., 1971; Holmquist and Vallee, 1976; Vasantha et al., 1984; Sidler et al., 1986). In addition, the zinc neutral proteases have demonstrated esterase activity (Holmquist and Vallee, 1976). The events that we detected for both of these bacterial exo-proteases in the feces extract of *T. putrescentiae* cultured on DDF mean that the *B. cereus* significantly contributes to digestion of protein and fat. Thus, the mite benefits from the predigested substrate. In addition to providing nutrition for the mite, bacillolysins can negatively impact the mite because they are virulence factors of diverse bacterial pathogens that are capable of mediating strong immune responses in invertebrate hosts (Altincicek et al., 2007). The negative impact on fitness of mite demonstrated the population growth tests. The excessive addition of *B. cereus* to diet highly suppressed population growth of *T. putrescentiae*.

The next detected exo-enzyme of the bacterium was an exo-chitinase. The exo-chitinases are known extracellular enzymes of *Bacillus* (Priest, 1977; Wang et al., 2001). The substrate for chitinases is chitin, a major component of most fungal cell walls, insect exoskeletons, and the shells of crustaceans (Flach et al., 1992; Lee et al., 2008; Martinez et al., 2014). In our experiment, the available substrate for the bacterial exo-chitinases in the mite culture was the mite bodies and the exuviae or peritrophic membranes that cover the fecal pellets (Lee et al., 2008; Sobotnik et al., 2008b; Martinez et al., 2014). The fungal cells were not a suggested substrate because we did not detect or observe fungi, the growth of which could be regulated by the bacterial exo-enzymes (Podile et al., 1985; Podile and Prakash, 1996; Manjula and Podile, 2005). Thus, the bacterium contributed to degradation of residues of the mites and was a possible regulator of fungi. The bacterial exo-chitinases can disrupt the feces by degrading the chitin fibrils in the peritrophic membrane, thereby releasing allergens from the feces.

### Medical/veterinary aspects of detected mite proteins/allergens

*T. putrescentiae* is the common species found in agricultural settings and in homes, where it serves as important source of allergens for humans and animals (van Hage-Hamsten and Johansson, 1998; Bravo et al., 1999; Elder et al., 2012). Just the chitin present in the mite bodies and the peritrophic membrane surrounding feces (Lee et al., 2008; Sobotnik et al., 2008b; Martinez et al., 2014) is capable of producing an allergic response (Mack et al., 2015). The mite bodies and feces contain allergens; however, because mites spread the feces, the feces-containing gut derived proteins are considered the most important source of allergens (Tovey et al., 1981; Erban and Hubert, 2015). In the feces extract of *T. putrescentiae*, we detected three allergens, Tyr p3, Tyr p13, and ferritin (Grp 30), which are current (23/01/2016; www.allergen.org) members of the mite allergenic groups approved by IUIS. In addition, we detected spots of low-molecular weight ubiquitins. In *Blomia tropicalis*, was observed 50% IgE binging in immunoblots to the 6.5 kDa protein that had 98% identity to several polyubiquitins (Mercado et al., 2003). Moreover, ubiquitin was identified as one of the proteins responsible for mite-shrimp cross-reactivity (Gámez et al., 2014). The allergenic potential of mite ubiquitin and the fact that ubiquitin spots in the 2D-E of the *T. putrescentiae* feces were relatively strong suggest that ubiquitin should be the focus of future studies of mite allergens.

### Medical/veterinary importance of *Bacillus cereus* and its exo-enzymes

The innate immune system is mediated by a variety of cells and molecular mechanisms that generically recognize pathogens (Thomas et al., 2010). Pathogen recognition is mediated by specific pathogen recognition receptors (PRRs) that respond to pathogen associated molecular patterns (PAMPs). Prolonged epithelial PRR activation by the allergens themselves or by microbial contaminating PAMPs represents one of the key steps in the Th2 cell sensitization process, which is connected to allergies (Lambrecht and Hammad, 2012). PAMPs include bacteria, fungi and their compounds, such as LPS (lipopolysaccharides, endotoxin), β-glucans and chitins. These compounds are abundant in house dust (Douwes et al., 2000; Jacquet, 2013). Hence, the high-abundance bacterium and its compounds represent a serious danger for the recipient immune system.

*B. cereus* is an aerobic endospore-forming, gram-positive rod that is widely distributed in the environment, including soil, dust, mud, decaying organic matter, fresh and marine waters, vegetables and fomites (Drobniewski, 1993; Fekete, 2010; Ribeiro et al., 2010). It is also well adapted for growth in the intestinal tract of arthropods and mammals (Margulis et al., 1998; Swiecicka and Mahillon, 2006; Stenfors Arnesen et al., 2008; Bottone, 2010). It easily spreads in food, where may cause an emetic or diarrheal type of food-associated illness. The emetic disease is a food intoxication caused by cereulide, a small ring-formed dodecadepsipeptide. Due to the impact of *B. cereus* on health, it is commonly considered a volatile human pathogen (Häggblom et al., 2002; Stenfors Arnesen et al., 2008; Bottone, 2010). Other toxins produced during *B. cereus* growth constitute phospholipases, proteases, or hemolysins. These toxins may also contribute to the pathogenicity of *B. cereus* in nongastrointestinal disease (Drobniewski, 1993). In one case, the proteolytic enzymes of mites and cockroaches were described as having dual allergen functions, including one function that affects the epidermal barrier and stimulates protease-activated receptor 2 (PAR-2; Kondo et al., 2004; Jeong et al., 2008; Roelandt et al., 2008). This protease likely increases allergenicity for the bacillolysins of the bacterium, especially if mite proteases are also present, as Tyr p3 is in our case. If other proteins of the bacterium represent health dangers for mammals, this needs to be investigated further. At least our detected *B. cereus* chitinases constitute a risk, and the supposition is that chitinases are used by pathogens (mainly protozoan or metazoan) and cause animal and human diseases (Hamid et al., 2013). The chitinases Der f15 and Der f18 of *D. farinae* are recognized as allergens for dogs (McCall et al., 2001; Weber et al., 2003). Thus, there is suspicion that there can be some similarity between bacterial and mite chitinases, leading to allergen cross-reactivity.

The mite is apparently capable of serving as the carrier of the bacterium and/or its putative toxins to the foods or dust in homes, similar to mycotoxin-producing fungi (Armitage and George, 1986; Hubert et al., 2004). The mite feces are rich in mite allergens, and the peritrophic membrane of the feces contains chitin. Bacterial cells, spores, enzymes, and toxins represent a dangerous cocktail for humans and animals after ingestion or inhalation. Our results indicate that the association of the *B. cereus* group bacterium with the cosmopolitan mite *T. putrescentiae* is of great medical and veterinary importance. Additional molecular, enzymatic, and protein immunoreactivity characterization of our bacterial isolates will help to identify the particular health risks for humans and animals.

### Mite-*Bacillus cereus* association: Association from soil to the synanthropic environment

There is interest in fungal or bacterial associations because mites interact with microbes. The nutritional importance of fungi and/or bacteria leads to increased mite populations (Sinha, 1966; Erban and Hubert, 2008). The synanthropic acaridid mites originally inhabited the soil and migrated into human habitats from the nests of birds and mammals during the Neolithic revolution (OConnor, 1979, 1982, 1984). It is thought that mites, including *T. putrescentiae*, feed on decomposing tissues in the soil and make use of the fungi and bacteria growing there (Smrz and Catska, 1989, 2010; Erban and Hubert, 2008). In addition, the bacteria in the mite gut are thought to contribute to digestion of the chitin from fungi by exoenzymes (Smrz, 2003; Smrz and Catska, 2010). An increase in diversity within Bacillacae in *T. putrescentiae* was observed when the diet of the laboratory strains was changed to fungi (Hubert et al., 2012b). We suggest that the symbiotic interaction identified here between the mite and *B. cereus* also occurs in the soil, where those bacteria comprise soil-dwelling saprophytes (Vilain et al., 2006; Didelot et al., 2009). However, the bacterium apparently limits fungal growth (Vilain et al., 2006) because we did not observe fungal growth in our culture of *T. putrescentiae* on DDF. The *B. cereus* clade is ubiquitous, so the mite can acquire different strains from the environment. Another possibility is that the mite co-exists with the bacterial strain for a long time, and the bacterial abundance increases with an appropriate food source, such as the DDF used in this study. However, here we observed the suppressive effect of *B. cereus* addition to the diet (1 and 5%) on population growth of *T. putrescentiae*. The suppressive effect of *Bacillus sphaericus* to *Dermatophagoides pteronyssinus* development (Saleh et al., 1991) and *Bacillus thuringiensis* var *tenebrionis* to *T. putrescentianae* population growth is well documented (Erban et al., 2009b). Here we suggest that *B. cereus* exo-chitinases are responsible for growth suppression through damage of perithrophic matrix or mite body surface. In *Acarus siro* the addition of chitinase in the range 0.5–50 mg/g to the diet suppressed population growth (Sobotnik et al., 2008b). However, previously in case of *T. putrescentiae* we did not find difference in final population density between chitinase additive to diet (12.5 mg/g) and control diet (Stara et al., 2010). It indicates that the exo-chitinases can differ in their mode of action between mites and increasing concertation should be responsible for suppressive effect.

The need for mite-microbial interactions, whether fungal or bacterial, has been suggested. Analogical situation has been reported for *Aspergillus penicillioides* and *D. pteronyssinus* (de Saint Georges-Gridelet, 1987; Hay et al., 1993). Although the fungus suppresses *D. pteronyssinus*, the fungus-free mites have much lower fitness than those from the cultures with fungus (Hay et al., 1993). Thus, the mite-*B. cereus* symbiosis can be beneficial-suppresive. For mites such mite-microbial interactions may promote effective utilization of nutrients from keratin and collagen by the mites. Mite extracts show keratinolytic and collagenolytic activities and are highly active at basic pH (10), which facilitates partial hydrolysis of these substrates (Erban and Hubert, 2010a). In the wild, the degradation of keratin-rich substrates, such as skin, hair, nails, and feathers, is caused by microscopic fungi and bacteria including *B. cereus* (Cardamone, 2002; Muhsin and Hadi, 2002; Gunderson, 2008; Adigüzel et al., 2009). *B. cereus* strains were found to produce keratinases (Adigüzel et al., 2009). The bacillolysins detected here can be effective at hydrolyzing keratin and collagen substrates. Thus, if the mite carries the bacterium from the DDF, the *T. putrescentiae*-*B. cereus* interaction can continue on skin derivatives. It is in agreement with mites harbor a variety of bacterial species often associated with human skin (Tang et al., 2013).

### Veterinary and medical significance

The DDF infested with *T. putrescentiae* and associated bacterium *B. cereus* represents serious health risks that arise mainly from mite allergens and toxins of bacterium. The *B. cereus* exo-proteases can comprise a large part of the proteases in mite extracts, and thereby contribute to allergy.

## Author contributions

TE conceived and designed the study. TE wrote the main manuscript. DR and TE prepared samples, and performed enzyme and gel-based analysis. BH and JH performed isolation and identification of *B. cereus*. KH performed MS/MS identification. TE, DR, BH, and JH prepared figures and tables. TE and JH provided grants.

### Conflict of interest statement

The authors declare that the research was conducted in the absence of any commercial or financial relationships that could be construed as a potential conflict of interest.
