# Supplementary material for: Feces Derived Allergens of Tyrophagus putrescentiae Reared on Dried Dog Food and Evidence of the Strong Nutritional Interaction between the Mite and Bacillus cereus Producing Protease Bacillolysins and Exo-chitinases
Source: Front Physiol. 2016 Feb 24;7:53. doi: 10.3389/fphys.2016.00053 (PMC4764834; doi:10.3389/fphys.2016.00053)
Supplement: Supplementary file 1 [file Table1.PDF]

Table supplement 1 – List of proteins and details of MS/MS protein identifications in *Tyrophagus putrescentiae* feces extract

Journal name: Frontiers in Physiology – Invertebrate Physiology

Article title: Feces derived allergens of *Tyrophagus putrescentiae* reared on dried dog food and evidence of the strong nutritional interaction between the mite and B*acillus cereus* producing protease bacillolysins and exo-chitinases

Author names: Tomas Erban*, Dagmar Rybanska, Karel Harant, Bronislava Hortova, Jan Hubert

*Corresponding author: Tomas ERBAN; Crop Research Institute; Biologically Active Substances in Crop Protection; Laboratory of Proteomics; Drnovska 507/73, Prague 6-Ruzyne; CZ-16106; Czech Republic

E-mail: arachnid@centrum.cz

A) List of proteins MS/MS identified in *Tyrophagus putrescentiae* feces extract

Spot No.	Result No.	Score	GI	Description [Taxonomy] of considered results	Mass	Expect	Queries Matched
**1a**	1.	378	gi|374073976	**Chain B, Structure Of Usp21 In Complex With Linear Diubiquitin-Aldehyde**	17235	3.7e-031	16
**1b**	1.	172	gi|228311825	**Chain A, Crystal Structure Of Linear Di-Ubiquitin**	17081	1.5e-010	13
**2**	1.	203	gi|223646258	**Ubiquitin [Salmo salar]**	21483	1.2e-013	11
**3**	1.	70	gi|30983942	**putative polyubiquitin [Gossypium barbadense]**	15022	2.4	6
**4**	1.	199	gi|51860756	**fatty acid-biding protein [Tyrophagus putrescentiae]**	14565	2.9e-013	10
	2.	113	gi|156938917	**allergen Tyr p 13 [Tyrophagus putrescentiae]**	14537	0.00012	7
**5**	1.	210	gi|374073976	**Chain B, Structure Of Usp21 In Complex With Linear Diubiquitin-Aldehyde**	17235	2.3e-014	11
**6**	1.	110	gi|1402737	**major cold-shock protein, partial [Bacillus cereus]**	5004	0.00023	4
**7**	1.	161	gi|30021641	**cold shock protein [Bacillus cereus ATCC 14579]**	7362	1.8e-009	7
**8**	1.	158	gi|30022927	**cold shock protein [Bacillus cereus ATCC 14579]**	7234	3.7e-009	5
**9**	1.	150	gi|66841026	**alpha-amylase inhibitor 0.19 [Triticum aestivum]**	13340	2.3e-008	9
**10a**	1.	108	gi|269999913	**ferritin 3 [Coffea arabica]**	15114	0.00037	8
**10b**	1.	141	gi|28630234	**ferritin heavy chain polypeptide 1 [Branchiostoma lanceolatum]**	19901	2.8e-007	6
	2.	127	gi|256079612	**ferritin [Schistosoma mansoni]**	21035	7.00E-06	7
	4.	96	gi|290020584	**ferritin [Coffea arabica]**	32304	0.0099	7
**10c**	1.	119	gi|28630234	**ferritin heavy chain polypeptide 1 [Branchiostoma lanceolatum]**	19901	4.4e-005	4
**11**	1.	122	gi|156124994	**Tyr p 3 allergen [Tyrophagus putrescentiae]**	20059	1.5e-005	5
**12**	1.	237	gi|75762754	**Nucleoside diphosphate kinase [Bacillus thuringiensis serovar israelensis ATCC 35646]**	19962	4.6e-017	14
**13**	1.	148	gi|423464038	**superoxide dismutase [Mn] 1 [Bacillus cereus BAG6O-1]**	22708	3.7e-008	16
**14a**	1.	123	gi|15149252	**triosephosphate isomerase B [Xiphophorus maculatus]**	26761	1.2e-005	9
**14b**	1.	90	gi|297529143	**ribonuclease HIII [Geobacillus sp. C56-T3]**	34420	0.033	17
**15a**	1.	148	gi|30264347	**superoxide dismutase [Bacillus anthracis str. Ames]**	22650	5.6e-008	12
**15b**	1.	145	gi|30264347	**superoxide dismutase [Bacillus anthracis str. Ames]**	22650	1.1e-007	13
**16**	1.	172	gi|448519843	**Sod2 mitochondrial Mn-containing superoxide dismutase [Candida orthopsilosis Co 90-125]**	22311	2.2e-010	9
**17**	1.	88	gi|308508467	**hypothetical protein CRE_08947 [Caenorhabditis remanei]**	117937	0.04	38
**18a**	1.	102	gi|308508467	**hypothetical protein CRE_08947 [Caenorhabditis remanei]**	117937	0.0015	41
	2.	77	gi|88801757	**Secretion protein HlyD [Polaribacter irgensii 23-P]**	41353	0.5	14
**18b**	1.	90	gi|32473681	**hypothetical protein RB5404 [Rhodopirellula baltica SH 1]**	41678	0.036	16
**19a**	1.	399	gi|156124994	**Tyr p 3 allergen [Tyrophagus putrescentiae]**	20059	2.9e-033	7
**19b**	1.	188	gi|156124994	**Tyr p 3 allergen [Tyrophagus putrescentiae]**	20059	5.6e-012	8
**20a**	1.	210	gi|156124994	**Tyr p 3 allergen [Tyrophagus putrescentiae]**	20059	2.3e-014	10
**20b**	1.	147	gi|156124994	**Tyr p 3 allergen [Tyrophagus putrescentiae]**	20059	7.0e-008	6
	2.	92	gi|325108307	**type VI secretion ATPase, ClpV1 family [Planctomyces brasiliensis DSM 5305]**	96614	0.02	23
**20c**	1.	118	gi|156124994	**Tyr p 3 allergen [Tyrophagus putrescentiae]**	20059	5.6e-005	7
**21**	1.	95	gi|401626492	**hbt1p [Saccharomyces arboricola H-6]**	113679	0.0075	34
	2.	75	gi|304396978	**conserved hypothetical protein [Pantoea sp. aB]**	141476	0.73	29
**22**	1.	200	gi|196039924	**alkaline serine protease, subtilase family [Bacillus cereus NVH0597-99]**	42448	2.3e-013	11
**23**	1.	425	gi|196039924	**alkaline serine protease, subtilase family [Bacillus cereus NVH0597-99]**	42448	7.3e-036	17
**24**	1.	144	gi|218901795	**neutral protease Npr599 [Bacillus cereus AH820]**	60941	9.2e-008	16
**25**	1.	413	gi|218901795	**neutral protease Npr599 [Bacillus cereus AH820]**	60941	1.2e-034	24
**26**	1.	550	gi|229188808	**Bacillolysin [Bacillus cereus ATCC 10876]**	60929	2.3e-048	27
**27a**	1.	217	gi|229188808	**Bacillolysin [Bacillus cereus ATCC 10876]**	60929	4.6e-015	18
**27b**	1.	187	gi|487962439	**bacillolysin [Bacillus cereus]**	62720	7.4e-017	17
**28**	2.	344	gi|229191847	**Extracellular exochitinase [Bacillus cereus ATCC 10876]**	37774	9.2e-028	20
**29**	1.	336	gi|156124994	**Tyr p 3 allergen [Tyrophagus putrescentiae]**	20059	5.8e-027	8
**30**	1.	145	gi|229191670	**Extracellular solute-binding protein family 5 [Bacillus cereus ATCC 10876]**	63482	7.3e-008	23
**31**	10.	99	gi|229191670	**Extracellular solute-binding protein family 5 [Bacillus cereus ATCC 10876]**	63482	0.0031	20
**32**	1.	175	gi|229188566	**1-pyrroline-5-carboxylate dehydrogenase [Bacillus cereus ATCC 10876]**	56445	7.3e-011	26
**33**	1.	151	gi|229188566	**1-pyrroline-5-carboxylate dehydrogenase [Bacillus cereus ATCC 10876]**	56445	1.8e-008	23

B) Details of MS/MS protein identifications in *Tyrophagus putrescentiae* feces extract

Spot No.	Result No.	Score	GI	Description [Taxonomy] - *results in bold were considered*	Mass	Expect	Queries Matched	Observed	Mr(expt)	Mr(calc)	ppm	Start	End	Miss	Ions	Peptide
**1a**	**1.**	378	gi|374073976	**Chain B, Structure Of Usp21 In Complex With Linear Diubi**	17235	3.7e-031	16	717.3478	716.3405	716.3453	-6.68	125	130	0	---	K.QLEDGR.T
								765.4180	764.4107	764.4255	-19.30	1	6	0	---	-.MQIFVK.T
								781.4205	780.4132	780.4204	-9.19	1	6	0	---	-.MQIFVK.T + Oxidation (M)
								1039.5237	1038.5164	1038.5094	6.73	34	42	0	35	K.EGIPPDQQR.L
								1039.5237	1038.5164	1038.5094	6.75	110	118	0	---	K.EGIPPDQQR.L
								1067.6313	1066.6240	1066.6135	9.89	140	148	0	---	K.ESTLHLVLR.L
								1067.6314	1066.6241	1066.6135	9.93	64	72	0	48	K.ESTLHLVLR.L
								1081.5552	1080.5479	1080.5451	2.58	55	63	0	62	R.TLSDYNIQK.E
								1081.5552	1080.5479	1080.5451	2.59	131	139	0	---	R.TLSDYNIQK.E
								1475.7659	1474.7586	1474.7966	-25.77	75	87	1	---	R.GHMQIFVKTLTGK.T + Oxidation (M)
								1523.7925	1522.7852	1522.7740	7.39	30	42	1	65	K.IQDKEGIPPDQQR.L
								1523.7925	1522.7852	1522.7740	7.40	106	118	1	---	K.IQDKEGIPPDQQR.L
								1782.9944	1781.9871	1781.8319	87.1	125	139	1	---	K.QLEDGRTLSDYNIQK.E + 3 Deamidated (NQ)
								1787.9332	1786.9259	1786.9200	3.31	88	103	0	---	K.TITLEVEPSDTIENVK.A
								1787.9332	1786.9260	1786.9200	3.32	12	27	0	72	K.TITLEVEPSDTIENVK.A
								2130.1514	2129.1441	2129.1480	-1.84	131	148	1	---	R.TLSDYNIQKESTLHLVLR.L
	**2.**	376	gi|226477466	polyubiquitin [Schistosoma japonicum]	19260											
	**3.**	372	gi|223646258	Ubiquitin [Salmo salar]	21483											
	**4.**	370	gi|26353460	unnamed protein product [Mus musculus]	17214											
	**5.**	369	gi|90075190	unnamed protein product [Macaca fascicularis]	17892											
	**6.**	369	gi|355753795	Polyubiquitin-C [Macaca fascicularis]	17261											
	**7.**	368	gi|1805696	polyubiquitin [Ceratitis capitata]	15038											
	**8.**	368	gi|361130305	Chain A, Crystal Structure Of The Mouse Hoil1-L-Nzf In Comp	17101											
	**9.**	368	gi|7799051	ubiquitin [Cyanidium caldarium]	17293											
	**10.**	368	gi|395836339	PREDICTED: polyubiquitin-B [Otolemur garnettii]	17261											
	**11.**	368	gi|326931517	PREDICTED: polyubiquitin-B-like isoform 1 [Meleagris gallopa	17264											
	**12.**	368	gi|256079432	ubiquitin (ribosomal protein L40) [Schistosoma mansoni]	17214											
	**13.**	368	gi|226484009	ubiquitin C [Schistosoma japonicum]	17214											
	**14.**	368	gi|403221030	ubiquitin [Theileria orientalis strain Shintoku]	17464											
	**15.**	367	gi|71033201	ubiquitin [Theileria parva strain Muguga]	17479											
	**16.**	367	gi|28189917	similar to polyubiquitin [Bos taurus]	19318											
	**17.**	366	gi|84998672	ubiquitin [Theileria annulata]	17935											
	**18.**	366	gi|350537349	putative ubiquitin C variant 4 [Taeniopygia guttata]	17215											
	**19.**	366	gi|209875527	ubiquitin family protein [Cryptosporidium muris RN66]	17462											
	**20.**	364	gi|428672795	ubiquitin family member protein [Babesia equi]	17555											
	**21.**	363	gi|149063235	rCG21224 [Rattus norvegicus]	18359											
	**22.**	363	gi|195491526	GE20668 [Drosophila yakuba]	8872											
	**23.**	362	gi|1321735	ubiquitin [Carabus alpestris]	15669											
	**24.**	362	gi|440891703	Polyubiquitin-B [Bos grunniens mutus]	8819											
	**25.**	361	gi|56199552	ubiquitin [Culicoides sonorensis]	18776											
	**26.**	361	gi|62079638	ubiquitin [Oreochromis mossambicus]	17908											
	**27.**	360	gi|228311825	Chain A, Crystal Structure Of Linear Di-Ubiquitin	17081											
	**28.**	360	gi|54300702	ubiquitin C splice variant [Homo sapiens]	17142											
	**29.**	359	gi|54610299	polyubiquitin-like protein [Schistosoma japonicum]	17621											
	**30.**	359	gi|324525461	Polyubiquitin, partial [Ascaris suum]	29349											
	**31.**	359	gi|324525469	Polyubiquitin, partial [Ascaris suum]	29377											
	**32.**	358	gi|323714499	Chain A, Crystal Structure Of Human Ubiquitin In A New Cryst	8176											
	**33.**	357	gi|225733991	Chain A, Nemo Cozi Domain Incomplex With Diubiquitin In P2	17245											
	**34.**	357	gi|163575	polyubiquitin, partial [Bos taurus]	18477											
	**35.**	356	gi|372467011	Chain A, Crystal Structure Of A Linear-Specific Ubiquitin Fab	19263											
	**36.**	356	gi|225715160	Ubiquitin [Esox lucius]	10523											

	**37.**	356	gi|197129059	putative ubiquitin C variant 4 [Taeniopygia guttata]	21291											
	**38.**	356	gi|339233028	ubiquitin family protein [Trichinella spiralis]	23043											
	**39.**	356	gi|14250448	Ubc protein [Mus musculus]	22822											
	**40.**	355	gi|229532	ubiquitin	8446											
	**41.**	355	gi|290560476	Chain F, Crystal Structure Of Human Stam1 Vhs Domain In Co	8289											
	**42.**	355	gi|109157803	Chain A, Crystal Structure Of The 9-10 8 Glycine Insertion Mut	9016											
	**43.**	355	gi|325302604	TPA_exp: ubiquitin [Amblyomma variegatum]	22017											
	**44.**	355	gi|28189839	similar to polyubiquitin [Bos taurus]	17644											
	**45.**	355	gi|212851	ubiquitin polyprotein (heat shock related), partial [Gallus gallus]	17648											
	**46.**	355	gi|78099807	RecName: Full=Ubiquitin	7199											
	**47.**	354	gi|195587566	GD13313 [Drosophila simulans]	21780											
	**48.**	354	gi|195190296	GL14086 [Drosophila persimilis]	8890											
	**49.**	353	gi|350537541	putative ubiquitin C variant 9 [Taeniopygia guttata]	25815											
	**50.**	353	gi|119624911	ubiquitin B, isoform CRA_e [Homo sapiens]	16906											
**1b**	**1.**	172	gi|228311825	**Chain A, Crystal Structure Of Linear Di-Ubiquitin**	17081	1.5e-010	13	1039.4032	1038.3959	1038.5094	-109.28	110	118	0	---	K.EGIPPDQQR.L
								1040.3878	1039.3805	1039.4934	-108.61	110	118	0	---	K.EGIPPDQQR.L + Deamidated (NQ)
								1041.3765	1040.3692	1040.4774	-104.00	110	118	0	---	K.EGIPPDQQR.L + 2 Deamidated (NQ)
								1067.5056	1066.4983	1066.6135	-107.96	140	148	0	---	K.ESTLHLVLR.L
								1067.5056	1066.4983	1066.6135	-107.95	64	72	0	38	K.ESTLHLVLR.L
								1524.6619	1523.6546	1523.7580	-67.82	106	118	1	---	K.IQDKEGIPPDQQR.L + Deamidated (NQ)
								1525.6534	1524.6461	1524.7420	-62.86	106	118	1	---	K.IQDKEGIPPDQQR.L + 2 Deamidated (NQ)
								1525.6534	1524.6462	1524.7420	-62.84	30	42	1	41	K.IQDKEGIPPDQQR.L + 2 Deamidated (NQ)
								1526.6433	1525.6360	1525.7260	-58.96	106	118	1	---	K.IQDKEGIPPDQQR.L + 3 Deamidated (NQ)
								1787.8196	1786.8123	1786.9200	-60.28	12	27	0	31	K.TITLEVEPSDTIENVK.A
								1787.8196	1786.8123	1786.9200	-60.27	88	103	0	---	K.TITLEVEPSDTIENVK.A
								2131.0654	2130.0581	2130.1321	-34.70	131	148	1	---	R.TLSDYNIQKESTLHLVLR.L + Deamidated (NQ)
								2131.0654	2130.0582	2130.1321	-34.69	55	72	1	16	R.TLSDYNIQKESTLHLVLR.L + Deamidated (NQ)
	**2.**	170	gi|82622379	unknown [Theileria lestoquardi]	17936											
	**3.**	169	gi|163575	polyubiquitin, partial [Bos taurus]	18477											
	**4.**	169	gi|361130305	Chain A, Crystal Structure Of The Mouse Hoil1-L-Nzf In Comp	17101											
	**5.**	169	gi|374073976	Chain B, Structure Of Usp21 In Complex With Linear Diubiquit	17235											
	**6.**	169	gi|71033201	ubiquitin [Theileria parva strain Muguga]	17479											
	**7.**	169	gi|7799051	ubiquitin [Cyanidium caldarium]	17293											
	**8.**	169	gi|395836339	PREDICTED: polyubiquitin-B [Otolemur garnettii]	17261											
	**9.**	169	gi|326931517	PREDICTED: polyubiquitin-B-like isoform 1 [Meleagris gallopa	17264											
	**10.**	169	gi|256079432	ubiquitin (ribosomal protein L40) [Schistosoma mansoni]	17214											
	**11.**	169	gi|226484009	ubiquitin C [Schistosoma japonicum]	17214											
	**12.**	169	gi|355753795	Polyubiquitin-C [Macaca fascicularis]	17261											
	**13.**	169	gi|225733991	Chain A, Nemo Cozi Domain Incomplex With Diubiquitin In P2	17245											
	**14.**	169	gi|84998672	ubiquitin [Theileria annulata]	17935											
	**15.**	169	gi|403221030	ubiquitin [Theileria orientalis strain Shintoku]	17464											
	**16.**	168	gi|350537349	putative ubiquitin C variant 4 [Taeniopygia guttata]	17215											
	**17.**	168	gi|28189839	similar to polyubiquitin [Bos taurus]	17644											
	**18.**	168	gi|212851	ubiquitin polyprotein (heat shock related), partial [Gallus gallus]	17648											
	**19.**	168	gi|209875527	ubiquitin family protein [Cryptosporidium muris RN66]	17462											
	**20.**	168	gi|372467011	Chain A, Crystal Structure Of A Linear-Specific Ubiquitin Fab F	19263											
	**21.**	167	gi|342326368	ubiquitin [Cherax quadricarinatus]	11182											
	**22.**	167	gi|226477466	polyubiquitin [Schistosoma japonicum]	19260											
	**23.**	167	gi|428672795	ubiquitin family member protein [Babesia equi]	17555											
	**24.**	166	gi|302393789	RecName: Full=Polyubiquitin; Contains: RecName: Full=Ubiqui	18789											
	**25.**	166	gi|3954791	unnamed protein product [Gallus gallus]	17738											
	**26.**	165	gi|56199552	ubiquitin [Culicoides sonorensis]	18776											
	**27.**	165	gi|78099807	RecName: Full=Ubiquitin	7199											
	**28.**	164	gi|223646258	Ubiquitin [Salmo salar]	21483											
	**29.**	163	gi|385881402	polyubiquitin-B, partial [Anticola delacruzi]	17432											
	**30.**	163	gi|14250448	Ubc protein [Mus musculus]	22822											
	**31.**	163	gi|28189917	similar to polyubiquitin [Bos taurus]	19318											

	**32.**	162	gi|388571222	ubiquitin [Ostrea edulis]	17276											
	**33.**	162	gi|228069369	ubiquitin [Oncorhynchus tshawytscha]	7554											
	**34.**	162	gi|90075190	unnamed protein product [Macaca fascicularis]	17892											
	**35.**	162	gi|339233028	ubiquitin family protein [Trichinella spiralis]	23043											
	**36.**	162	gi|54300702	ubiquitin C splice variant [Homo sapiens]	17142											
	**37.**	162	gi|325302604	TPA_exp: ubiquitin [Amblyomma variegatum]	22017											
	**38.**	162	gi|114152964	ubiquitin/40S ribosomal protein S27a fusion protein [Argas mon	26778											
	**39.**	161	gi|226484025	ubiquitin C [Schistosoma japonicum]	25744											
	**40.**	161	gi|197129059	putative ubiquitin C variant 4 [Taeniopygia guttata]	21291											
	**41.**	160	gi|90076064	unnamed protein product [Macaca fascicularis]	23571											
	**42.**	160	gi|226473064	polyubiquitin [Schistosoma japonicum]	21907											
	**43.**	160	gi|29425820	polyubiquitin [Anas platyrhynchos]	13038											
	**44.**	160	gi|402232869	ribosomal protein S27-3, partial [Salvelinus fontinalis]	14447											
	**45.**	160	gi|48425521	Chain B, Tsg101(Uev) Domain In Complex With Ubiquitin	8540											
	**46.**	160	gi|71042034	Chain A, X-Ray Crystal Structure Of A Chemically Synthesized	8542											
	**47.**	160	gi|116488178	polyubiquitin [Scophthalmus maximus]	8429											
	**48.**	160	gi|195587566	GD13313 [Drosophila simulans]	21780											
	**49.**	160	gi|1805696	polyubiquitin [Ceratitis capitata]	15038											
	**50.**	160	gi|339232982	ubiquitin family protein [Trichinella spiralis]	12454											
2	**1.**	203	gi|223646258	**Ubiquitin [Salmo salar]**	21483	1.2e-013	11	1039.5068	1038.4995	1038.5094	-9.52	148	156	0	---	K.EGIPPDQQR.L
								1039.5068	1038.4996	1038.5094	-9.49	72	80	0	16	K.EGIPPDQQR.L
								1067.6190	1066.6117	1066.6135	-1.65	178	186	0	---	K.ESTLHLVLR.L
								1067.6190	1066.6117	1066.6135	-1.63	102	110	0	26	K.ESTLHLVLR.L
								1523.7654	1522.7581	1522.7740	-10.41	68	80	1	45	K.IQDKEGIPPDQQR.L
								1523.7654	1522.7581	1522.7740	-10.40	144	156	1	---	K.IQDKEGIPPDQQR.L
								1787.9155	1786.9082	1786.9200	-6.60	126	141	0	---	K.TITLEVEPSDTIENVK.A
								1787.9155	1786.9083	1786.9200	-6.58	50	65	0	33	K.TITLEVEPSDTIENVK.A
								2130.1255	2129.1182	2129.1480	-14.00	169	186	1	---	R.TLSDYNIQKESTLHLVLR.L
								2388.1372	2387.1299	2387.2683	-57.96	44	65	1	---	K.LSLTGKTITLEVEPSDTIENVK.A + Deamidated (NQ)
								2388.1372	2387.1299	2387.2683	-57.95	44	65	1	---	K.LSLTGKTITLEVEPSDTIENVK.A + Deamidated (NQ)
	**2.**	196	gi|228311825	Chain A, Crystal Structure Of Linear Di-Ubiquitin	17081											
	**3.**	195	gi|395536787	PREDICTED: polyubiquitin-C-like, partial [Sarcophilus harrisii]	27373											
	**4.**	194	gi|355753795	Polyubiquitin-C [Macaca fascicularis]	17261											
	**5.**	193	gi|163575	polyubiquitin, partial [Bos taurus]	18477											
	**6.**	193	gi|361130305	Chain A, Crystal Structure Of The Mouse Hoil1-L-Nzf In Comp	17101											
	**7.**	193	gi|374073976	Chain B, Structure Of Usp21 In Complex With Linear Diubiquit	17235											
	**8.**	193	gi|7799051	ubiquitin [Cyanidium caldarium]	17293											
	**9.**	193	gi|395836339	PREDICTED: polyubiquitin-B [Otolemur garnettii]	17261											
	**10.**	193	gi|326931517	PREDICTED: polyubiquitin-B-like isoform 1 [Meleagris gallopa	17264											
	**11.**	193	gi|256079432	ubiquitin (ribosomal protein L40) [Schistosoma mansoni]	17214											
	**12.**	193	gi|226484009	ubiquitin C [Schistosoma japonicum]	17214											
	**13.**	193	gi|54300702	ubiquitin C splice variant [Homo sapiens]	17142											
	**14.**	193	gi|225733991	Chain A, Nemo Cozi Domain Incomplex With Diubiquitin In P2	17245											
	**15.**	193	gi|403221030	ubiquitin [Theileria orientalis strain Shintoku]	17464											
	**16.**	193	gi|71033201	ubiquitin [Theileria parva strain Muguga]	17479											
	**17.**	192	gi|372467011	Chain A, Crystal Structure Of A Linear-Specific Ubiquitin Fab F	19263											
	**18.**	192	gi|84998672	ubiquitin [Theileria annulata]	17935											
	**19.**	192	gi|350537349	putative ubiquitin C variant 4 [Taeniopygia guttata]	17215											
	**20.**	192	gi|209875527	ubiquitin family protein [Cryptosporidium muris RN66]	17462											
	**21.**	191	gi|28189839	similar to polyubiquitin [Bos taurus]	17644											
	**22.**	191	gi|212851	ubiquitin polyprotein (heat shock related), partial [Gallus gallus]	17648											
	**23.**	191	gi|226477466	polyubiquitin [Schistosoma japonicum]	19260											
	**24.**	191	gi|428672795	ubiquitin family member protein [Babesia equi]	17555											
	**25.**	189	gi|3954791	unnamed protein product [Gallus gallus]	17738											
	**26.**	189	gi|302393789	RecName: Full=Polyubiquitin; Contains: RecName: Full=Ubiqui	18789											
	**27.**	189	gi|56199552	ubiquitin [Culicoides sonorensis]	18776											
	**28.**	188	gi|1805696	polyubiquitin [Ceratitis capitata]	15038											

	**29.**	187	gi|343960965	ubiquitin [Pan troglodytes]	19800											
	**30.**	186	gi|28189917	similar to polyubiquitin [Bos taurus]	19318											
	**31.**	185	gi|14250448	Ubc protein [Mus musculus]	22822											
	**32.**	185	gi|339233028	ubiquitin family protein [Trichinella spiralis]	23043											
	**33.**	185	gi|325302604	TPA_exp: ubiquitin [Amblyomma variegatum]	22017											
	**34.**	184	gi|197129059	putative ubiquitin C variant 4 [Taeniopygia guttata]	21291											
	**35.**	184	gi|440894648	Polyubiquitin-C, partial [Bos grunniens mutus]	11392											
	**36.**	184	gi|1321735	ubiquitin [Carabus alpestris]	15669											
	**37.**	184	gi|149063235	rCG21224 [Rattus norvegicus]	18359											
	**38.**	184	gi|385881402	polyubiquitin-B, partial [Antricola delacruzi]	17432											
	**39.**	183	gi|26353460	unnamed protein product [Mus musculus]	17214											
	**40.**	183	gi|195587566	GD13313 [Drosophila simulans]	21780											
	**41.**	183	gi|54610299	polyubiquitin-like protein [Schistosoma japonicum]	17621											
	**42.**	183	gi|226473064	polyubiquitin [Schistosoma japonicum]	21907											
	**43.**	182	gi|90076064	unnamed protein product [Macaca fascicularis]	23571											
	**44.**	182	gi|90075190	unnamed protein product [Macaca fascicularis]	17892											
	**45.**	182	gi|56199554	ubiquitin [Culicoides sonorensis]	21565											
	**46.**	180	gi|114152964	ubiquitin/40S ribosomal protein S27a fusion protein [Argas mon	26778											
	**47.**	180	gi|301617753	PREDICTED: ubiquitin-like [Xenopus (Silurana) tropicalis]	17410											
	**48.**	180	gi|339232974	ubiquitin family protein [Trichinella spiralis]	23593											
	**49.**	179	gi|197129049	putative ubiquitin C variant 1 [Taeniopygia guttata]	23410											
	**50.**	179	gi|348537407	PREDICTED: polyubiquitin-C-like [Oreochromis niloticus]	26763											
**3**	**1.**	70	gi|30983942	**putative polyubiquitin [Gossypium barbadense]**	15022	2.4	6	1039.4984	1038.4911	1038.5094	-17.61	91	99	0	---	K.EGIPPDQQR.L
								1039.4984	1038.4911	1038.5094	-17.60	15	23	0	9	K.EGIPPDQQR.L
								1067.6090	1066.6017	1066.6135	-11.02	121	129	0	---	K.ESTLHLVLR.L
								1067.6090	1066.6017	1066.6135	-11.01	45	53	0	20	K.ESTLHLVLR.L
								1523.7607	1522.7534	1522.7740	-13.48	87	99	1	---	K.IQDKEGIPPDQQR.L
								1523.7607	1522.7535	1522.7740	-13.46	11	23	1	---	K.IQDKEGIPPDQQR.L
**4**	**1.**	199	gi|51860756	**fatty acid-biding protein [Tyrophagus putrescentiae]**	14565	2.9e-013	10	714.4193	713.4120	713.4436	-44.22	92	97	0	---	K.LVQVQK.G
								984.5720	983.5647	983.5764	-11.86	98	106	0	---	K.GDKPVTIVR.E
								1123.4772	1122.4699	1122.4829	-11.57	66	74	0	---	K.LGEEFEEDR.A
								1398.6271	1397.6198	1397.6463	-18.93	64	74	1	---	K.FKLGEEFEEDR.A
								1494.6350	1493.6277	1493.6634	-23.88	66	78	1	---	K.LGEEFEEDRADGK.K
								1880.9003	1879.8930	1879.9415	-25.80	35	52	0	77	K.SASPTVEVIVDGDSYTIK.T
								1880.9003	1879.8930	1879.9415	-25.78	35	52	0	---	K.SASPTVEVIVDGDSYTIK.T
								2096.0220	2095.0147	2095.0433	-13.68	107	126	0	75	R.EFSEEGLTVTATVNGVTSVR.F
								2096.0220	2095.0147	2095.0433	-13.66	107	126	0	---	R.EFSEEGLTVTATVNGVTSVR.F
								2097.0071	2095.9998	2096.0274	-13.14	107	126	0	---	R.EFSEEGLTVTATVNGVTSVR.F + Deamidated (NQ)
	**2.**	113	gi|156938917	**allergen Tyr p 13 [Tyrophagus putrescentiae]**	14537	0.00012	7	714.4193	713.4120	713.4436	-44.22	92	97	0	---	K.LVQVQK.G
								984.5720	983.5647	983.5764	-11.86	98	106	0	---	K.GDKPVTIVR.E
								1123.4772	1122.4699	1122.4829	-11.57	66	74	0	---	K.LGEEFEEDR.A
								1398.6271	1397.6198	1397.6463	-18.93	64	74	1	---	K.FKLGEEFEEDR.A
								1494.6350	1493.6277	1493.6634	-23.88	66	78	1	---	K.LGEEFEEDRADGK.K
								1880.9003	1879.8930	1879.9415	-25.80	35	52	0	77	K.SASPTVEVIVDGDSYTIK.T
								1880.9003	1879.8930	1879.9415	-25.78	35	52	0	---	K.SASPTVEVIVDGDSYTIK.T
	**3.**	71	gi|419706568	Hypothetical protein PS4_64959 [Streptococcus salivarius PS4]	22289											
**5**	**1.**	210	gi|374073976	**Chain B, Structure Of Usp21 In Complex With Linear Diubi**	17235	2.3e-014	11	717.3257	716.3184	716.3453	-37.53	125	130	0	---	K.QLEDGR.T
								1039.4967	1038.4894	1038.5094	-19.25	34	42	0	21	K.EGIPPDQQR.L
								1039.4967	1038.4894	1038.5094	-19.25	110	118	0	---	K.EGIPPDQQR.L
								1067.5981	1066.5908	1066.6135	-21.24	140	148	0	---	K.ESTLHLVLR.L
								1067.5981	1066.5909	1066.6135	-21.20	64	72	0	33	K.ESTLHLVLR.L
								1081.5261	1080.5188	1080.5451	-24.34	131	139	0	---	R.TLSDYNIQK.E

								1460.7751	1459.7678	1459.7857	-12.26	75	87	1	---	R.GHMQIFVKTLTGK.T + Deamidated (NQ)
								1523.7476	1522.7403	1522.7740	-22.11	30	42	1	45	K.IQDKEGIPPDQQR.L
								1523.7476	1522.7403	1522.7740	-22.09	106	118	1	---	K.IQDKEGIPPDQQR.L
								1787.8772	1786.8699	1786.9200	-28.03	12	27	0	40	K.TITLEVEPSDTIENVK.A
								1787.8772	1786.8699	1786.9200	-28.03	88	103	0	---	K.TITLEVEPSDTIENVK.A
	**2.**	209	gi|1805696	polyubiquitin [Ceratitis capitata]	15038											
	**3.**	207	gi|114152964	ubiquitin/40S ribosomal protein S27a fusion protein [Argas mon	26778											
	**4.**	206	gi|340062	pro-ubiquitin, partial [Homo sapiens]	17776											
	**5.**	202	gi|228311825	Chain A, Crystal Structure Of Linear Di-Ubiquitin	17081											
	**6.**	200	gi|355753795	Polyubiquitin-C [Macaca fascicularis]	17261											
	**7.**	200	gi|28189426	similar to ubiquitin-S27a fusion protein [Bos taurus]	16717											
	**8.**	199	gi|54300702	ubiquitin C splice variant [Homo sapiens]	17142											
	**9.**	199	gi|403221030	ubiquitin [Theileria orientalis strain Shintoku]	17464											
	**10.**	199	gi|372467011	Chain A, Crystal Structure Of A Linear-Specific Ubiquitin Fab F	19263											
	**11.**	199	gi|225733991	Chain A, Nemo Cozi Domain Incomplex With Diubiquitin In P2	17245											
	**12.**	199	gi|7799051	ubiquitin [Cyanidium caldarium]	17293											
	**13.**	199	gi|395836339	PREDICTED: polyubiquitin-B [Otolemur garnettii]	17261											
	**14.**	199	gi|326931517	PREDICTED: polyubiquitin-B-like isoform 1 [Meleagris gallopa	17264											
	**15.**	199	gi|256079432	ubiquitin (ribosomal protein L40) [Schistosoma mansoni]	17214											
	**16.**	199	gi|226484009	ubiquitin C [Schistosoma japonicum]	17214											
	**17.**	199	gi|361130305	Chain A, Crystal Structure Of The Mouse Hoil1-L-Nzf In Comp	17101											
	**18.**	199	gi|195587566	GD13313 [Drosophila simulans]	21780											
	**19.**	199	gi|163575	polyubiquitin, partial [Bos taurus]	18477											
	**20.**	198	gi|71033201	ubiquitin [Theileria parva strain Muguga]	17479											
	**21.**	198	gi|600539	ubiquitin, partial [synthetic construct]	18459											
	**22.**	198	gi|172051100	ubiquitin/40S ribosomal protein S27a [Ornithodoros coriaceus]	17391											
	**23.**	198	gi|4506713	ubiquitin-40S ribosomal protein S27a precursor [Homo sapiens]	18296											
	**24.**	198	gi|296199681	PREDICTED: ubiquitin-40S ribosomal protein S27a-like [Callitl	18324											
	**25.**	198	gi|209875527	ubiquitin family protein [Cryptosporidium muris RN66]	17462											
	**26.**	198	gi|350537349	putative ubiquitin C variant 4 [Taeniopygia guttata]	17215											
	**27.**	198	gi|84998672	ubiquitin [Theileria annulata]	17935											
	**28.**	198	gi|426242585	PREDICTED: ubiquitin-40S ribosomal protein S27a-like [Ovis a	18244											
	**29.**	198	gi|442761305	Putative ubiquitin/40s ribosomal protein s27a fusion, partial [Ixo	17708											
	**30.**	198	gi|403277587	PREDICTED: ubiquitin-40S ribosomal protein S27a-like [Saimi	18300											
	**31.**	198	gi|296216667	PREDICTED: ubiquitin-40S ribosomal protein S27a-like [Callitl	18171											
	**32.**	197	gi|226477466	polyubiquitin [Schistosoma japonicum]	19260											
	**33.**	197	gi|28189839	similar to polyubiquitin [Bos taurus]	17644											
	**34.**	197	gi|212851	ubiquitin polyprotein (heat shock related), partial [Gallus gallus]	17648											
	**35.**	197	gi|428672795	ubiquitin family member protein [Babesia equi]	17555											
	**36.**	197	gi|354483686	PREDICTED: ubiquitin-40S ribosomal protein S27a-like [Cricet	18292											
	**37.**	196	gi|417408300	Putative ribosomal protein s27a, partial [Desmodus rotundus]	19210											
	**38.**	196	gi|395508039	PREDICTED: ubiquitin-40S ribosomal protein S27a [Sarcophilu	25788											
	**39.**	195	gi|223646258	Ubiquitin [Salmo salar]	21483											
	**40.**	195	gi|297697495	PREDICTED: ubiquitin-40S ribosomal protein S27a-like [Pongo	18111											
	**41.**	195	gi|440894648	Polyubiquitin-C, partial [Bos grunniens mutus]	11392											
	**42.**	195	gi|3954791	unnamed protein product [Gallus gallus]	17738											
	**43.**	195	gi|56199552	ubiquitin [Culicoides sonorensis]	18776											
	**44.**	194	gi|78099807	RecName: Full=Ubiquitin	7199											
	**45.**	194	gi|302393789	RecName: Full=Polyubiquitin; Contains: RecName: Full=Ubiqui	18789											
	**46.**	193	gi|343960965	ubiquitin [Pan troglodytes]	19800											
	**47.**	193	gi|440891703	Polyubiquitin-B [Bos grunniens mutus]	8819											
	**48.**	193	gi|1321735	ubiquitin [Carabus alpestris]	15669											
	**49.**	192	gi|119624911	ubiquitin B, isoform CRA_e [Homo sapiens]	16906											
	**50.**	192	gi|149063235	rCG21224 [Rattus norvegicus]	18359											
6	**1.**	110	gi|1402737	**major cold-shock protein, partial [Bacillus cereus]**	5004	0.00023	4	1964.8878	1963.8805	1963.9123	-16.18	29	45	0	---	K.TLEEGQEVTFEVEQGNR.-
								1964.8878	1963.8805	1963.9123	-16.17	29	45	0	19	K.TLEEGQEVTFEVEQGNR.-
								2802.2642	2801.2569	2801.3337	-27.44	3	28	0	70	K.GFGFIEVEGGEDVFVHFSAIQGEGFK.T

								2802.2642	2801.2569	2801.3337	-27.42	3	28	0	---	K.GFGFIEVEGGEDVFVHFSAIQGEGFK.T
	**2.**	107	gi|62421414	cold shock protein [Bacillus thuringiensis serovar kurstaki]	6625											
	**3.**	107	gi|63148792	cold shock protein [Bacillus thuringiensis serovar tolworthi]	6643											
	**4.**	105	gi|152975949	cold-shock DNA-binding domain-containing protein [Bacillus cyt	7331											
	**5.**	105	gi|228992244	Cold shock protein cspB [Bacillus pseudomycoides DSM 12442]	7347											
	**6.**	104	gi|30021641	cold shock protein [Bacillus cereus ATCC 14579]	7362											
	**7.**	103	gi|254752895	cold shock protein CspB [Bacillus anthracis str. Vollum]	7326											
**7**	**1.**	161	gi|30021641	**cold shock protein [Bacillus cereus ATCC 14579]**	7362	1.8e-009	7	810.3424	809.3351	809.3708	-44.07	8	13	0	---	K.WFNSEK.G
								810.3424	809.3351	809.3708	-44.05	8	13	0	---	K.WFNSEK.G
								1964.8627	1963.8554	1963.9123	-28.98	40	56	0	79	K.TLEEGQEVTFEVEQGNR.G
								1964.8627	1963.8554	1963.9123	-28.96	40	56	0	---	K.TLEEGQEVTFEVEQGNR.G
								2802.2454	2801.2381	2801.3337	-34.15	14	39	0	50	K.GFGFIEVEGGEDVFVHFSAIQGEGFK.T
								2802.2454	2801.2381	2801.3337	-34.13	14	39	0	---	K.GFGFIEVEGGEDVFVHFSAIQGEGFK.T
								2874.3025	2873.2952	2873.3791	-29.20	40	65	1	---	K.TLEEGQEVTFEVEQGNRGPQATNVNK.K
	**2.**	160	gi|254752895	cold shock protein CspB [Bacillus anthracis str. Vollum]	7326											
	**3.**	154	gi|63148792	cold shock protein [Bacillus thuringiensis serovar tolworthi]	6643											
	**4.**	152	gi|228992244	Cold shock protein cspB [Bacillus pseudomycoides DSM 12442]	7347											
	**5.**	146	gi|1402737	major cold-shock protein, partial [Bacillus cereus]	5004											
	**6.**	144	gi|62421414	cold shock protein [Bacillus thuringiensis serovar kurstaki]	6625											
	**7.**	142	gi|152975949	cold-shock DNA-binding domain-containing protein [Bacillus cyt	7331											
	**8.**	101	gi|229031192	Cold shock protein cspB [Bacillus cereus AH1271]	7348											
	**9.**	93	gi|229086124	Cold shock protein cspB [Bacillus cereus Rock3-44]	7333											
**8**	**1.**	158	gi|30022927	**cold shock protein [Bacillus cereus ATCC 14579]**	7234	3.7e-009	5	810.3574	809.3501	809.3708	-25.53	8	13	0	---	K.WFNSEK.G
								1935.8757	1934.8684	1934.9221	-27.76	40	56	0	---	K.TLEEGQEVSFEIVEGNR.G
								1935.8757	1934.8685	1934.9221	-27.74	40	56	0	96	K.TLEEGQEVSFEIVEGNR.G
								2774.2029	2773.1956	2773.3025	-38.53	14	39	0	37	K.GFGFIEVEGGDDVFVHFSAIQGDGFK.T
								2774.2029	2773.1956	2773.3025	-38.52	14	39	0	---	K.GFGFIEVEGGDDVFVHFSAIQGDGFK.T
	**2.**	147	gi|228993599	Cold shock protein cspB [Bacillus pseudomycoides DSM 12442]	7204											
	**3.**	110	gi|152977181	cold-shock DNA-binding domain-containing protein [Bacillus cyt	7279											
	**4.**	110	gi|403237935	cold shock protein [Bacillus sp. 10403023]	7274											
	**5.**	102	gi|218900031	cold-shock protein CspD [Bacillus cereus G9842]	7219											
	**6.**	102	gi|229032524	Cold shock protein cspB [Bacillus cereus AH1271]	7233											
	**7.**	102	gi|239826758	cold-shock DNA-binding domain-containing protein [Geobacillus	7310											
	**8.**	102	gi|423394881	cold shock-like protein CspD [Bacillus cereus BAG2X1-1]	7219											
	**9.**	102	gi|16974809	Chain A, Bacillus Caldolyticus Cold-Shock Protein Mutants To S	7302											
	**10.**	102	gi|16974805	Chain A, Bacillus Caldolyticus Cold-Shock Protein Mutants To S	7345											
	**11.**	102	gi|56419891	cold shock protein [Geobacillus kaustophilus HTA426]	7329											
	**12.**	102	gi|293375824	major cold shock protein CspA [Turicibacter sanguinis PC909]	7091											
	**13.**	102	gi|16974803	Chain A, Bacillus Caldolyticus Cold-Shock Protein Mutants To S	7418											
**9**	**1.**	150	gi|66841026	**alpha-amylase inhibitor 0.19 [Triticum aestivum]**	13340	2.3e-008	9	1570.7417	1569.7344	1569.7933	-37.52	21	34	0	24	R.LQCNGSQVPEAVLR.D
								1570.7417	1569.7344	1569.7933	-37.52	21	34	0	---	R.LQCNGSQVPEAVLR.D
								1612.6902	1611.6829	1611.7390	-34.79	62	77	0	70	K.EHGAQEGQAGTGAFPR.C
								1612.6902	1611.6829	1611.7390	-34.78	62	77	0	---	K.EHGAQEGQAGTGAFPR.C
								1663.7909	1662.7836	1662.8287	-27.12	96	111	0	---	R.LPIVVDASGDGAYVCK.D
								1862.7047	1861.6974	1861.7658	-36.73	35	48	0	---	R.DCCQQLAHISEWCR.C
								1862.7047	1861.6974	1861.7658	-36.73	35	48	0	19	R.DCCQQLAHISEWCR.C
								1863.7147	1862.7074	1862.7498	-22.76	35	48	0	---	R.DCCQQLAHISEWCR.C + Deamidated (NQ)
								2807.2292	2806.2219	2806.4357	-76.19	85	111	1	---	K.LTAASITAVCRLPIVVDASGDGAYVCK.D
	**2.**	150	gi|123963	RecName: Full=Alpha-amylase inhibitor 0.19; AltName: Full=0.	13899											
	**3.**	149	gi|108597921	dimeric alpha-amylase inhibitor precursor, partial [Triticum aesti	14030											

	**4.**	149	gi|386877048	dimeric alpha-amylase inhibitor, partial [Aegilops kotschyi]	14199											
	**5.**	149	gi|386877062	dimeric alpha-amylase inhibitor, partial [Aegilops geniculata]	14543											
	**6.**	149	gi|386877046	dimeric alpha-amylase inhibitor, partial [Aegilops tauschii]	14670											
	**7.**	148	gi|386877044	dimeric alpha-amylase inhibitor, partial [Aegilops tauschii]	14805											
	**8.**	148	gi|386877050	dimeric alpha-amylase inhibitor, partial [Aegilops tauschii]	14826											
	**9.**	148	gi|227809300	dimeric alpha-amylase inhibitor [Aegilops uniaristata]	15758											
	**10.**	148	gi|227809384	dimeric alpha-amylase inhibitor [Aegilops tauschii]	15791											
	**11.**	148	gi|386877060	dimeric alpha-amylase inhibitor, partial [Aegilops longissima]	14954											
	**12.**	147	gi|65993781	dimeric alpha-amylase inhibitor [Triticum aestivum]	15688											
	**13.**	147	gi|227809005	dimeric alpha-amylase inhibitor [Triticum dicoccoides]	15730											
	**14.**	147	gi|227809314	dimeric alpha-amylase inhibitor [Aegilops tauschii]	15762											
	**15.**	147	gi|227809364	dimeric alpha-amylase inhibitor [Aegilops tauschii]	15742											
	**16.**	147	gi|386877038	dimeric alpha-amylase inhibitor [Triticum aestivum]	15702											
	**17.**	147	gi|54778521	0.19 dimeric alpha-amylase inhibitor [Aegilops tauschii]	13881											
	**18.**	130	gi|386877068	dimeric alpha-amylase inhibitor, partial [Triticum aestivum]	14416											
	**19.**	129	gi|255988225	dimeric alpha-amylase inhibitor [Triticum aestivum]	15666											
	**20.**	127	gi|54778503	0.19 dimeric alpha-amylase inhibitor [Triticum aestivum]	13827											
	**21.**	125	gi|65993925	dimeric alpha-amylase inhibitor [Triticum aestivum]	15702											
	**22.**	125	gi|227809156	dimeric alpha-amylase inhibitor [Triticum dicoccoides]	15716											
	**23.**	125	gi|227809366	dimeric alpha-amylase inhibitor [Aegilops tauschii]	15714											
	**24.**	120	gi|114215934	dimeric alpha-amylase inhibitor [Aegilops sharonensis]	13962											
	**25.**	120	gi|114215808	dimeric alpha-amylase inhibitor [Triticum dicoccoides]	13922											
	**26.**	120	gi|114215932	dimeric alpha-amylase inhibitor [Aegilops sharonensis]	13891											
	**27.**	120	gi|114215804	dimeric alpha-amylase inhibitor [Triticum dicoccoides]	13863											
	**28.**	120	gi|114215806	dimeric alpha-amylase inhibitor [Triticum dicoccoides]	13863											
	**29.**	120	gi|386877056	dimeric alpha-amylase inhibitor, partial [Aegilops longissima]	14792											
	**30.**	120	gi|386877058	dimeric alpha-amylase inhibitor, partial [Aegilops longissima]	14719											
	**31.**	119	gi|54778515	0.19 dimeric alpha-amylase inhibitor [Aegilops tauschii]	13869											
	**32.**	119	gi|227809078	dimeric alpha-amylase inhibitor [Triticum dicoccoides]	15722											
	**33.**	119	gi|227809102	dimeric alpha-amylase inhibitor [Triticum dicoccoides]	15678											
	**34.**	119	gi|227809250	dimeric alpha-amylase inhibitor [Triticum dicoccoides]	15694											
	**35.**	119	gi|227809252	dimeric alpha-amylase inhibitor [Triticum dicoccoides]	15716											
	**36.**	119	gi|227809254	dimeric alpha-amylase inhibitor [Triticum dicoccoides]	15753											
	**37.**	119	gi|227809431	dimeric alpha-amylase inhibitor [Hordeum vulgare]	15605											
	**38.**	117	gi|65993829	dimeric alpha-amylase inhibitor [Triticum aestivum]	15722											
	**39.**	117	gi|227809316	dimeric alpha-amylase inhibitor [Aegilops tauschii]	15704											
	**40.**	117	gi|227809370	dimeric alpha-amylase inhibitor [Aegilops tauschii]	15756											
	**41.**	111	gi|114215916	dimeric alpha-amylase inhibitor [Aegilops sharonensis]	13775											
	**42.**	111	gi|114215786	dimeric alpha-amylase inhibitor [Triticum dicoccoides]	13833											
	**43.**	111	gi|114215842	dimeric alpha-amylase inhibitor [Aegilops speltoides]	13829											
	**44.**	111	gi|114216000	dimeric alpha-amylase inhibitor [Aegilops longissima]	13831											
	**45.**	111	gi|114215908	dimeric alpha-amylase inhibitor [Aegilops sharonensis]	13784											
	**46.**	110	gi|227809096	dimeric alpha-amylase inhibitor [Triticum dicoccoides]	15664											
	**47.**	104	gi|114215896	dimeric alpha-amylase inhibitor [Aegilops sharonensis]	13787											
	**48.**	103	gi|227809117	dimeric alpha-amylase inhibitor [Triticum dicoccoides]	15904											
	**49.**	103	gi|227809136	dimeric alpha-amylase inhibitor [Triticum dicoccoides]	15902											
	**50.**	102	gi|386877040	dimeric alpha-amylase inhibitor, partial [Triticum aestivum]	15014											
**10a**	**1.**	108	gi|269999913	**ferritin 3 [Coffea arabica]**	15114	0.00037	8	865.3251	864.3178	864.3461	-32.68	2	8	0	---	K.ESSEEER.E
								1066.5249	1065.5176	1065.5641	-43.58	14	21	1	---	K.LMKYQNIR.G + Deamidated (NQ)
								1094.5753	1093.5680	1093.5768	-7.99	97	105	0	---	K.ISDYVTQLR.M
								1094.5753	1093.5680	1093.5768	-7.98	97	105	0	52	K.ISDYVTQLR.M
								1222.6641	1221.6568	1221.6717	-12.23	96	105	1	30	K.KISDYVTQLR.M
								1222.6641	1221.6568	1221.6717	-12.19	96	105	1	---	K.KISDYVTQLR.M
								1459.6038	1458.5965	1458.6222	-17.62	2	13	1	---	K.ESSEEEREHAEK.L
								1625.8469	1624.8396	1624.7933	28.5	106	119	1	---	R.MVGKGHGVWHFNQK.L + Deamidated (NQ)
	**2.**	103	gi|269999915	ferritin [Coffea arabica]	21268											
	**3.**	92	gi|290020584	ferritin [Coffea arabica]	32304											

**10b**	**1.**	141	gi|28630234	**ferritin heavy chain polypeptide 1 [Branchiostoma lanceolatu**	19901	2.8e-007	6	1284.5800	1283.5727	1283.6398	-52.26	155	166	0	117	R.VGSGLGEYIFDK.E
								1284.5800	1283.5727	1283.6398	-52.23	155	166	0	---	R.VGSGLGEYIFDK.E
								1440.6661	1439.6588	1439.7409	-56.99	154	166	1	---	K.RVGSGLGEYIFDK.E
								1440.6661	1439.6589	1439.7409	-56.96	154	166	1	13	K.RVGSGLGEYIFDK.E
								2586.1152	2585.1079	2585.3489	-93.22	144	166	2	---	K.QIGEYVTNLKRVGSGLGEYIFDK.E
								2586.1152	2585.1080	2585.3489	-93.21	144	166	2	---	K.QIGEYVTNLKRVGSGLGEYIFDK.E
	**2.**	127	gi|256079612	**ferritin [Schistosoma mansoni]**	21035	7.00E-06	7	856.4756	855.4683	855.4198	56.7	64	70	1	---	R.KASHEER.E
								856.4756	855.4683	855.4198	56.7	64	70	1	---	R.KASHEER.E
								1284.5800	1283.5727	1283.6398	-52.26	165	176	0	117	R.VGSGLGQYLFDK.M + Deamidated (NQ)
								1284.5800	1283.5727	1283.6398	-52.23	165	176	0	---	R.VGSGLGQYLFDK.M + Deamidated (NQ)
								1445.5488	1444.5415	1444.6946	-105.95	71	82	1	---	R.EHAENLAKYQNK.R + Deamidated (NQ)
								2691.1255	2690.1182	2690.2347	-43.31	131	153	0	---	K.NNDPALTDFIESQYLHEQEDAIK.Q + Deamidated (NQ)
								2691.1255	2690.1182	2690.2347	-43.31	131	153	0	---	K.NNDPALTDFIESQYLHEQEDAIK.Q + Deamidated (NQ
	**3.**	127	gi|256079614	ferritin [Schistosoma mansoni]	21064											
	**4.**	96	gi|290020584	ferritin [Coffea arabica]	32304	0.0099	7	1011.5844	1010.5771	1010.5219	54.7	166	173	1	---	R.EHPEKLMK.Y
								1094.5277	1093.5204	1093.5768	-51.52	254	262	0	---	K.ISDYVTQLR.M
								1094.5277	1093.5204	1093.5768	-51.51	254	262	0	49	K.ISDYVTQLR.M
								1222.6162	1221.6089	1221.6717	-51.40	253	262	1	---	K.KISDYVTQLR.M
								1222.6162	1221.6089	1221.6717	-51.40	253	262	1	35	K.KISDYVTQLR.M
								1625.7935	1624.7862	1624.7933	-4.36	263	276	1	---	R.MVGKGHGVWHFNQK.L + Deamidated (NQ)
								1625.7935	1624.7862	1624.7933	-4.34	263	276	1	---	R.MVGKGHGVWHFNQK.L + Deamidated (NQ)
	**5.**	94	gi|475511929	hypothetical protein F775_14174 [Aegilops tauschii]	11510											
	**6.**	90	gi|384371285	ferritin [Cerebratulus lacteus]	19645											
	**7.**	87	gi|494746081	nicotinamide-nucleotide adenylyltransferase [Acinetobacter]	22001											
**10c**	**1.**	119	gi|28630234	**ferritin heavy chain polypeptide 1 [Branchiostoma lanceolatu**	19901	4.4e-005	4	1284.6028	1283.5955	1283.6398	-34.49	155	166	0	110	R.VGSGLGEYIFDK.E
								1284.6028	1283.5955	1283.6398	-34.47	155	166	0	---	R.VGSGLGEYIFDK.E
								1440.6979	1439.6906	1439.7409	-34.92	154	166	1	2	K.RVGSGLGEYIFDK.E
								1440.6979	1439.6906	1439.7409	-34.90	154	166	1	---	K.RVGSGLGEYIFDK.E
	**2.**	118	gi|256079612	ferritin [Schistosoma mansoni]	21035											
	**3.**	118	gi|256079614	ferritin [Schistosoma mansoni]	21064											
**11**	**1.**	122	gi|156124994	**Tyr p 3 allergen [Tyrophagus putrescentiae]**	20059	1.5e-005	5	1465.6981	1464.6908	1464.7573	-45.36	94	107	0	---	R.YNTLTQGSGGQVIK.S
								1866.9475	1865.9402	1866.0211	-43.33	170	188	0	---	K.EGGSSPSALQIVTVPIVGR.D
								1866.9475	1865.9402	1866.0211	-43.33	170	188	0	70	K.EGGSSPSALQIVTVPIVGR.D
								2274.1313	2273.1240	2273.2168	-40.82	40	62	0	---	R.IVGGVAATPGQAPYQVSLLYGGR.H
								2274.1314	2273.1241	2273.2168	-40.80	40	62	0	43	R.IVGGVAATPGQAPYQVSLLYGGR.H
**12**	**1.**	237	gi|75762754	**Nucleoside diphosphate kinase [Bacillus thuringiensis serovar**	19962	4.6e-017	14	804.2491	803.2418	803.3054	-79.19	1	6	0	---	-.MHNCDK.G
								819.4338	818.4265	818.4650	-47.06	56	63	0	---	K.GFQLVGAK.L
								867.4597	866.4524	866.4902	-43.57	153	159	0	---	R.EIAIFFK.E
								947.5119	946.5046	946.5600	-58.50	55	63	1	---	K.KGFQLVGAK.L
								975.5278	974.5205	974.5549	-35.27	43	51	0	---	R.AFIGEIVAR.F
								975.5278	974.5206	974.5549	-35.24	43	51	0	44	R.AFIGEIVAR.F
								1305.6566	1304.6493	1304.6949	-34.94	118	129	0	---	K.TRPHEAAPGTIR.G
								1305.6566	1304.6493	1304.6949	-34.93	118	129	0	39	K.TRPHEAAPGTIR.G
								1406.6918	1405.6845	1405.7388	-38.62	31	42	0	22	K.TFLMVKPDGVQR.A + Oxidation (M)
								1406.6918	1405.6845	1405.7388	-38.60	31	42	0	---	K.TFLMVKPDGVQR.A + Oxidation (M)
								1527.6879	1526.6806	1526.7325	-33.98	139	152	0	68	K.NIIHGSDSLESAER.E
								1527.6879	1526.6806	1526.7325	-33.96	139	152	0	---	K.NIIHGSDSLESAER.E
								1959.9127	1958.9054	1958.9877	-41.98	153	168	1	---	R.EIAIFFKEEELVDYSK.L

								1967.8875	1966.8802	1966.9571	-39.07	64	80	0	---	K.LMQVTPEIAGQHYAEHK.E + Oxidation (M)
	**2.**	233	gi|228920325	Nucleoside diphosphate kinase [Bacillus thuringiensis serovar hu	16645											
	**3.**	233	gi|30019663	nucleoside diphosphate kinase [Bacillus cereus ATCC 14579]	16631											
	**4.**	223	gi|47565977	nucleoside diphosphate kinase [Bacillus cereus G9241]	16604											
	**5.**	223	gi|229010922	Nucleoside diphosphate kinase [Bacillus mycoides DSM 2048]	16716											
	**6.**	223	gi|229160565	Nucleoside diphosphate kinase [Bacillus cereus R309803]	16646											
	**7.**	223	gi|229096106	Nucleoside diphosphate kinase [Bacillus cereus Rock3-29]	16617											
	**8.**	223	gi|423397671	nucleoside diphosphate kinase [Bacillus cereus BAG2X1-1]	16645											
	**9.**	220	gi|229102218	Nucleoside diphosphate kinase [Bacillus cereus Rock3-28]	18617											
	**10.**	220	gi|163939430	nucleoside diphosphate kinase [Bacillus weihenstephanensis KBA	18975											
	**11.**	220	gi|42736636	nucleoside diphosphate kinase, putative [Bacillus cereus ATCC 1	18863											
	**12.**	211	gi|118477068	nucleoside diphosphate kinase [Bacillus thuringiensis str. Al Hak	19905											
	**13.**	206	gi|30261610	nucleoside diphosphate kinase [Bacillus anthracis str. Ames]	16590											
	**14.**	206	gi|167633279	putative nucleoside diphosphate kinase [Bacillus anthracis str. A(	16518											
	**15.**	206	gi|224983364	Chain A, Crystal Structure Of Pndk From Bacillus Anthracis	16618											
	**16.**	206	gi|229084618	Nucleoside diphosphate kinase [Bacillus cereus Rock3-44]	16645											
	**17.**	206	gi|228945214	Nucleoside diphosphate kinase [Bacillus thuringiensis serovar mo	16590											
	**18.**	205	gi|229183809	Nucleoside diphosphate kinase [Bacillus cereus BGSC 6E1]	17301											
	**19.**	203	gi|301053152	nucleoside diphosphate kinase [Bacillus cereus biovar anthracis s	18849											
	**20.**	203	gi|229115062	Nucleoside diphosphate kinase [Bacillus cereus Rock1-3]	15868											
	**21.**	178	gi|228907247	Nucleoside diphosphate kinase [Bacillus thuringiensis IBL 200]	16645											
	**22.**	175	gi|229058250	Nucleoside diphosphate kinase [Bacillus cereus AH603]	16851											
	**23.**	175	gi|228984693	Nucleoside diphosphate kinase [Bacillus thuringiensis serovar toc	16634											
	**24.**	175	gi|423610043	nucleoside diphosphate kinase [Bacillus cereus VD107]	16606											
	**25.**	159	gi|228990629	Nucleoside diphosphate kinase [Bacillus pseudomycoides DSM 1	16605											
	**26.**	137	gi|152975038	nucleoside diphosphate kinase [Bacillus cytotoxicus NVH 391-98	16715											
	**27.**	105	gi|308174065	nucleoside diphosphate kinase [Bacillus amyloliquefaciens DSM	16843											
	**28.**	105	gi|375362787	nucleoside diphosphate kinase [Bacillus amyloliquefaciens subsp.	16830											
	**29.**	105	gi|384265870	nucleoside diphosphate kinase [Bacillus amyloliquefaciens subsp.	16852											
	**30.**	101	gi|154686521	nucleoside diphosphate kinase [Bacillus amyloliquefaciens FZB4	16839											
	**31.**	100	gi|386758853	nucleoside diphosphate kinase [Bacillus sp. JS]	18737											
	**32.**	99	gi|350266446	nucleoside diphosphate kinase [Bacillus subtilis subsp. spizizenii	16786											
	**33.**	99	gi|398306796	mulitfunctional nucleoside diphosphate kinase/apyrimidinic endo	16833											
	**34.**	99	gi|221310189	nucleoside diphosphate kinase [Bacillus subtilis subsp. subtilis st	16817											
	**35.**	98	gi|16079330	nucleoside diphosphate kinase [Bacillus subtilis subsp. subtilis st	16948											
	**36.**	97	gi|398311220	mulitfunctional nucleoside diphosphate kinase/apyrimidinic endo	16829											
	**37.**	97	gi|242253868	trypsinogen precursor [Sus scrofa]	26549											
	**38.**	94	gi|999627	Chain B, Refined 1.8 Angstroms Resolution Crystal Structure Of	8928											
	**39.**	93	gi|110590762	Chain A, Trypsin In Complex With Borate	24143											
	**40.**	93	gi|2914482	Chain A, Complex Of The Second Kunitz Domain Of Tissue Fac	24145											
	**41.**	93	gi|494360	Chain A, The Refined 1.6 Angstroms Resolution Crystal Structu	24143											
	**42.**	92	gi|3318722	Chain E, Leech-Derived Tryptase InhibitorTRYPSIN COMPLE	24142											
	**43.**	92	gi|136429	RecName: Full=Trypsin; Flags: Precursor	25078											
	**44.**	88	gi|1942351	Chain A, Crystal Structure Of The First Active Autolysate Form	13569											
	**45.**	87	gi|315583496	Chain A, The Bowman-Birk Type Inhibitor From Mung Bean In	24115											
	**46.**	86	gi|157878102	Chain E, Complex Of Eeti-Ii With Porcine Trypsin	24841											
	**47.**	86	gi|134298006	nucleoside diphosphate kinase [Desulfotomaculum reducens MI-1	16589											
**13**	**1.**	148	gi|423464038	**superoxide dismutase [Mn] 1 [Bacillus cereus BAG6O-1]**	22708	3.7e-008	16	1126.5400	1125.5327	1125.5237	8.03	23	31	0	---	K.ETMNIHHTK.H + Oxidation (M)
								1522.7200	1521.7127	1521.7140	-0.83	110	122	1	---	K.FGSFDAFKEEFAK.A
								1881.9729	1880.9656	1880.9843	-9.94	54	70	0	17	K.SVEELVANLNEVPEAIR.T
								1881.9729	1880.9656	1880.9843	-9.94	54	70	0	---	K.SVEELVANLNEVPEAIR.T
								2251.0718	2250.0645	2250.0858	-9.46	179	197	0	23	R.RPDYIGAFWNVVDWNAAEK.R
								2251.0718	2250.0645	2250.0858	-9.45	179	197	0	---	R.RPDYIGAFWNVVDWNAAEK.R
								2269.0918	2268.0845	2268.0851	-0.25	4	22	0	---	K.HELPNLPYAYDALEPHFDK.E
								2451.1958	2450.1885	2450.2019	-5.45	159	178	0	23	K.TPVIGLDVWEHAYYLNYQNR.R
								2451.1958	2450.1885	2450.2019	-5.45	159	178	0	---	K.TPVIGLDVWEHAYYLNYQNR.R
								2468.1978	2467.1905	2467.2172	-10.80	2	22	1	---	M.AKHELPNLPYAYDALEPHFDK.E

								3332.5891	3331.5818	3331.5997	-5.37	128	158	0	---	R.FGSGWAWLVVNNGELEVTSTPNQDSPLTEGK.T
								3332.5891	3331.5818	3331.5997	-5.37	128	158	0	1	R.FGSGWAWLVVNNGELEVTSTPNQDSPLTEGK.T
								3377.5476	3376.5403	3376.5822	-12.41	4	31	1	---	K.HELPNLPYAYDALEPHFDKETMNIHHTK.H + Deamidated (NQ); Oxidation (M
								3563.6882	3562.6809	3562.7229	-11.79	75	109	0	---	R.NNGGGHANHTFFWTILSPNGGGQPVGELATAIEAK.F + Deamidated (NQ)
								3564.6936	3563.6863	3563.7070	-5.79	75	109	0	---	R.NNGGGHANHTFFWTILSPNGGGQPVGELATAIEAK.F + 2 Deamidated (NQ)
								3598.7334	3597.7261	3597.6906	9.87	23	53	1	---	K.ETMNIHHTKHHNTYITNLNAALEDHAELADK.S + Deamidated (NQ); Oxidatic
	**2.**	147	gi|254721868	superoxide dismutase, Mn [Bacillus anthracis str. A1055]	22680											
	**3.**	136	gi|30264347	superoxide dismutase [Bacillus anthracis str. Ames]	22650											
	**4.**	136	gi|228902798	Superoxide dismutase [Bacillus thuringiensis IBL 4222]	22649											
	**5.**	136	gi|423612489	superoxide dismutase [Mn] 1 [Bacillus cereus VD107]	22607											
	**6.**	136	gi|52141224	superoxide dismutase [Bacillus cereus E33L]	22676											
	**7.**	134	gi|301055769	Mn containing superoxide dismutase [Bacillus cereus biovar anth	23522											
	**8.**	124	gi|73535546	Chain A, Crystal Structure Of Soda-1 (Ba4499) From Bacillus A	23704											
	**9.**	123	gi|306447497	manganese superoxide dismutase [Bacillus sp. MHS47]	22616											
	**10.**	118	gi|229180552	Superoxide dismutase [Bacillus cereus 172560W]	22636											
	**11.**	115	gi|229093344	Superoxide dismutase [Bacillus cereus Rock3-42]	22664											
	**12.**	114	gi|30022355	superoxide dismutase [Mn] [Bacillus cereus ATCC 14579]	24738											
	**13.**	108	gi|423385779	superoxide dismutase [Mn] 1 [Bacillus cereus BAG1X1-2]	22680											
	**14.**	106	gi|228941439	Superoxide dismutase [Bacillus thuringiensis serovar berliner AT	22616											
	**15.**	102	gi|229031924	Superoxide dismutase [Bacillus cereus AH1271]	22636											
	**16.**	102	gi|229163220	Superoxide dismutase [Bacillus cereus R309803]	22615											
	**17.**	102	gi|229019498	Superoxide dismutase [Bacillus cereus AH1273]	22635											
	**18.**	92	gi|229086844	Superoxide dismutase [Bacillus cereus Rock3-44]	22599											
	**19.**	92	gi|163942034	superoxide dismutase [Bacillus weihenstephanensis KBAB4]	22577											
	**20.**	92	gi|423660877	superoxide dismutase [Mn] 1 [Bacillus cereus VDM022]	22578											
	**21.**	90	gi|423417797	superoxide dismutase [Mn] 1 [Bacillus cereus BAG3X2-1]	22605											
	**22.**	90	gi|423470494	superoxide dismutase [Mn] 1 [Bacillus cereus BAG6O-2]	22577											
**14a**	**1.**	123	gi|15149252	**triosephosphate isomerase B [Xiphophorus maculatus]**	26761	1.2e-005	9	954.4706	953.4633	953.4760	-13.24	5	12	0	---	K.FFVGGNWK.M
								1082.5620	1081.5547	1081.5709	-14.96	4	12	1	---	R.KFFVGGNWK.M
								1232.6313	1231.6240	1231.6157	6.78	193	204	0	---	K.TNVSDAVANSVR.I
								1602.8716	1601.8643	1601.8817	-10.88	159	173	0	60	K.VVLAYEPVWAIGTGK.T
								1602.8716	1601.8643	1601.8817	-10.87	159	173	0	---	K.VVLAYEPVWAIGTGK.T
								1641.8429	1640.8356	1640.7729	38.2	84	97	0	---	K.DCGVNWVILGHSER.R
								1721.8578	1720.8505	1720.8856	-20.40	174	188	1	33	K.TASPQQAQEVHEKLR.A
								1721.8578	1720.8505	1720.8856	-20.39	174	188	1	---	K.TASPQQAQEVHEKLR.A
								2211.0898	2210.0825	2210.0678	6.67	32	51	0	---	K.VDSNVEVVCGAPSIYLDFAR.S
	**2.**	123	gi|47206738	unnamed protein product [Tetraodon nigroviridis]	26945											
	**3.**	116	gi|432908784	PREDICTED: triosephosphate isomerase B [Oryzias latipes]	26955											
	**4.**	116	gi|410905727	PREDICTED: triosephosphate isomerase B-like [Takifugu rubrip	26818											
	**5.**	111	gi|295792352	triosephosphate isomerase B [Epinephelus coioides]	26841											
	**6.**	110	gi|126211567	triose phosphate isomerase B [Poecilia reticulata]	26873											
	**7.**	106	gi|46849383	triose phosphate isomerase B [Oryzias latipes]	22945											
	**8.**	104	gi|15149246	triosephosphate isomerase [Acipenser brevirostrum]	27126											
	**9.**	104	gi|442565872	triose-phosphate isomerase [Dermatophagoides farinae]	27345											
	**10.**	98	gi|6624231	triose phosphate isomerase [Lethenteron reissneri]	22966											
	**11.**	97	gi|46849427	triose phosphate isomerase [Acipenser baerii]	22890											
	**12.**	94	gi|70997109	triosephosphate isomerase [Aspergillus fumigatus Af293]	28279											
**14b**	**1.**	90	gi|297529143	**ribonuclease HIII [Geobacillus sp. C56-T3]**	34420	0.033	17	856.4683	855.4610	855.4675	-7.60	69	76	2	---	K.RGADPGKR.Q
								856.4683	855.4610	855.4675	-7.55	69	76	2	---	K.RGADPGKR.Q
								930.4198	929.4125	929.4818	-74.51	135	142	0	9	K.QLNDEAIK.R
								930.4198	929.4125	929.4818	-74.51	135	142	0	---	K.QLNDEAIK.R
								1001.4940	1000.4867	1000.5917	-104.89	125	134	1	---	K.IAALGVKDSK.Q
								1001.4940	1000.4867	1000.5917	-104.87	125	134	1	26	K.IAALGVKDSK.Q
								1077.5049	1076.4976	1076.5172	-18.20	286	295	0	6	R.GAEVLETCAK.L

								1077.5049	1076.4976	1076.5172	-18.18	286	295	0	---	R.GAEVLETCAK.L
								1234.6006	1233.5933	1233.5924	0.72	167	176	1	---	R.WQRSGMPQTK.M + Oxidation (M)
								1320.5018	1319.4945	1319.6179	-93.52	57	68	1	---	K.AAEQEAAKWMGK.R + Deamidated (NQ)
								1475.6650	1474.6577	1474.7350	-52.42	57	69	2	---	K.AAEQEAAKWMGKR.G
								1493.6372	1492.6299	1492.7279	-65.62	167	178	2	---	R.WQRSGMPQTKMK.A + Oxidation (M)
								1511.6133	1510.6060	1510.6908	-56.14	167	178	2	---	R.WQRSGMPQTKMK.A + 2 Deamidated (NQ); 2 Oxidation (M)
								1511.6133	1510.6060	1510.6908	-56.12	167	178	2	---	R.WQRSGMPQTKMK.A + 2 Deamidated (NQ); 2 Oxidation (M)
								1568.6309	1567.6236	1567.8066	-116.75	76	88	2	1	K.RQERETASPPLER.R
								1568.6309	1567.6236	1567.8066	-116.72	77	89	2	---	R.QERETASPPLERR.L
								1993.8503	1992.8430	1992.9939	-75.68	1	17	0	---	-.MSNYVIQADQQLLDALR.A + Oxidation (M)
**15a**	**1.**	148	gi|30264347	**superoxide dismutase [Bacillus anthracis str. Ames]**	22650	5.6e-008	12	1522.6169	1521.6096	1521.7140	-68.58	110	122	1	---	K.FGSFDAFKEEFAK.A
								1522.6169	1521.6097	1521.7140	-68.56	110	122	1	61	K.FGSFDAFKEEFAK.A
								1881.8632	1880.8559	1880.9843	-68.28	54	70	0	43	K.SVEELVANLNEVPEAIR.T
								1881.8632	1880.8559	1880.9843	-68.26	54	70	0	---	K.SVEELVANLNEVPEAIR.T
								2250.9336	2249.9263	2250.0858	-70.87	179	197	0	6	R.RPDYIGAFWNVVDWNAAEK.R
								2250.9336	2249.9263	2250.0858	-70.87	179	197	0	---	R.RPDYIGAFWNVVDWNAAEK.R
								2432.0115	2431.0042	2431.1880	-75.60	32	53	0	---	K.HHNTYITNLNAALEGHAELADK.S
								2432.0115	2431.0042	2431.1880	-75.59	32	53	0	---	K.HHNTYITNLNAALEGHAELADK.S
								2451.0325	2450.0252	2450.2019	-72.11	159	178	0	6	K.TPVIGLDVWEHAYYLNYQNR.R
								2451.0325	2450.0252	2450.2019	-72.10	159	178	0	---	K.TPVIGLDVWEHAYYLNYQNR.R
								2468.0471	2467.0398	2467.2172	-71.88	2	22	1	---	M.AKHELPNLPYAYDALEPHFDK.E
								3333.3608	3332.3535	3332.5837	-69.08	128	158	0	---	R.FGSGWAWLVVNNGELEVTSTPNQDSPLTEGK.T + Deamidated (NQ)
	**2.**	148	gi|434377442	superoxide dismutase [Bacillus thuringiensis HD-789]	22649											
	**3.**	148	gi|306447497	manganese superoxide dismutase [Bacillus sp. MHS47]	22616											
	**4.**	148	gi|488102083	superoxide dismutase [Bacillus cereus]	22607											
	**5.**	147	gi|301055769	Mn containing superoxide dismutase [Bacillus cereus biovar anth	23522											
	**6.**	147	gi|30022355	superoxide dismutase [Mn] [Bacillus cereus ATCC 14579]	24738											
	**7.**	141	gi|446974768	superoxide dismutase [Bacillus cereus]	22664											
	**8.**	141	gi|384188348	superoxide dismutase (Mn) [Bacillus thuringiensis serovar chiner	22616											
	**9.**	141	gi|446974782	superoxide dismutase [Bacillus anthracis]	22680											
	**10.**	141	gi|446974769	superoxide dismutase [Bacillus cereus]	22708											
	**11.**	141	gi|52141224	superoxide dismutase [Bacillus cereus E33L]	22676											
	**12.**	140	gi|73535546	Chain A, Crystal Structure Of Soda-1 (Ba4499) From Bacillus A	23704											
	**13.**	135	gi|446974778	superoxide dismutase [Bacillus cereus]	22636											
	**14.**	135	gi|446974774	superoxide dismutase [Bacillus cereus]	22615											
	**15.**	134	gi|446974777	superoxide dismutase [Bacillus cereus]	22635											
	**16.**	134	gi|446974772	superoxide dismutase [Bacillus cereus]	22605											
	**17.**	134	gi|488040468	superoxide dismutase [Bacillus cereus]	22577											
	**18.**	122	gi|33465735	manganese dependent superoxide dismutase [Bacillus anthracis]	15211											
	**19.**	122	gi|33465743	manganese dependent superoxide dismutase [Bacillus thuringiens	15155											
	**20.**	122	gi|33465751	manganese dependent superoxide dismutase [Bacillus thuringiens	15269											
	**21.**	122	gi|33465747	manganese dependent superoxide dismutase [Bacillus thuringiens	15245											
	**22.**	122	gi|33465769	manganese dependent superoxide dismutase [Bacillus cereus]	15216											
	**23.**	121	gi|146262040	SOD [Bacillus subtilis]	15574											
	**24.**	116	gi|33465753	manganese dependent superoxide dismutase [Bacillus thuringiens	15239											
	**25.**	104	gi|507009559	superoxide dismutase [Mn] 1 [Bacillus cereus]	22623											
	**26.**	97	gi|446974779	superoxide dismutase [Bacillus cereus]	22636											
	**27.**	97	gi|446974780	superoxide dismutase [Bacillus cereus]	22680											
**15b**	**1.**	145	gi|30264347	**superoxide dismutase [Bacillus anthracis str. Ames]**	22650	1.1e-007	13	1522.6658	1521.6585	1521.7140	-36.47	110	122	1	37	K.FGSFDAFKEEFAK.A
								1522.6658	1521.6585	1521.7140	-36.45	110	122	1	---	K.FGSFDAFKEEFAK.A
								1881.9233	1880.9160	1880.9843	-36.31	54	70	0	---	K.SVEELVANLNEVPEAIR.T
								1881.9233	1880.9161	1880.9843	-36.28	54	70	0	60	K.SVEELVANLNEVPEAIR.T
								2251.0105	2250.0032	2250.0858	-36.69	179	197	0	19	R.RPDYIGAFWNVVDWNAAEK.R
								2251.0105	2250.0032	2250.0858	-36.69	179	197	0	---	R.RPDYIGAFWNVVDWNAAEK.R
								2432.1050	2431.0977	2431.1880	-37.14	32	53	0	---	K.HHNTYITNLNAALEGHAELADK.S
								2432.1050	2431.0977	2431.1880	-37.13	32	53	0	---	K.HHNTYITNLNAALEGHAELADK.S

								2451.1177	2450.1104	2450.2019	-37.33	159	178	0	---	K.TPVIGLDVWEHAYYLNYQNR.R
								2451.1177	2450.1104	2450.2019	-37.32	159	178	0	---	K.TPVIGLDVWEHAYYLNYQNR.R
								2468.1221	2467.1148	2467.2172	-41.49	2	22	1	---	M.AKHELPNLPYAYDALEPHFDK.E
								2468.1221	2467.1148	2467.2172	-41.48	2	22	1	---	M.AKHELPNLPYAYDALEPHFDK.E
								3332.4827	3331.4754	3331.5997	-37.31	128	158	0	---	R.FGSGWAWLVVNNGELEVTSTPNQDSPLTEGK.T
	**2.**	145	gi|434377442	superoxide dismutase [Bacillus thuringiensis HD-789]	22649											
	**3.**	145	gi|306447497	manganese superoxide dismutase [Bacillus sp. MHS47]	22616											
	**4.**	145	gi|488102083	superoxide dismutase [Bacillus cereus]	22607											
	**5.**	144	gi|301055769	Mn containing superoxide dismutase [Bacillus cereus biovar anth	23522											
	**6.**	143	gi|30022355	superoxide dismutase [Mn] [Bacillus cereus ATCC 14579]	24738											
	**7.**	139	gi|446974782	superoxide dismutase [Bacillus anthracis]	22680											
	**8.**	139	gi|446974769	superoxide dismutase [Bacillus cereus]	22708											
	**9.**	139	gi|52141224	superoxide dismutase [Bacillus cereus E33L]	22676											
	**10.**	139	gi|446974768	superoxide dismutase [Bacillus cereus]	22664											
	**11.**	139	gi|384188348	superoxide dismutase (Mn) [Bacillus thuringiensis serovar chiner	22616											
	**12.**	138	gi|446974778	superoxide dismutase [Bacillus cereus]	22636											
	**13.**	138	gi|446974774	superoxide dismutase [Bacillus cereus]	22615											
	**14.**	138	gi|73535546	Chain A, Crystal Structure Of Soda-1 (Ba4499) From Bacillus A	23704											
	**15.**	119	gi|446974777	superoxide dismutase [Bacillus cereus]	22635											
	**16.**	119	gi|446974772	superoxide dismutase [Bacillus cereus]	22605											
	**17.**	119	gi|488040468	superoxide dismutase [Bacillus cereus]	22577											
	**18.**	113	gi|33465735	manganese dependent superoxide dismutase [Bacillus anthracis]	15211											
	**19.**	113	gi|33465743	manganese dependent superoxide dismutase [Bacillus thuringiens	15155											
	**20.**	113	gi|33465751	manganese dependent superoxide dismutase [Bacillus thuringiens	15269											
	**21.**	113	gi|33465747	manganese dependent superoxide dismutase [Bacillus thuringiens	15245											
	**22.**	113	gi|33465769	manganese dependent superoxide dismutase [Bacillus cereus]	15216											
	**23.**	112	gi|146262040	SOD [Bacillus subtilis]	15574											
	**24.**	108	gi|33465753	manganese dependent superoxide dismutase [Bacillus thuringiens	15239											
	**25.**	94	gi|517595629	superoxide dismutase [Bacillus sp. 105MF]	22605											
	**26.**	90	gi|446974783	superoxide dismutase [Bacillus cereus]	22599											
	**27.**	90	gi|489292824	superoxide dismutase [Bacillus]	22591											
	**28.**	90	gi|507045291	superoxide dismutase [Mn] 1 [Bacillus cereus]	22601											
	**29.**	90	gi|515718201	superoxide dismutase [Bacillus sp. FJAT-13831]	22571											
**16**	**1.**	172	gi|448519843	**Sod2 mitochondrial Mn-containing superoxide dismutase [Ca**	22311	2.2e-010	9	1129.5435	1128.5362	1128.6080	-63.63	183	191	0	72	K.ALWNVVNWK.E
								1129.5435	1128.5362	1128.6080	-63.60	183	191	0	---	K.ALWNVVNWK.E
								1446.7123	1445.7050	1445.8031	-67.84	119	132	0	84	K.LAGIQGSGWAFIVK.N
								1446.7123	1445.7050	1445.8031	-67.82	119	132	0	---	K.LAGIQGSGWAFIVK.N
								2238.9685	2237.9612	2238.1221	-71.90	178	195	2	---	K.ADYFKALWNVVNWKEAER.R
								2238.9685	2237.9612	2238.1221	-71.90	178	195	2	---	K.ADYFKALWNVVNWKEAER.R
								2282.9326	2281.9253	2282.1655	-105.23	29	48	2	---	K.HHKTYVDNLNKSIESAVEAK.S
								2282.9326	2281.9253	2282.1655	-105.22	29	48	2	1	K.HHKTYVDNLNKSIESAVEAK.S
								2283.8931	2282.8858	2283.1495	-115.48	29	48	2	---	K.HHKTYVDNLNKSIESAVEAK.S + Deamidated (NQ)
	**2.**	171	gi|354544167	hypothetical protein CPAR2_109280 [Candida parapsilosis]	25535											
	**3.**	118	gi|448118161	Piso0_001042 [Millerozyma farinosa CBS 7064]	25480											
	**4.**	102	gi|146418515	hypothetical protein PGUG_02952 [Meyerozyma guilliermondii .	25234											
	**5.**	96	gi|254566141	Mitochondrial superoxide dismutase, protects cells against oxyge	25158											
	**6.**	96	gi|126273994	manganese-superoxide dismutase [Scheffersomyces stipitis CBS	25191											
	**7.**	96	gi|363750191	hypothetical protein Ecym_2799 [Eremothecium cymbalariae DE	30691											
	**8.**	94	gi|149244102	superoxide dismutase, mitochondrial precursor [Lodderomyces el	25642											
	**9.**	93	gi|60760047	manganese superoxide dismutase, partial [Candida parapsilosis]	14702											
	**10.**	92	gi|513034132	AaceriADR160Wp [Saccharomycetaceae sp. 'Ashbya aceri']	25062											
	**11.**	92	gi|156622416	mitochondrial superoxide dismutase [Nakaseomyces delphensis]	26243											
	**12.**	91	gi|50426317	DEHA2G04818p [Debaryomyces hansenii CBS767]	25373											
	**13.**	91	gi|260947596	hypothetical protein CLUG_01554 [Clavispora lusitaniae ATCC	25175											
	**14.**	90	gi|2398587	MnSOD [Candida sp. HN95]	25269											
	**15.**	90	gi|344304453	manganese-superoxide dismutase [Spathaspora passalidarum NR	25098											
	**16.**	90	gi|302307542	ADR160Wp [Ashbya gossypii ATCC 10895]	25224											
	**17.**	90	gi|562970061	Superoxide dismutase [Mn], mitochondrial [Ogataea parapolymo	25574											

	**18.**	90	gi|255730451	superoxide dismutase, mitochondrial precursor [Candida tropical	26220											
	**19.**	90	gi|77022402	hypothetical protein CaJ7_0018 [Candida albicans SC5314]	22720											
	**20.**	90	gi|544191907	Chain A, Crystal Structure Of The K184r, L185p Mutant Mang	22732											
	**21.**	90	gi|448123523	Piso0_000578 [Millerozyma farinosa CBS 7064]	23399											
	**22.**	90	gi|410078235	hypothetical protein KAFR_0C05730 [Kazachstania africana CE	26922											
**17**	**1.**	88	gi|308508467	**hypothetical protein CRE_08947 [Caenorhabditis remanei]**	117937	0.04	38	842.4681	841.4608	841.4406	24.0	1094	1101	1	---	R.GAGERQPK.D
								1130.5718	1129.5645	1129.6091	-39.49	345	355	1	14	R.LARETGDVAAK.T
								1130.5718	1129.5645	1129.6091	-39.48	345	355	1	---	R.LARETGDVAAK.T
								1213.5275	1212.5202	1212.5847	-53.16	642	653	0	---	K.GPQGAESSRPAR.A + Deamidated (NQ)
								1215.5536	1214.5463	1214.5640	-14.51	206	216	0	---	R.GADQEQSRPAR.A + Deamidated (NQ)
								1402.5436	1401.5363	1401.6484	-79.97	776	788	0	---	K.TQGAEQGQTRPAR.G + 3 Deamidated (NQ)
								1568.6721	1567.6648	1567.7776	-71.96	1035	1047	1	---	K.TQGLDQEHKPMKR.D + Deamidated (NQ)
								2039.0090	2038.0017	2037.9538	23.5	369	387	1	---	R.DTAHASAKTQGADQHKPMK.R + Deamidated (NQ); Oxidation (M)
								2055.9878	2054.9805	2054.9617	9.17	87	106	1	---	R.AAGDQPKTQGAEQGQSRPAR.G + 3 Deamidated (NQ)
								2057.0256	2056.0183	2055.9457	35.3	87	106	1	---	R.AAGDQPKTQGAEQGQSRPAR.G + 4 Deamidated (NQ)
								2057.0256	2056.0184	2055.9457	35.3	87	106	1	---	R.AAGDQPKTQGAEQGQSRPAR.G + 4 Deamidated (NQ)
								2071.0339	2070.0266	2069.9613	31.5	735	754	1	---	K.TQGAEEGQSRPARAAGEQPK.T + 3 Deamidated (NQ)
								2071.0339	2070.0267	2069.9613	31.6	735	754	1	---	K.TQGAEEGQSRPARAAGEQPK.T + 3 Deamidated (NQ)
								2079.0000	2077.9927	2078.0253	-15.67	413	432	1	---	R.AAGEQPKAQGAEQGQQRPAR.D + Deamidated (NQ)
								2081.0183	2080.0110	2079.9933	8.51	511	530	1	---	K.TQGAEQGQSRPARGAEVHAK.T + 3 Deamidated (NQ)
								2084.0237	2083.0164	2082.9891	13.1	1028	1046	1	---	R.GAEVNAKTQGLDQEHKPMK.R + 3 Deamidated (NQ)
								2086.9968	2085.9895	2086.0039	-6.88	727	747	1	---	R.ATAQAGAKTQGAEEGQSRPAR.A + 2 Deamidated (NQ)
								2093.0012	2091.9939	2092.0410	-22.48	776	795	1	---	K.TQGAEQGQTRPARGAEVHAK.T + Deamidated (NQ)
								2094.9800	2093.9727	2094.0090	-17.31	776	795	1	---	K.TQGAEQGQTRPARGAEVHAK.T + 3 Deamidated (NQ)
								2100.0156	2099.0083	2098.9841	11.6	1028	1046	1	---	R.GAEVNAKTQGLDQEHKPMK.R + 3 Deamidated (NQ); Oxidation (M)
								2108.9949	2107.9876	2108.0358	-22.87	237	256	0	---	R.AAGEQAKPQGAEQEQSRPAR.A
								2114.0261	2113.0188	2113.0148	1.91	1015	1034	1	---	K.TQGADQEQSRPARGAEVNAK.T + Deamidated (NQ)
								2116.9995	2115.9922	2115.9668	12.0	1015	1034	1	---	K.TQGADQEQSRPARGAEVNAK.T + 4 Deamidated (NQ)
								2128.0176	2127.0103	2127.0192	-4.17	594	613	1	---	K.TQGLEEQSRPSRAAGAQDPK.G + 2 Deamidated (NQ)
								2129.0125	2128.0052	2128.0032	0.95	594	613	1	---	K.TQGLEEQSRPSRAAGAQDPK.G + 3 Deamidated (NQ)
								2138.0532	2137.0459	2137.0148	14.6	264	283	1	---	K.TQGADQEQSRPARGAEVHAK.T + 2 Deamidated (NQ)
								2138.0532	2137.0459	2137.0148	14.6	264	283	1	---	K.TQGADQEQSRPARGAEVHAK.T + 2 Deamidated (NQ)
								2145.9556	2144.9483	2144.9934	-21.00	796	815	1	---	K.TQSADQQQSRPARGAEVNAK.T + 4 Deamidated (NQ)
								2147.9434	2146.9361	2147.0025	-30.92	984	1003	1	---	K.TQGAEQGQSRPARDTEMSAK.T
								2157.0286	2156.0213	2156.0094	5.55	748	767	1	---	R.AAGEQPKTQSADQQQSRPAR.A + 3 Deamidated (NQ)
								2163.9587	2162.9514	2162.9974	-21.27	984	1003	1	---	K.TQGAEQGQSRPARDTEMSAK.T + Oxidation (M)
								2175.9629	2174.9556	2174.9974	-19.22	284	303	1	---	K.TQGAEQGQSRPARDTEMNAK.T + Deamidated (NQ)
								2185.9214	2184.9141	2185.0835	-77.53	755	775	1	---	K.TQSADQQQSRPARASANIGAK.T + Deamidated (NQ)
								2194.9917	2193.9844	2193.9444	18.3	284	303	1	---	K.TQGAEQGQSRPARDTEMNAK.T + 4 Deamidated (NQ); Oxidation (M)
								2200.0259	2199.0186	2198.9974	9.64	121	140	1	---	K.TDGADQEQSRPARATEMHAK.T + Deamidated (NQ)
								2210.9998	2209.9925	2210.0134	-9.44	420	439	1	---	K.AQGAEQGQQRPARDTEMHAK.T + 2 Deamidated (NQ)
								2230.0657	2229.0584	2229.0080	22.6	257	276	1	---	R.ATEMHAKTQGADQEQSRPAR.G + 2 Deamidated (NQ); Oxidation (M)
								2283.0845	2282.0772	2282.0523	10.9	348	368	1	---	R.ETGDVAAKTQSADQNQHRPAR.D + 3 Deamidated (NQ)
**18a**	**1.**	102	gi|308508467	**hypothetical protein CRE_08947 [Caenorhabditis remanei]**	117937	0.0015	41	761.3458	760.3385	760.3980	-78.23	64	69	1	---	K.RFAPDR.A
								1130.5702	1129.5629	1129.6091	-40.90	345	355	1	12	R.LARETGDVAAK.T
								1130.5702	1129.5629	1129.6091	-40.89	345	355	1	---	R.LARETGDVAAK.T
								1213.5331	1212.5258	1212.5847	-48.54	642	653	0	---	K.GPQGAESSRPAR.A + Deamidated (NQ)
								1215.5514	1214.5441	1214.5640	-16.32	206	216	0	---	R.GADQEQSRPAR.A + Deamidated (NQ)
								1568.6794	1567.6721	1567.7776	-67.31	1035	1047	1	---	K.TQGLDQEHKPMKR.D + Deamidated (NQ)
								1628.6866	1627.6793	1627.7260	-28.69	614	627	0	---	K.GTQQADQDQHKPMK.R + Deamidated (NQ); Oxidation (M)
								2039.0062	2037.9989	2037.9538	22.2	369	387	1	---	R.DTAHASAKTQGADQHKPMK.R + Deamidated (NQ); Oxidation (M)
								2055.0049	2053.9976	2053.9777	9.71	87	106	1	---	R.AAGDQPKTQGAEQGQSRPAR.G + 2 Deamidated (NQ)
								2055.9927	2054.9854	2054.9617	11.6	87	106	1	---	R.AAGDQPKTQGAEQGQSRPAR.G + 3 Deamidated (NQ)
								2057.0205	2056.0132	2055.9457	32.8	87	106	1	---	R.AAGDQPKTQGAEQGQSRPAR.G + 4 Deamidated (NQ)
								2057.0205	2056.0132	2055.9457	32.9	87	106	1	---	R.AAGDQPKTQGAEQGQSRPAR.G + 4 Deamidated (NQ)

								2068.9993	2067.9920	2067.9933	-0.63	735	754	1	---	K.TQGAEEGQSRPARAAGEQPK.T + Deamidated (NQ)
								2071.0227	2070.0154	2069.9613	26.1	735	754	1	---	K.TQGAEEGQSRPARAAGEQPK.T + 3 Deamidated (NQ)
								2071.0227	2070.0154	2069.9613	26.1	735	754	1	---	K.TQGAEEGQSRPARAAGEQPK.T + 3 Deamidated (NQ)
								2078.9856	2077.9783	2078.0253	-22.60	413	432	1	---	R.AAGEQPKAQGAEQGQQRPAR.D + Deamidated (NQ)
								2081.0022	2079.9949	2079.9933	0.77	511	530	1	---	K.TQGAEQGQSRPARGAEVHAK.T + 3 Deamidated (NQ)
								2083.0117	2082.0044	2082.0051	-0.34	1028	1046	1	---	R.GAEVNAKTQGLDQEHKPMK.R + 2 Deamidated (NQ)
								2094.9746	2093.9673	2094.0090	-19.89	776	795	1	---	K.TQGAEQGQTRPARGAEVHAK.T + 3 Deamidated (NQ)
								2100.9856	2099.9783	2100.0195	-19.62	503	523	1	---	R.ASANIGAKTQGAEQGQSRPAR.G + 3 Deamidated (NQ)
								2110.9385	2109.9312	2110.0039	-34.42	237	256	0	---	R.AAGEQAKPQGAEQEQSRPAR.A + 2 Deamidated (NQ)
								2114.0134	2113.0061	2113.0148	-4.10	1015	1034	1	---	K.TQGADQEQSRPARGAEVNAK.T + Deamidated (NQ)
								2117.0081	2116.0008	2115.9668	16.1	1015	1034	1	---	K.TQGADQEQSRPARGAEVNAK.T + 4 Deamidated (NQ)
								2118.9517	2117.9444	2118.0566	-52.95	866	885	0	---	R.AAGEQAKPQGAEQEQPRPAR.G
								2128.0256	2127.0183	2127.0192	-0.41	594	613	1	---	K.TQGLEEQSRPSRAAGAQDPK.G + 2 Deamidated (NQ)
								2138.0457	2137.0384	2137.0148	11.0	264	283	1	---	K.TQGADQEQSRPARGAEVHAK.T + 2 Deamidated (NQ)
								2138.0457	2137.0384	2137.0148	11.1	264	283	1	---	K.TQGADQEQSRPARGAEVHAK.T + 2 Deamidated (NQ)
								2144.9399	2143.9326	2143.9617	-13.58	924	943	1	---	K.TQGADQEQSRPTRAAGDQPK.T + 4 Deamidated (NQ)
								2147.9402	2146.9329	2147.0025	-32.41	984	1003	1	---	K.TQGAEQGQSRPARDTEMSAK.T
								2157.0198	2156.0125	2156.0094	1.47	748	767	1	---	R.AAGEQPKTQSADQQQSRPAR.A + 3 Deamidated (NQ)
								2163.9536	2162.9463	2162.9974	-23.63	984	1003	1	---	K.TQGAEQGQSRPARDTEMSAK.T + Oxidation (M)
								2170.9333	2169.9260	2170.0185	-42.62	662	681	1	---	K.TQGTEMEHRPTRAAGQEAGK.G + Oxidation (M)
								2185.9404	2184.9331	2185.0835	-68.83	755	775	1	---	K.TQSADQQQSRPARASANIGAK.T + Deamidated (NQ)
								2191.9705	2190.9632	2190.9923	-13.29	284	303	1	---	K.TQGAEQGQSRPARDTEMNAK.T + Deamidated (NQ); Oxidation (M)
								2194.9988	2193.9915	2193.9444	21.5	284	303	1	---	K.TQGAEQGQSRPARDTEMNAK.T + 4 Deamidated (NQ); Oxidation (M)
								2200.9895	2199.9822	2199.9814	0.36	121	140	1	---	K.TDGADQEQSRPARATEMHAK.T + 2 Deamidated (NQ)
								2211.0039	2209.9966	2210.0134	-7.59	420	439	1	---	K.AQGAEQGQQRPARDTEMHAK.T + 2 Deamidated (NQ)
								2230.0745	2229.0672	2229.0080	26.6	257	276	1	---	R.ATEMHAKTQGADQEQSRPAR.G + 2 Deamidated (NQ); Oxidation (M)
								2282.0208	2281.0135	2281.0683	-24.01	348	368	1	---	R.ETGDVAAKTQSADQNQHRPAR.D + 2 Deamidated (NQ)
								2283.0732	2282.0659	2282.0523	5.97	348	368	1	---	R.ETGDVAAKTQSADQNQHRPAR.D + 3 Deamidated (NQ)
								2284.0759	2283.0686	2283.0363	14.2	348	368	1	---	R.ETGDVAAKTQSADQNQHRPAR.D + 4 Deamidated (NQ)
	**2.**	77	gi|88801757	**Secretion protein HlyD [Polaribacter irgensii 23-P]**	41353	0.5	14	1130.5702	1129.5629	1129.5615	1.24	176	186	0	41	R.EGALVSPSDQK.A
								1130.5702	1129.5629	1129.5615	1.25	176	186	0	---	R.EGALVSPSDQK.A
								1628.6866	1627.6793	1627.8028	-75.85	297	309	1	---	K.ATYEQQRNIMVFK.L + Deamidated (NQ)
								1644.6693	1643.6620	1643.7977	-82.55	297	309	1	---	K.ATYEQQRNIMVFK.L + Deamidated (NQ); Oxidation (M)
								1644.6693	1643.6620	1643.7977	-82.54	297	309	1	---	K.ATYEQQRNIMVFK.L + Deamidated (NQ); Oxidation (M)
								2055.0049	2053.9976	2054.1234	-61.24	8	25	1	---	R.SLLALSIFLVFTSCNKNK.T
								2055.9927	2054.9854	2055.1074	-59.36	8	25	1	---	R.SLLALSIFLVFTSCNKNK.T + Deamidated (NQ)
								2057.0205	2056.0132	2056.0914	-38.04	8	25	1	---	R.SLLALSIFLVFTSCNKNK.T + 2 Deamidated (NQ)
								2057.0205	2056.0132	2056.1415	-62.39	128	146	1	9	K.NIISTVLLETAKANLAQEK.A + Deamidated (NQ)
								2083.0117	2082.0044	2082.1547	-72.18	6	23	1	---	K.LRSLLALSIFLVFTSCNK.N + Deamidated (NQ)
								2147.9402	2146.9329	2147.1626	-106.97	224	243	0	---	K.LTNFPAVNLILANGSLYAEK.G
								2194.9988	2193.9915	2194.1131	-55.40	156	175	1	---	R.INYGTIRSQVDGNVGAINFR.E + Deamidated (NQ)
								2211.0039	2209.9966	2210.0815	-38.40	244	263	1	---	K.GKIETSTEQINQNTGTVSFR.A + Deamidated (NQ)
								2284.0759	2283.0686	2283.1383	-30.51	48	68	0	---	K.TVTGYQEYPTSIEGIVNSAVR.A
**18b**	**1.**	90	gi|32473681	**hypothetical protein RB5404 [Rhodopirellula baltica SH 1]**	41678	0.036	16	842.4670	841.4597	841.5246	-77.07	266	271	2	---	R.IERLRR.K
								856.4810	855.4737	855.4450	33.6	159	166	0	---	R.AELAAQPR.A + Deamidated (NQ)
								856.4810	855.4737	855.4450	33.6	159	166	0	36	R.AELAAQPR.A + Deamidated (NQ)
								870.4990	869.4917	869.4065	98.0	106	112	1	---	K.YRDASMK.G
								1011.5939	1010.5866	1010.5621	24.3	158	166	1	---	K.RAELAAQPR.A
								1011.5939	1010.5867	1010.5621	24.3	158	166	1	10	K.RAELAAQPR.A
								1344.5496	1343.5423	1343.6568	-85.26	85	96	0	---	K.LSDEIGLSPEQR.K + Deamidated (NQ)
								1344.5496	1343.5423	1343.6568	-85.23	85	96	0	---	K.LSDEIGLSPEQR.K + Deamidated (NQ)
								2138.0156	2137.0083	2137.0725	-30.03	183	200	1	---	R.QIQTELMRLSFDTELADK.L
								2138.0156	2137.0084	2137.0725	-30.01	183	200	1	---	R.QIQTELMRLSFDTELADK.L
								2147.9082	2146.9009	2146.9881	-40.60	301	316	2	---	K.IEEVRKDYYDDWMELK.K + Oxidation (M)
								2224.9934	2223.9861	2223.9630	10.4	273	292	0	---	K.HGDEFAMINGLAEDFGLDEK.A + Deamidated (NQ); Oxidation (M)
								2224.9934	2223.9861	2223.9630	10.4	273	292	0	---	K.HGDEFAMINGLAEDFGLDEK.A + Deamidated (NQ); Oxidation (M)
								2663.1221	2662.1148	2662.3007	-69.83	237	259	1	---	R.RNEVLQEVVMSSQTILTPEQSEK.L + 2 Deamidated (NQ); Oxidation (M)

								2663.1221	2662.1148	2662.3007	-69.82	237	259	1	---	R.RNEVLQEVVMSSQTILTPEQSEK.L + 2 Deamidated (NQ); Oxidation (M)
								2748.1816	2747.1743	2747.2782	-37.80	169	190	2	---	R.SYSPEQFQEMSKLRQIQTELMR.L + 3 Deamidated (NQ); Oxidation (M)
19a	1.	399	gi|156124994	**Tyr p 3 allergen [Tyrophagus putrescentiae]**	20059	2.9e-033	7	1465.6941	1464.6868	1464.7573	-48.10	94	107	0	92	R.YNTLTQGSGGQVIK.S
								1465.6941	1464.6868	1464.7573	-48.09	94	107	0	---	R.YNTLTQGSGGQVIK.S
								1866.9492	1865.9419	1866.0211	-42.42	170	188	0	---	K.EGGSSPSALQIVTVPIVGR.D
								1866.9492	1865.9419	1866.0211	-42.41	170	188	0	141	K.EGGSSPSALQIVTVPIVGR.D
								2274.1279	2273.1206	2273.2168	-42.32	40	62	0	---	R.IVGGVAATPGQAPYQVSLLYGGR.H
								2274.1279	2273.1207	2273.2168	-42.31	40	62	0	152	R.IVGGVAATPGQAPYQVSLLYGGR.H
								3330.4854	3329.4781	3329.6286	-45.18	63	93	0	---	R.HFCGGTIVSATWIVTAAHCVDGTSVSQISIR.Y
19b	1.	188	gi|156124994	**Tyr p 3 allergen [Tyrophagus putrescentiae]**	20059	5.6e-012	8	1465.7367	1464.7294	1464.7573	-19.01	94	107	0	95	R.YNTLTQGSGGQVIK.S
								1465.7367	1464.7294	1464.7573	-19.01	94	107	0	---	R.YNTLTQGSGGQVIK.S
								1866.9888	1865.9815	1866.0211	-21.22	170	188	0	52	K.EGGSSPSALQIVTVPIVGR.D
								1866.9888	1865.9815	1866.0211	-21.20	170	188	0	---	K.EGGSSPSALQIVTVPIVGR.D
								2274.1748	2273.1675	2273.2168	-21.69	40	62	0	26	R.IVGGVAATPGQAPYQVSLLYGGR.H
								2274.1748	2273.1675	2273.2168	-21.69	40	62	0	---	R.IVGGVAATPGQAPYQVSLLYGGR.H
								3330.5706	3329.5633	3329.6286	-19.61	63	93	0	---	R.HFCGGTIVSATWIVTAAHCVDGTSVSQISIR.Y
								3330.5706	3329.5633	3329.6286	-19.60	63	93	0	---	R.HFCGGTIVSATWIVTAAHCVDGTSVSQISIR.Y
20a	1.	210	gi|156124994	**Tyr p 3 allergen [Tyrophagus putrescentiae]**	20059	2.3e-014	10	1465.5936	1464.5863	1464.7573	-116.70	94	107	0	---	R.YNTLTQGSGGQVIK.S
								1466.5901	1465.5828	1465.7413	-108.11	94	107	0	35	R.YNTLTQGSGGQVIK.S + Deamidated (NQ)
								1466.5901	1465.5828	1465.7413	-108.10	94	107	0	---	R.YNTLTQGSGGQVIK.S + Deamidated (NQ)
								1680.8081	1679.8008	1679.8842	-49.66	94	109	1	---	R.YNTLTQGSGGQVIKSK.T
								1866.8916	1865.8843	1866.0211	-73.29	170	188	0	99	K.EGGSSPSALQIVTVPIVGR.D
								1866.8916	1865.8843	1866.0211	-73.29	170	188	0	---	K.EGGSSPSALQIVTVPIVGR.D
								1867.8867	1866.8794	1867.0051	-67.31	170	188	0	---	K.EGGSSPSALQIVTVPIVGR.D + Deamidated (NQ)
								2274.1028	2273.0955	2273.2168	-53.36	40	62	0	---	R.IVGGVAATPGQAPYQVSLLYGGR.H
								2275.0964	2274.0891	2274.2008	-49.12	40	62	0	---	R.IVGGVAATPGQAPYQVSLLYGGR.H + Deamidated (NQ)
								2275.0964	2274.0892	2274.2008	-49.10	40	62	0	64	R.IVGGVAATPGQAPYQVSLLYGGR.H + Deamidated (NQ)
20b	1.	147	gi|156124994	**Tyr p 3 allergen [Tyrophagus putrescentiae]**	20059	7.0e-008	6	1465.7504	1464.7431	1464.7573	-9.67	94	107	0	61	R.YNTLTQGSGGQVIK.S
								1465.7504	1464.7431	1464.7573	-9.65	94	107	0	---	R.YNTLTQGSGGQVIK.S
								1867.0048	1865.9975	1866.0211	-12.65	170	188	0	52	K.EGGSSPSALQIVTVPIVGR.D
								1867.0048	1865.9975	1866.0211	-12.63	170	188	0	---	K.EGGSSPSALQIVTVPIVGR.D
								2274.1907	2273.1834	2273.2168	-14.71	40	62	0	26	R.IVGGVAATPGQAPYQVSLLYGGR.H
								2274.1907	2273.1834	2273.2168	-14.69	40	62	0	---	R.IVGGVAATPGQAPYQVSLLYGGR.H
	2.	92	gi|325108307	**type VI secretion ATPase, ClpV1 family [Planctomyces brasil**	96614	0.02	23	806.4461	805.4388	805.4368	2.55	1	7	0	---	-.MVNVSLK.S + Oxidation (M)
								806.4461	805.4388	805.4368	2.57	1	7	0	49	-.MVNVSLK.S + Oxidation (M)
								842.4994	841.4921	841.4657	31.4	13	19	0	---	K.LNPITQR.T + Deamidated (NQ)
								1011.6353	1010.6280	1010.5509	76.3	203	212	0	1	R.QGGIDPVLGR.D
								1011.6353	1010.6280	1010.5509	76.3	203	212	0	---	R.QGGIDPVLGR.D
								1036.5145	1035.5072	1035.4291	75.4	830	838	0	---	R.CQEVESGAR.N + Deamidated (NQ)
								1036.5145	1035.5073	1035.5865	-76.57	771	779	0	---	K.TFKPAFLGR.C
								1367.6429	1366.6356	1366.7357	-73.22	408	418	2	---	K.RYISGRYLPDK.G
								1367.6429	1366.6357	1366.7357	-73.20	408	418	2	---	K.RYISGRYLPDK.G
								1465.7504	1464.7431	1464.8049	-42.16	401	413	2	---	R.DAVTLSKRYISGR.Y
								1465.7504	1464.7431	1464.8049	-42.14	401	413	2	---	R.DAVTLSKRYISGR.Y
								1516.6893	1515.6820	1515.7780	-63.35	213	225	0	---	R.DEEISQVVDILTR.R
								1516.6893	1515.6821	1515.7780	-63.33	213	225	0	---	R.DEEISQVVDILTR.R
								1867.0048	1865.9975	1865.9530	23.8	430	446	1	5	R.VAMSHQATPGQVTDLRR.H
								1867.0048	1865.9975	1865.9530	23.9	430	446	1	---	R.VAMSHQATPGQVTDLRR.H
								2256.1733	2255.1660	2255.0739	40.9	461	481	2	---	R.EVAAGADHAEELEAMAKEKEK.T
								2256.1733	2255.1661	2255.0739	40.9	461	481	2	---	R.EVAAGADHAEELEAMAKEKEK.T
								2274.1907	2273.1834	2273.0641	52.5	583	601	2	2	R.QRMEERVCGQSHGLETISR.S + Deamidated (NQ)

								2274.1907	2273.1834	2273.0641	52.5	583	601	2	---	R.QRMEERVCGQSHGLETISR.S + Deamidated (NQ)
								2292.0100	2291.0027	2291.1038	-44.12	361	380	1	---	R.RFQVVQVGEPAENAAMEMIR.G + Deamidated (NQ); Oxidation (M)
								2748.2837	2747.2764	2747.3575	-29.50	839	864	0	---	R.NVDNILNGTLLPELSAEFLEAMAAGK.S + 2 Deamidated (NQ); Oxidation (M
**20c**	**1.**	118	gi|156124994	**Tyr p 3 allergen [Tyrophagus putrescentiae]**	20059	5.6e-005	7	1465.6624	1464.6551	1464.7573	-69.77	94	107	0	65	R.YNTLTQGSGGQVIK.S
								1465.6624	1464.6551	1464.7573	-69.73	94	107	0	---	R.YNTLTQGSGGQVIK.S
								1866.8972	1865.8899	1866.0211	-70.29	170	188	0	---	K.EGGSSPSALQIVTVPIVGR.D
								1866.8972	1865.8899	1866.0211	-70.28	170	188	0	32	K.EGGSSPSALQIVTVPIVGR.D
								2274.0627	2273.0554	2273.2168	-71.00	40	62	0	---	R.IVGGVAATPGQAPYQVSLLYGGR.H
								2274.0627	2273.0555	2273.2168	-70.98	40	62	0	11	R.IVGGVAATPGQAPYQVSLLYGGR.H
								3330.4097	3329.4024	3329.6286	-67.92	63	93	0	---	R.HFCGGTIVSATWIVTAAHCVDGTSVSQISIR.Y
**21**	**1.**	95	gi|401626492	**hbt1p [Saccharomyces arboricola H-6]**	113679	0.0075	34	930.4099	929.4026	929.4203	-19.03	725	733	0	---	K.NNSGPTPSR.S + Deamidated (NQ)
								930.4099	929.4026	929.4203	-18.97	725	733	0	---	K.NNSGPTPSR.S + Deamidated (NQ)
								946.4490	945.4417	945.4920	-53.15	662	670	0	---	R.EGQIPGAFK.A
								1105.5204	1104.5131	1104.6139	-91.22	963	974	1	---	K.ISVGSVGSGKSK.H
								1244.5505	1243.5432	1243.5291	11.3	437	446	0	---	K.EPPHYNDNMK.N
								1246.6049	1245.5976	1245.4972	80.7	437	446	0	---	K.EPPHYNDNMK.N + 2 Deamidated (NQ)
								1248.5814	1247.5741	1247.6146	-32.45	872	882	1	---	K.VLDDDAPGYKR.E
								1248.5814	1247.5741	1247.6146	-32.43	872	882	1	---	K.VLDDDAPGYKR.E
								1264.5853	1263.5780	1263.6095	-24.91	183	193	1	---	K.TTPPANREYSK.V + Deamidated (NQ)
								1270.5526	1269.5453	1269.6929	-116.20	509	520	0	---	K.NLQDVVAPSSLK.D
								1289.6105	1288.6032	1288.6259	-17.57	568	579	1	---	R.SAGIPSRSEEQK.S + Deamidated (NQ)
								1478.5594	1477.5521	1477.6792	-86.03	585	597	0	---	K.NTNQSIVMPPTMK.D + 2 Deamidated (NQ); Oxidation (M)
								1478.5595	1477.5522	1477.6792	-85.99	585	597	0	---	K.NTNQSIVMPPTMK.D + 2 Deamidated (NQ); Oxidation (M)
								1505.5769	1504.5696	1504.6318	-41.31	474	485	0	---	R.DDILHDDDYQQK.N + Deamidated (NQ)
								1527.6198	1526.6125	1526.7801	-109.77	135	149	1	---	K.TTTTPAATHGQSKAR.T
								1543.5807	1542.5734	1542.6937	-77.96	975	986	1	---	K.HHHDHSHHQSRK.N + Deamidated (NQ)
								1549.6814	1548.6741	1548.6580	10.4	705	718	1	---	R.QGKYEFSNSSGNTK.L + 3 Deamidated (NQ)
								1625.6354	1624.6281	1624.7329	-64.49	102	116	0	---	R.SNVTSDVSPTHTDHK.S + Deamidated (NQ)
								1802.7524	1801.7451	1801.7867	-23.08	22	38	0	---	K.FDGKPGSYDSNSSSAQR.K
								1959.8029	1958.7956	1958.8143	-9.54	991	1007	0	---	K.GSDYDYNPNHSAEHTPR.H
								1975.7985	1974.7912	1974.9390	-74.85	580	597	1	---	K.SGEPKNTNQSIVMPPTMK.D + Deamidated (NQ); Oxidation (M)
								2025.9297	2024.9224	2024.9174	2.47	521	539	1	---	K.DNSFIAGSATQQKSEPNTK.L + 3 Deamidated (NQ)
								2067.8760	2066.8687	2066.9327	-30.98	756	776	0	---	K.NSHTAHLGTTGNVDAGTPGMK.A + 2 Deamidated (NQ)
								2067.8760	2066.8687	2066.9327	-30.97	756	776	0	---	K.NSHTAHLGTTGNVDAGTPGMK.A + 2 Deamidated (NQ)
								2081.9004	2080.8931	2080.9596	-31.96	756	776	0	---	K.NSHTAHLGTTGNVDAGTPGMK.A + Oxidation (M)
								2086.8589	2085.8516	2085.9313	-38.20	662	680	1	---	R.EGQIPGAFKADTFSNSMQR.Q + 3 Deamidated (NQ)
								2104.9448	2103.9375	2103.9207	7.99	437	453	1	---	K.EPPHYNDNMKNVQYPEK.T + 2 Deamidated (NQ)
								2132.0222	2131.0149	2130.9817	15.6	725	743	1	---	K.NNSGPTPSRSNFIDQIEPR.R + 3 Deamidated (NQ)
								2211.0171	2210.0098	2209.9606	22.3	585	603	1	---	K.NTNQSIVMPPTMKDEDLNK.G + 4 Deamidated (NQ); 2 Oxidation (M)
								2283.0840	2282.0767	2282.0597	7.45	756	778	1	---	K.NSHTAHLGTTGNVDAGTPGMKAK.T + 2 Deamidated (NQ); Oxidation (M)
								2473.0378	2472.0305	2472.0056	10.1	1	21	1	---	-.MNMNEPLSKDGQGEEEQNNFK.F + 2 Deamidated (NQ); 2 Oxidation (M)
								2620.1003	2619.0930	2619.3028	-80.08	509	533	1	---	K.NLQDVVAPSSLKDNSFIAGSATQQK.S + 2 Deamidated (NQ)
								2620.1003	2619.0931	2619.3028	-80.06	509	533	1	---	K.NLQDVVAPSSLKDNSFIAGSATQQK.S + 2 Deamidated (NQ)
								2991.2312	2990.2239	2990.2788	-18.36	540	567	0	---	K.LGQIEYESGVSTQSGNPQDVYGSQTANR.S + 6 Deamidated (NQ)
	**2.**	75	gi|304396978	**conserved hypothetical protein [Pantoea sp. aB]**	141476	0.73	29	944.4241	943.4168	943.4875	-74.99	854	861	0	---	R.GIWLNDAR.K
								944.4241	943.4168	943.4875	-74.96	854	861	0	---	R.GIWLNDAR.K
								946.4490	945.4417	945.4781	-38.43	232	238	1	---	R.WVRDNTR.L
								1105.5204	1104.5131	1104.4948	16.6	267	277	0	---	K.GGASWQGETGR.H
								1230.5856	1229.5783	1229.5888	-8.50	72	82	0	---	R.DLNVDLNENGK.L
								1232.6072	1231.5999	1231.6044	-3.69	1125	1137	0	15	K.GQIADVNTGTAGK.I + Deamidated (NQ)

								1232.6072	1231.5999	1231.6044	-3.67	1125	1137	0	---	K.GQIADVNTGTAGK.I + Deamidated (NQ)
								1246.6049	1245.5976	1245.6176	-16.02	28	37	0	---	R.LVMPHLDSYR.S + Oxidation (M)
								1264.5853	1263.5780	1263.5706	5.85	719	729	0	---	R.ANWFGQPMGVR.F + 2 Deamidated (NQ)
								1270.5526	1269.5453	1269.6565	-87.54	48	60	0	---	R.AGVPVNASQLEGK.W + Deamidated (NQ)
								1292.5460	1291.5387	1291.6772	-107.21	797	808	0	---	K.EVSSHLPAPLDK.N
								1478.5594	1477.5521	1477.6936	-95.75	1022	1035	0	---	K.YGQISAALQPEGDK.L + 2 Deamidated (NQ)
								1478.5595	1477.5522	1477.6936	-95.72	1022	1035	0	---	K.YGQISAALQPEGDK.L + 2 Deamidated (NQ)
								1543.5807	1542.5734	1542.6223	-31.66	482	495	0	---	R.GDFSYQQNSGGEPR.L + 2 Deamidated (NQ)
								1789.7731	1788.7658	1788.9145	-83.10	680	695	0	---	R.GNVDLNNNSLFIKPLK.T + 4 Deamidated (NQ)
								1802.7524	1801.7451	1801.8959	-83.67	1120	1137	1	---	K.THFGKGQIADVNTGTAGK.I + Deamidated (NQ)
								1975.7985	1974.7912	1974.8959	-53.01	1073	1090	0	---	K.GPNVNNATNWFGVNTPLR.D + 5 Deamidated (NQ)
								2025.9297	2024.9224	2025.0418	-58.97	745	763	1	---	K.LQGDWQPAKISEVPAAVSK.Q + 2 Deamidated (NQ)
								2086.8589	2085.8516	2086.0444	-92.39	182	201	0	---	R.AEGEVSLSSFTGQHGVVQVR.L
								2104.9448	2103.9375	2104.0549	-55.80	898	919	0	---	R.ASGGGSDFQTGGVTLPGNITLR.S
								2211.0171	2210.0098	2210.1082	-44.50	513	532	0	---	R.YFPVPLMGNSLTHYLSGAVK.G + Deamidated (NQ); Oxidation (M)
								2242.9800	2241.9727	2242.1382	-73.82	1104	1124	1	---	R.SAPWQPSAETLSGTLKTHFGK.G
								2299.0669	2298.0596	2298.1756	-50.48	992	1011	1	---	K.ASPQQAKNINFSQWPALQLR.C + 2 Deamidated (NQ)
								2473.0378	2472.0305	2472.2418	-85.44	1181	1203	0	---	R.TDNLLVDGLEADIAMQGNLDLVK.R + Oxidation (M)
								2601.1216	2600.1143	2600.2758	-62.11	48	71	1	---	R.AGVPVNASQLEGKWENFGPTLQVR.D + 4 Deamidated (NQ)
								2601.1216	2600.1143	2600.2758	-62.11	48	71	1	---	R.AGVPVNASQLEGKWENFGPTLQVR.D + 4 Deamidated (NQ)
								2615.1423	2614.1350	2614.3160	-69.21	1181	1204	1	---	R.TDNLLVDGLEADIAMQGNLDLVKR.Q + 2 Deamidated (NQ)
								2634.1199	2633.1126	2633.2849	-65.43	554	575	1	---	K.HNDGMFQVWVPLRQATYAFQPK.W + Deamidated (NQ)
								3051.3062	3050.2989	3050.3359	-12.12	1153	1178	0	---	R.FDFSDTFTNSFYFDSINGTAWIENGR.M
**22**	**1.**	200	gi|196039924	**alkaline serine protease, subtilase family [Bacillus cereus NVH**	42448	2.3e-013	11	1285.5898	1284.5825	1284.6462	-49.59	125	135	0	---	K.IQAPLAWDSQR.S + Deamidated (NQ)
								1813.8854	1812.8781	1812.9330	-30.28	211	228	0	85	R.VLDNQGSGTLDAVAQGIR.E
								1813.8854	1812.8781	1812.9330	-30.27	211	228	0	---	R.VLDNQGSGTLDAVAQGIR.E
								2305.1272	2304.1199	2304.1597	-17.28	46	67	1	---	K.QNASLSNVQSFHKSVGATVLSK.D + 3 Deamidated (NQ)
								2319.9670	2318.9597	2319.0655	-45.61	277	297	0	---	K.ANYPAYYSEVIAVASTDQSDR.K
								2319.9670	2318.9598	2319.0655	-45.59	277	297	0	24	K.ANYPAYYSEVIAVASTDQSDR.K
								2448.0869	2447.0796	2447.1604	-33.03	277	298	1	---	K.ANYPAYYSEVIAVASTDQSDRK.S
								2448.0869	2447.0796	2447.1604	-33.02	277	298	1	70	K.ANYPAYYSEVIAVASTDQSDRK.S
								2587.1528	2586.1455	2586.1915	-17.76	299	322	0	---	K.SSFSTYGSWVDVAAPGSNIYSTYK.G
								2619.1096	2618.1023	2618.3340	-88.48	237	261	0	---	K.VISLSLGAQNGGTALQQAVQYAWNK.G + 2 Deamidated (NQ)
								3567.6650	3566.6577	3566.7424	-23.73	323	357	0	---	K.GSTYQSLSGTSMATPHVAGVAALLANQGYSNTQIR.Q + Oxidation (M)
	**2.**	200	gi|118477781	thermitase [Bacillus thuringiensis str. Al Hakam]	42445											
	**3.**	200	gi|229091355	Alkaline protease A [Bacillus cereus Rock3-42]	42431											
	**4.**	200	gi|228914968	Alkaline protease A [Bacillus thuringiensis serovar pulsiensis BC	42473											
	**5.**	199	gi|229069887	Alkaline protease A [Bacillus cereus F65185]	42335											
	**6.**	199	gi|206971382	alkaline serine protease, subtilase family [Bacillus cereus AH113	42365											
	**7.**	196	gi|229115844	Alkaline protease A [Bacillus cereus Rock1-3]	41149											
	**8.**	196	gi|229096856	Alkaline protease A [Bacillus cereus Rock3-29]	41150											
	**9.**	196	gi|30262385	alkaline serine protease [Bacillus anthracis str. Ames]	42412											
	**10.**	196	gi|228945984	Alkaline protease A [Bacillus thuringiensis serovar monterrey BC	42412											
	**11.**	196	gi|423551861	thermitase [Bacillus cereus ISP3191]	42403											
	**12.**	196	gi|52143098	alkaline serine protease [Bacillus cereus E33L]	42426											
	**13.**	196	gi|423442868	thermitase [Bacillus cereus BAG4X2-1]	42439											
	**14.**	196	gi|407704822	LysR family transcriptional regulator [Bacillus thuringiensis MC	42351											
	**15.**	196	gi|423379832	thermitase [Bacillus cereus BAG1O-2]	42438											
	**16.**	196	gi|423624602	thermitase [Bacillus cereus VD148]	42365											
	**17.**	195	gi|196043577	alkaline serine protease, subtilase family [Bacillus cereus 03BB1	42390											
	**18.**	195	gi|225864343	alkaline serine protease, subtilase family [Bacillus cereus 03BB1	42418											
	**19.**	195	gi|229079554	Alkaline protease A [Bacillus cereus Rock4-2]	42393											
	**20.**	195	gi|228921076	Alkaline protease A [Bacillus thuringiensis serovar huazhongensi	42337											
	**21.**	191	gi|63029123	alkaline protease A [Bacillus thuringiensis serovar kurstaki]	19877											
	**22.**	111	gi|206974091	alkaline serine protease, subtilase family [Bacillus cereus H3081.	42376											
	**23.**	111	gi|222095966	thermitase [Bacillus cereus Q1]	42377											
	**24.**	106	gi|423575921	thermitase [Bacillus cereus MSX-D12]	42403											

	**25.**	103	gi|338172913	keratinase [Bacillus cereus]	39044											
	**26.**	102	gi|228985466	Alkaline protease A [Bacillus thuringiensis serovar tochigiensis B	42365											
	**27.**	98	gi|229144955	Alkaline protease A [Bacillus cereus BDRD-ST24]	42349											
	**28.**	98	gi|423587205	thermitase [Bacillus cereus VD045]	42367											
	**29.**	98	gi|423383757	thermitase [Bacillus cereus BAG1X1-2]	42351											
	**30.**	98	gi|30020448	thermitase [Bacillus cereus ATCC 14579]	42337											
	**31.**	98	gi|42781473	alkaline serine protease [Bacillus cereus ATCC 10987]	42352											
	**32.**	98	gi|423403015	thermitase [Bacillus cereus BAG2X1-2]	42377											
	**33.**	98	gi|47565736	alkaline protease A [Bacillus cereus G9241]	42308											
	**34.**	98	gi|229155957	Alkaline protease A [Bacillus cereus ATCC 4342]	42393											
	**35.**	97	gi|384180311	alkaline serine protease, subtilase family protein [Bacillus thuring	42334											
	**36.**	95	gi|301053899	alkaline serine protease, subtilase family [Bacillus cereus biovar a	42419											
	**37.**	95	gi|228939515	Alkaline protease A [Bacillus thuringiensis serovar berliner ATC	42337											
	**38.**	95	gi|423655153	thermitase [Bacillus cereus VD200]	42399											
	**39.**	94	gi|75762124	Thermitase [Bacillus thuringiensis serovar israelensis ATCC 356	28598											
	**40.**	94	gi|218897330	alkaline serine protease [Bacillus cereus G9842]	42291											
	**41.**	94	gi|228908127	Alkaline protease A [Bacillus thuringiensis IBL 200]	42313											
	**42.**	92	gi|152975948	peptidase S8/S53 subtilisin kexin sedolisin [Bacillus cytotoxicus	42731											
	**43.**	92	gi|423617379	thermitase [Bacillus cereus VD115]	42351											
	**44.**	92	gi|229102964	Alkaline protease A [Bacillus cereus Rock3-28]	42328											
	**45.**	86	gi|999627	Chain B, Refined 1.8 Angstroms Resolution Crystal Structure Of	8928											
23	**1.**	425	gi|196039924	**alkaline serine protease, subtilase family [Bacillus cereus NVH**	42448	7.3e-036	17	1012.4917	1011.4844	1011.5025	-17.92	367	375	0	---	K.ISGTGTYWK.N
								1285.6068	1284.5995	1284.6462	-36.35	125	135	0	---	K.IQAPLAWDSQR.S + Deamidated (NQ)
								1285.6068	1284.5995	1284.6462	-36.35	125	135	0	18	K.IQAPLAWDSQR.S + Deamidated (NQ)
								1329.6724	1328.6651	1328.7048	-29.87	262	276	0	---	K.GSVIVAAAGNAGNTK.A
								1813.9044	1812.8971	1812.9330	-19.79	211	228	0	---	R.VLDNQGSGTLDAVAQGIR.E
								1813.9044	1812.8971	1812.9330	-19.78	211	228	0	114	R.VLDNQGSGTLDAVAQGIR.E
								2320.0146	2319.0073	2319.0655	-25.08	277	297	0	---	K.ANYPAYYSEVIAVASTDQSDR.K
								2320.0147	2319.0074	2319.0655	-25.06	277	297	0	87	K.ANYPAYYSEVIAVASTDQSDR.K
								2448.1138	2447.1065	2447.1604	-22.05	277	298	1	95	K.ANYPAYYSEVIAVASTDQSDRK.S
								2448.1138	2447.1065	2447.1604	-22.03	277	298	1	---	K.ANYPAYYSEVIAVASTDQSDRK.S
								2587.2205	2586.2132	2586.1915	8.40	299	322	0	62	K.SSFSTYGSWVDVAAPGSNIYSTYK.G
								2587.2205	2586.2132	2586.1915	8.42	299	322	0	---	K.SSFSTYGSWVDVAAPGSNIYSTYK.G
								2618.2383	2617.2310	2617.3500	-45.45	237	261	0	---	K.VISLSLGAQNGGTALQQAVQYAWNK.G + Deamidated (NQ)
								2623.1711	2622.1638	2622.2700	-40.50	237	261	0	---	K.VISLSLGAQNGGTALQQAVQYAWNK.G + 6 Deamidated (NQ)
								2715.2178	2714.2105	2714.2864	-27.96	298	322	1	---	R.KSSFSTYGSWVDVAAPGSNIYSTYK.G
								3567.6831	3566.6758	3566.7424	-18.65	323	357	0	---	K.GSTYQSLSGTSMATPHVAGVAALLANQGYSNTQIR.Q + Oxidation (M)
								3567.6831	3566.6758	3566.7424	-18.65	323	357	0	13	K.GSTYQSLSGTSMATPHVAGVAALLANQGYSNTQIR.Q + Oxidation (M)
	**2.**	423	gi|118477781	thermitase [Bacillus thuringiensis str. Al Hakam]	42445											
	**3.**	423	gi|229091355	Alkaline protease A [Bacillus cereus Rock3-42]	42431											
	**4.**	423	gi|228914968	Alkaline protease A [Bacillus thuringiensis serovar pulsiensis BG	42473											
	**5.**	418	gi|30262385	alkaline serine protease [Bacillus anthracis str. Ames]	42412											
	**6.**	418	gi|228945984	Alkaline protease A [Bacillus thuringiensis serovar monterrey BG	42412											
	**7.**	418	gi|423551861	thermitase [Bacillus cereus ISP3191]	42403											
	**8.**	418	gi|52143098	alkaline serine protease [Bacillus cereus E33L]	42426											
	**9.**	417	gi|423624602	thermitase [Bacillus cereus VD148]	42365											
	**10.**	416	gi|229069887	Alkaline protease A [Bacillus cereus F65185]	42335											
	**11.**	416	gi|206971382	alkaline serine protease, subtilase family [Bacillus cereus AH113	42365											
	**12.**	412	gi|229115844	Alkaline protease A [Bacillus cereus Rock1-3]	41149											
	**13.**	412	gi|229096856	Alkaline protease A [Bacillus cereus Rock3-29]	41150											
	**14.**	411	gi|423442868	thermitase [Bacillus cereus BAG4X2-1]	42439											
	**15.**	411	gi|407704822	LysR family transcriptional regulator [Bacillus thuringiensis MC	42351											
	**16.**	411	gi|423379832	thermitase [Bacillus cereus BAG1O-2]	42438											
	**17.**	410	gi|228921076	Alkaline protease A [Bacillus thuringiensis serovar huazhongensi	42337											
	**18.**	405	gi|196043577	alkaline serine protease, subtilase family [Bacillus cereus 03BB1	42390											
	**19.**	405	gi|225864343	alkaline serine protease, subtilase family [Bacillus cereus 03BB1	42418											
	**20.**	403	gi|229079554	Alkaline protease A [Bacillus cereus Rock4-2]	42393											

	**21.**	382	gi|63029123	alkaline protease A [Bacillus thuringiensis serovar kurstaki]	19877											
	**22.**	233	gi|206974091	alkaline serine protease, subtilase family [Bacillus cereus H3081.	42376											
	**23.**	233	gi|222095966	thermitase [Bacillus cereus Q1]	42377											
	**24.**	233	gi|423575921	thermitase [Bacillus cereus MSX-D12]	42403											
	**25.**	231	gi|338172913	keratinase [Bacillus cereus]	39044											
	**26.**	228	gi|228985466	Alkaline protease A [Bacillus thuringiensis serovar tochigiensis E	42365											
	**27.**	225	gi|301053899	alkaline serine protease, subtilase family [Bacillus cereus biovar a	42419											
	**28.**	222	gi|423587205	thermitase [Bacillus cereus VD045]	42367											
	**29.**	222	gi|423383757	thermitase [Bacillus cereus BAG1X1-2]	42351											
	**30.**	222	gi|30020448	thermitase [Bacillus cereus ATCC 14579]	42337											
	**31.**	222	gi|42781473	alkaline serine protease [Bacillus cereus ATCC 10987]	42352											
	**32.**	222	gi|423403015	thermitase [Bacillus cereus BAG2X1-2]	42377											
	**33.**	222	gi|423655153	thermitase [Bacillus cereus VD200]	42399											
	**34.**	222	gi|229155957	Alkaline protease A [Bacillus cereus ATCC 4342]	42393											
	**35.**	217	gi|229144955	Alkaline protease A [Bacillus cereus BDRD-ST24]	42349											
	**36.**	217	gi|47565736	alkaline protease A [Bacillus cereus G9241]	42308											
	**37.**	217	gi|228939515	Alkaline protease A [Bacillus thuringiensis serovar berliner ATC	42337											
	**38.**	208	gi|384180311	alkaline serine protease, subtilase family protein [Bacillus thuring	42334											
	**39.**	206	gi|75762124	Thermitase [Bacillus thuringiensis serovar israelensis ATCC 356	28598											
	**40.**	204	gi|228908127	Alkaline protease A [Bacillus thuringiensis IBL 200]	42313											
	**41.**	203	gi|218897330	alkaline serine protease [Bacillus cereus G9842]	42291											
	**42.**	203	gi|229102964	Alkaline protease A [Bacillus cereus Rock3-28]	42328											
	**43.**	166	gi|423617379	thermitase [Bacillus cereus VD115]	42351											
	**44.**	121	gi|152975948	peptidase S8/S53 subtilisin kexin sedolisin [Bacillus cytotoxicus	42731											
	**45.**	110	gi|999627	Chain B, Refined 1.8 Angstroms Resolution Crystal Structure Of	8928											
	**46.**	105	gi|1942351	Chain A, Crystal Structure Of The First Active Autolysate Form	13569											
	**47.**	103	gi|402557411	alkaline serine protease [Bacillus cereus FRI-35]	42281											
	**48.**	103	gi|110590762	Chain A, Trypsin In Complex With Borate	24143											
	**49.**	103	gi|2914482	Chain A, Complex Of The Second Kunitz Domain Of Tissue Fac	24145											
	**50.**	103	gi|494360	Chain A, The Refined 1.6 Angstroms Resolution Crystal Structur	24143											
**24**	**1.**	144	gi|218901795	**neutral protease Npr599 [Bacillus cereus AH820]**	60941	9.2e-008	16	841.4404	840.4331	840.4494	-19.37	513	519	0	---	K.VGAIYYR.A
								841.4404	840.4331	840.4494	-19.33	513	519	0	---	K.VGAIYYR.A
								852.3679	851.3606	851.3701	-11.15	330	335	0	---	K.TYDYYK.A
								878.4546	877.4473	877.4294	20.4	352	359	0	---	K.STVHYGSK.Y
								1084.5590	1083.5517	1083.5713	-18.04	511	519	1	---	K.DKVGAIYYR.A
								1086.5341	1085.5268	1085.5393	-11.51	286	295	0	---	R.GATIFTYDAK.N
								1237.5465	1236.5392	1236.5523	-10.61	461	470	1	---	K.YGDPDHYSKR.Y
								1850.8392	1849.8319	1849.8595	-14.91	520	535	0	---	R.ANTQYFTQSTTFSQAR.A
								1850.8392	1849.8320	1849.8595	-14.89	520	535	0	63	R.ANTQYFTQSTTFSQAR.A
								1934.8884	1933.8811	1933.9130	-16.48	471	489	0	---	R.YTGTSDNGGVHTNSGIINK.A
								2033.9441	2032.9368	2032.9701	-16.39	268	285	0	45	K.SLNTTLSASSYYLQDNTR.G
								2033.9441	2032.9368	2032.9701	-16.39	268	285	0	---	K.SLNTTLSASSYYLQDNTR.G
								2062.0420	2061.0347	2061.0742	-19.15	536	557	0	---	R.AGLVQAAADLYGASSAEVAAVK.Q
								2076.0273	2075.0200	2075.0800	-28.90	490	510	0	---	K.AAYLLANGGTHYGVTVNGIGK.D
								2078.0320	2077.0247	2077.0480	-11.21	490	510	0	---	K.AAYLLANGGTHYGVTVNGIGK.D + 2 Deamidated (NQ)
								3400.5789	3399.5716	3399.6048	-9.75	298	329	0	---	R.TTLPGTLWVDADNVFNAAYDAAAVDAHYYAGK.T
	**2.**	144	gi|229188808	Bacillolysin [Bacillus cereus ATCC 10876]	60929											
	**3.**	144	gi|228957019	Bacillolysin [Bacillus thuringiensis serovar pakistani str. T13001	60931											
	**4.**	144	gi|228925788	Bacillolysin [Bacillus thuringiensis serovar pondicheriensis BGS(	60955											
	**5.**	138	gi|473953	hydrolase [Lactobacillus sp.]	60927											
	**6.**	137	gi|229120250	Bacillolysin [Bacillus cereus 95/8201]	62720											
	**7.**	134	gi|30260755	neutral protease [Bacillus anthracis str. Ames]	60967											
	**8.**	134	gi|196041391	neutral protease Npr599 [Bacillus cereus NVH0597-99]	60955											
	**9.**	134	gi|196046786	neutral protease Npr599 [Bacillus cereus 03BB108]	60923											
	**10.**	134	gi|225862572	neutral protease Npr599 [Bacillus cereus 03BB102]	60865											
	**11.**	134	gi|228932028	Bacillolysin [Bacillus thuringiensis serovar andalousiensis BGSC	60953											
	**12.**	134	gi|118476285	thermolysin [Bacillus thuringiensis str. Al Hakam]	60953											

	**13.**	133	gi|229182930	Bacillolysin [Bacillus cereus BGSC 6E1]	62730											
	**14.**	133	gi|229089662	Bacillolysin [Bacillus cereus Rock3-42]	62760											
	**15.**	133	gi|228913289	Bacillolysin [Bacillus thuringiensis serovar pulsiensis BGSC 4C(	62746											
	**16.**	128	gi|152974346	thermolysin [Bacillus cytotoxicus NVH 391-98]	61197											
	**17.**	125	gi|196035672	neutral protease Npr599 [Bacillus cereus W]	61067											
	**18.**	125	gi|52144711	bacillolysin (thermolysin-like metalloprotease, peptidase M4) [Ba	60981											
	**19.**	125	gi|49480192	bacillolysin (thermolysin-like metalloprotease, peptidase M4) [Ba	61023											
	**20.**	124	gi|228963694	Bacillolysin [Bacillus thuringiensis serovar sotto str. T04001]	60749											
	**21.**	124	gi|423363644	bacillolysin [Bacillus cereus VD022]	60807											
	**22.**	105	gi|999627	Chain B, Refined 1.8 Angstroms Resolution Crystal Structure Of	8928											
	**23.**	103	gi|110590762	Chain A, Trypsin In Complex With Borate	24143											
	**24.**	103	gi|2914482	Chain A, Complex Of The Second Kunitz Domain Of Tissue Fac	24145											
	**25.**	103	gi|494360	Chain A, The Refined 1.6 Angstroms Resolution Crystal Structu	24143											
	**26.**	102	gi|157878102	Chain E, Complex Of Eeti-Ii With Porcine Trypsin	24841											
	**27.**	102	gi|136429	RecName: Full=Trypsin; Flags: Precursor	25078											
	**28.**	101	gi|242253868	trypsinogen precursor [Sus scrofa]	26549											
	**29.**	100	gi|1942351	Chain A, Crystal Structure Of The First Active Autolysate Form	13569											
	**30.**	98	gi|315583496	Chain A, The Bowman-Birk Type Inhibitor From Mung Bean In	24115											
	**31.**	97	gi|3318722	Chain E, Leech-Derived Tryptase InhibitorTRYPSIN COMPLE	24142											
	**32.**	93	gi|423578934	bacillolysin [Bacillus cereus VD014]	60932											
	**33.**	93	gi|423653480	bacillolysin [Bacillus cereus VD200]	60962											
	**34.**	92	gi|228983801	Bacillolysin [Bacillus thuringiensis serovar tochigiensis BGSC 4	60771											
	**35.**	92	gi|76364030	neutral protease [Bacillus cereus]	33748											
	**36.**	89	gi|228919457	Bacillolysin [Bacillus thuringiensis serovar huazhongensis BGSC	60946											
	**37.**	88	gi|71835110	neutral protease [Bacillus cereus]	60869											
	**38.**	88	gi|423645776	bacillolysin [Bacillus cereus VD166]	60833											
	**39.**	88	gi|423387746	bacillolysin [Bacillus cereus BAG1X1-2]	60817											
	**40.**	87	gi|229028394	Bacillolysin [Bacillus cereus AH1271]	60833											
	**41.**	87	gi|229159680	Bacillolysin [Bacillus cereus R309803]	60619											
	**42.**	86	gi|228906349	Bacillolysin [Bacillus thuringiensis IBL 200]	60705											
**25**	**1.**	413	gi|218901795	**neutral protease Npr599 [Bacillus cereus AH820]**	60941	1.2e-034	24	841.4445	840.4372	840.4494	-14.45	513	519	0	---	K.VGAIYYR.A
								841.4445	840.4372	840.4494	-14.43	513	519	0	14	K.VGAIYYR.A
								852.3682	851.3609	851.3701	-10.80	330	335	0	---	K.TYDYYK.A
								878.4279	877.4206	877.4294	-9.98	352	359	0	---	K.STVHYGSK.Y
								1081.4480	1080.4407	1080.4512	-9.73	461	469	0	---	K.YGDPDHYSK.R
								1084.5612	1083.5539	1083.5713	-16.01	511	519	1	---	K.DKVGAIYYR.A
								1086.5388	1085.5315	1085.5393	-7.18	286	295	0	---	R.GATIFTYDAK.N
								1086.5388	1085.5315	1085.5393	-7.17	286	295	0	28	R.GATIFTYDAK.N
								1237.5490	1236.5417	1236.5523	-8.58	461	470	1	---	K.YGDPDHYSKR.Y
								1555.6941	1554.6868	1554.7929	-68.25	59	72	1	---	K.KAESVVFDYLNAAK.G + Deamidated (NQ)
								1733.7710	1732.7637	1732.7944	-17.71	433	447	0	---	R.NPDWEIGEDIYTPGK.A
								1850.8458	1849.8385	1849.8595	-11.34	520	535	0	---	R.ANTQYFTQSTTFSQAR.A
								1850.8458	1849.8386	1849.8595	-11.32	520	535	0	91	R.ANTQYFTQSTTFSQAR.A
								1934.8877	1933.8804	1933.9130	-16.84	471	489	0	---	R.YTGTSDNGGVHTNSGIINK.A
								1935.8816	1934.8743	1934.8970	-11.72	471	489	0	---	R.YTGTSDNGGVHTNSGIINK.A + Deamidated (NQ)
								2033.9508	2032.9435	2032.9701	-13.09	268	285	0	---	K.SLNTTLSASSYYLQDNTR.G
								2033.9508	2032.9435	2032.9701	-13.09	268	285	0	98	K.SLNTTLSASSYYLQDNTR.G
								2062.0461	2061.0388	2061.0742	-17.17	536	557	0	---	R.AGLVQAAADLYGASSAEVAAVK.Q
								2062.0461	2061.0389	2061.0742	-17.15	536	557	0	119	R.AGLVQAAADLYGASSAEVAAVK.Q
								2076.0491	2075.0418	2075.0800	-18.39	490	510	0	---	K.AAYLLANGGTHYGVTVNGIGK.D
								2077.0481	2076.0408	2076.0640	-11.16	490	510	0	---	K.AAYLLANGGTHYGVTVNGIGK.D + Deamidated (NQ)
								2078.0381	2077.0308	2077.0480	-8.27	490	510	0	---	K.AAYLLANGGTHYGVTVNGIGK.D + 2 Deamidated (NQ)
								2091.9858	2090.9785	2090.9981	-9.37	470	489	1	---	K.RYTGTSDNGGVHTNSGIINK.A + Deamidated (NQ)
								3400.5908	3399.5835	3399.6048	-6.25	298	329	0	---	R.TTLPGTLWVDADNVFNAAYDAAAVDAHYYAGK.T
	**2.**	413	gi|229188808	Bacillolysin [Bacillus cereus ATCC 10876]	60929											
	**3.**	413	gi|228957019	Bacillolysin [Bacillus thuringiensis serovar pakistani str. T13001	60931											
	**4.**	406	gi|228925788	Bacillolysin [Bacillus thuringiensis serovar pondicheriensis BGS(	60955											

	**5.**	405	gi|473953	hydrolase [Lactobacillus sp.]	60927											
	**6.**	404	gi|229120250	Bacillolysin [Bacillus cereus 95/8201]	62720											
	**7.**	391	gi|30260755	neutral protease [Bacillus anthracis str. Ames]	60967											
	**8.**	391	gi|196046786	neutral protease Npr599 [Bacillus cereus 03BB108]	60923											
	**9.**	391	gi|225862572	neutral protease Npr599 [Bacillus cereus 03BB102]	60865											
	**10.**	391	gi|228932028	Bacillolysin [Bacillus thuringiensis serovar andalousiensis BGSC	60953											
	**11.**	390	gi|229182930	Bacillolysin [Bacillus cereus BGSC 6E1]	62730											
	**12.**	390	gi|229089662	Bacillolysin [Bacillus cereus Rock3-42]	62760											
	**13.**	390	gi|228913289	Bacillolysin [Bacillus thuringiensis serovar pulsiensis BGSC 4C(	62746											
	**14.**	386	gi|196041391	neutral protease Npr599 [Bacillus cereus NVH0597-99]	60955											
	**15.**	308	gi|423578934	bacillolysin [Bacillus cereus VD014]	60932											
	**16.**	308	gi|423653480	bacillolysin [Bacillus cereus VD200]	60962											
	**17.**	301	gi|71835110	neutral protease [Bacillus cereus]	60869											
	**18.**	280	gi|118476285	thermolysin [Bacillus thuringiensis str. Al Hakam]	60953											
	**19.**	267	gi|423556492	bacillolysin [Bacillus cereus MC67]	60889											
	**20.**	267	gi|423473527	bacillolysin [Bacillus cereus BAG6O-2]	60889											
	**21.**	262	gi|423455855	bacillolysin [Bacillus cereus BAG5X1-1]	60891											
	**22.**	251	gi|196035672	neutral protease Npr599 [Bacillus cereus W]	61067											
	**23.**	251	gi|52144711	bacillolysin (thermolysin-like metalloprotease, peptidase M4) [Ba	60981											
	**24.**	251	gi|49480192	bacillolysin (thermolysin-like metalloprotease, peptidase M4) [Ba	61023											
	**25.**	250	gi|228963694	Bacillolysin [Bacillus thuringiensis serovar sotto str. T04001]	60749											
	**26.**	250	gi|423363644	bacillolysin [Bacillus cereus VD022]	60807											
	**27.**	205	gi|152974346	thermolysin [Bacillus cytotoxicus NVH 391-98]	61197											
	**28.**	182	gi|228919457	Bacillolysin [Bacillus thuringiensis serovar huazhongensis BGSC	60946											
	**29.**	165	gi|228983801	Bacillolysin [Bacillus thuringiensis serovar tochigiensis BGSC 4`	60771											
	**30.**	161	gi|76364030	neutral protease [Bacillus cereus]	33748											
	**31.**	160	gi|217331226	34 kDa fibrinolytic enzyme precursor [Bacillus pseudomycoides]	60883											
	**32.**	160	gi|229154303	Bacillolysin [Bacillus cereus ATCC 4342]	60925											
	**33.**	160	gi|423645776	bacillolysin [Bacillus cereus VD166]	60833											
	**34.**	160	gi|423387746	bacillolysin [Bacillus cereus BAG1X1-2]	60817											
	**35.**	159	gi|229159680	Bacillolysin [Bacillus cereus R309803]	60619											
	**36.**	154	gi|229194912	Bacillolysin [Bacillus cereus m1293]	60965											
	**37.**	154	gi|423577559	bacillolysin [Bacillus cereus MSX-D12]	60979											
	**38.**	154	gi|206974193	neutral protease Npr599 [Bacillus cereus H3081.97]	60951											
	**39.**	154	gi|384178557	bacillolysin (thermolysin-like metalloprotease, peptidase M4) [Ba	61053											
	**40.**	154	gi|12655808	neutral protease NprB [Bacillus thuringiensis serovar finitimus]	61126											
	**41.**	153	gi|423526189	bacillolysin [Bacillus cereus HuA4-10]	60762											
	**42.**	153	gi|365163641	bacillolysin [Bacillus sp. 7_6_55CFAA_CT2]	60851											
	**43.**	153	gi|423590359	bacillolysin [Bacillus cereus VD045]	60834											
	**44.**	153	gi|423646665	bacillolysin [Bacillus cereus VD169]	60870											
	**45.**	153	gi|30018788	bacillolysin [Bacillus cereus ATCC 14579]	60861											
	**46.**	149	gi|229028394	Bacillolysin [Bacillus cereus AH1271]	60833											
	**47.**	148	gi|228906349	Bacillolysin [Bacillus thuringiensis IBL 200]	60705											
	**48.**	148	gi|229101357	Bacillolysin [Bacillus cereus Rock3-28]	60748											
	**49.**	148	gi|229095250	Bacillolysin [Bacillus cereus Rock3-29]	60720											
	**50.**	148	gi|229056376	Bacillolysin [Bacillus cereus AH603]	60722											
**26**	**1.**	550	gi|229188808	**Bacillolysin [Bacillus cereus ATCC 10876]**	60929	2.3e-048	27	841.4441	840.4368	840.4494	-14.94	513	519	0	16	K.VGAIYYR.A
								841.4441	840.4368	840.4494	-14.93	513	519	0	---	K.VGAIYYR.A
								852.3649	851.3576	851.3701	-14.67	330	335	0	---	K.TYDYYK.A
								878.4261	877.4188	877.4294	-12.03	352	359	0	---	K.STVHYGSK.Y
								1081.4471	1080.4398	1080.4512	-10.56	461	469	0	---	K.YGDPDHYSK.R
								1084.5598	1083.5525	1083.5713	-17.30	511	519	1	---	K.DKVGAIYYR.A
								1086.5327	1085.5254	1085.5393	-12.80	286	295	0	---	R.GATIFTYDAK.N
								1086.5327	1085.5254	1085.5393	-12.79	286	295	0	36	R.GATIFTYDAK.N
								1099.5559	1098.5486	1098.5669	-16.65	341	351	0	---	R.NSINDAGAPLK.S
								1237.5457	1236.5384	1236.5523	-11.25	461	470	1	---	K.YGDPDHYSKR.Y
								1475.7323	1474.7250	1474.7052	13.4	220	231	1	---	K.YNKLDHVTNEDK.S
								1733.7727	1732.7654	1732.7944	-16.73	433	447	0	---	R.NPDWEIGEDIYTPGK.A

1850.8420	1849.8347	1849.8595	-13.39	520	535	0	---	R.ANTQYFTQSTTFSQAR.A
1850.8420	1849.8348	1849.8595	-13.37	520	535	0	95	R.ANTQYFTQSTTFSQAR.A
1890.8300	1889.8227	1889.9047	-43.36	60	76	1	---	K.AESVVFDYLNAAKGDYK.L + Deamidated (NQ)
1934.8827	1933.8754	1933.9130	-19.43	471	489	0	---	R.YTGTSDNGGVHTNSGIINK.A
1935.8779	1934.8706	1934.8970	-13.63	471	489	0	---	R.YTGTSDNGGVHTNSGIINK.A + Deamidated (NQ)
2033.9470	2032.9397	2032.9701	-14.96	268	285	0	---	K.SLNTTLSASSYYLQDNTR.G
2033.9470	2032.9397	2032.9701	-14.95	268	285	0	112	K.SLNTTLSASSYYLQDNTR.G
2062.0400	2061.0327	2061.0742	-20.12	536	557	0	---	R.AGLVQAAADLYGASSAEVAAVK.Q
2062.0400	2061.0328	2061.0742	-20.11	536	557	0	123	R.AGLVQAAADLYGASSAEVAAVK.Q
2076.0374	2075.0301	2075.0800	-24.05	490	510	0	81	K.AAYLLANGGTHYGVTVNGIGK.D
2076.0374	2075.0301	2075.0800	-24.03	490	510	0	---	K.AAYLLANGGTHYGVTVNGIGK.D
2078.0327	2077.0254	2077.0480	-10.87	490	510	0	---	K.AAYLLANGGTHYGVTVNGIGK.D + 2 Deamidated (NQ)
2090.9834	2089.9761	2090.0141	-18.17	470	489	1	---	K.RYTGTSDNGGVHTNSGIINK.A
2320.1580	2319.1507	2319.1859	-15.17	490	512	1	---	K.AAYLLANGGTHYGVTVNGIGKDK.V + Deamidated (NQ)
3400.6208	3399.6135	3399.6048	2.57	298	329	0	---	R.TTLPGTLWVDADNVFNAAYDAAAVDAHYYAGK.T

**2.**	550	gi|228957019	Bacillolysin [Bacillus thuringiensis serovar pakistani str. T13001	60931
**3.**	542	gi|218901795	neutral protease Npr599 [Bacillus cereus AH820]	60941
**4.**	534	gi|228925788	Bacillolysin [Bacillus thuringiensis serovar pondicheriensis BGS(	60955
**5.**	533	gi|473953	hydrolase [Lactobacillus sp.]	60927
**6.**	532	gi|229120250	Bacillolysin [Bacillus cereus 95/8201]	62720
**7.**	524	gi|229182930	Bacillolysin [Bacillus cereus BGSC 6E1]	62730
**8.**	524	gi|229089662	Bacillolysin [Bacillus cereus Rock3-42]	62760
**9.**	518	gi|30260755	neutral protease [Bacillus anthracis str. Ames]	60967
**10.**	518	gi|196046786	neutral protease Npr599 [Bacillus cereus 03BB108]	60923
**11.**	518	gi|225862572	neutral protease Npr599 [Bacillus cereus 03BB102]	60865
**12.**	518	gi|228932028	Bacillolysin [Bacillus thuringiensis serovar andalousiensis BGSC	60953
**13.**	516	gi|228913289	Bacillolysin [Bacillus thuringiensis serovar pulsiensis BGSC 4C(	62746
**14.**	511	gi|196041391	neutral protease Npr599 [Bacillus cereus NVH0597-99]	60955
**15.**	429	gi|423653480	bacillolysin [Bacillus cereus VD200]	60962
**16.**	421	gi|423578934	bacillolysin [Bacillus cereus VD014]	60932
**17.**	413	gi|71835110	neutral protease [Bacillus cereus]	60869
**18.**	396	gi|118476285	thermolysin [Bacillus thuringiensis str. Al Hakam]	60953
**19.**	307	gi|152974346	thermolysin [Bacillus cytotoxicus NVH 391-98]	61197
**20.**	289	gi|228919457	Bacillolysin [Bacillus thuringiensis serovar huazhongensis BGSC	60946
**21.**	288	gi|423556492	bacillolysin [Bacillus cereus MC67]	60889
**22.**	288	gi|423473527	bacillolysin [Bacillus cereus BAG6O-2]	60889
**23.**	283	gi|423455855	bacillolysin [Bacillus cereus BAG5X1-1]	60891
**24.**	278	gi|196035672	neutral protease Npr599 [Bacillus cereus W]	61067
**25.**	278	gi|52144711	bacillolysin (thermolysin-like metalloprotease, peptidase M4) [Ba	60981
**26.**	278	gi|49480192	bacillolysin (thermolysin-like metalloprotease, peptidase M4) [Ba	61023
**27.**	276	gi|228963694	Bacillolysin [Bacillus thuringiensis serovar sotto str. T04001]	60749
**28.**	276	gi|423363644	bacillolysin [Bacillus cereus VD022]	60807
**29.**	246	gi|228989744	Bacillolysin [Bacillus pseudomycoides DSM 12442]	61135
**30.**	211	gi|229083836	Bacillolysin [Bacillus cereus Rock3-44]	61201
**31.**	178	gi|423645776	bacillolysin [Bacillus cereus VD166]	60833
**32.**	177	gi|228983801	Bacillolysin [Bacillus thuringiensis serovar tochigiensis BGSC 4`	60771
**33.**	176	gi|254722123	neutral protease Npr599 [Bacillus anthracis str. A1055]	55760
**34.**	173	gi|76364030	neutral protease [Bacillus cereus]	33748
**35.**	172	gi|217331226	34 kDa fibrinolytic enzyme precursor [Bacillus pseudomycoides]	60883
**36.**	172	gi|229154303	Bacillolysin [Bacillus cereus ATCC 4342]	60925
**37.**	172	gi|423387746	bacillolysin [Bacillus cereus BAG1X1-2]	60817
**38.**	171	gi|229101357	Bacillolysin [Bacillus cereus Rock3-28]	60748
**39.**	171	gi|229095250	Bacillolysin [Bacillus cereus Rock3-29]	60720
**40.**	171	gi|365163641	bacillolysin [Bacillus sp. 7_6_55CFAA_CT2]	60851
**41.**	171	gi|423590359	bacillolysin [Bacillus cereus VD045]	60834
**42.**	171	gi|423646665	bacillolysin [Bacillus cereus VD169]	60870
**43.**	171	gi|30018788	bacillolysin [Bacillus cereus ATCC 14579]	60861
**44.**	171	gi|229159680	Bacillolysin [Bacillus cereus R309803]	60619
**45.**	166	gi|229194912	Bacillolysin [Bacillus cereus m1293]	60965

	**46.**	166	gi|423577559	bacillolysin [Bacillus cereus MSX-D12]	60979											
	**47.**	166	gi|206974193	neutral protease Npr599 [Bacillus cereus H3081.97]	60951											
	**48.**	166	gi|384178557	bacillolysin (thermolysin-like metalloprotease, peptidase M4) [Ba	61053											
	**49.**	166	gi|12655808	neutral protease NprB [Bacillus thuringiensis serovar finitimus]	61126											
	**50.**	166	gi|423526189	bacillolysin [Bacillus cereus HuA4-10]	60762											
**27a**	**1.**	217	gi|229188808	**Bacillolysin [Bacillus cereus ATCC 10876]**	60929	4.6e-015	18	841.4463	840.4390	840.4494	-12.32	513	519	0	10	K.VGAIYYR.A
								841.4463	840.4390	840.4494	-12.31	513	519	0	---	K.VGAIYYR.A
								852.3751	851.3678	851.3701	-2.69	330	335	0	---	K.TYDYYK.A
								878.4298	877.4225	877.4294	-7.82	352	359	0	---	K.STVHYGSK.Y
								1081.4631	1080.4558	1080.4512	4.25	461	469	0	---	K.YGDPDHYSK.R
								1084.5664	1083.5591	1083.5713	-11.21	511	519	1	---	K.DKVGAIYYR.A
								1086.5349	1085.5276	1085.5393	-10.78	286	295	0	---	R.GATIFTYDAK.N
								1086.5349	1085.5276	1085.5393	-10.77	286	295	0	24	R.GATIFTYDAK.N
								1237.5514	1236.5441	1236.5523	-6.64	461	470	1	---	K.YGDPDHYSKR.Y
								1475.7429	1474.7356	1474.7052	20.6	220	231	1	---	K.YNKLDHVTNEDK.S
								1850.8518	1849.8445	1849.8595	-8.09	520	535	0	---	R.ANTQYFTQSTTFSQAR.A
								1850.8518	1849.8445	1849.8595	-8.09	520	535	0	72	R.ANTQYFTQSTTFSQAR.A
								1934.9058	1933.8985	1933.9130	-7.48	471	489	0	---	R.YTGTSDNGGVHTNSGIINK.A
								2033.9585	2032.9512	2032.9701	-9.30	268	285	0	69	K.SLNTTLSASSYYLQDNTR.G
								2033.9585	2032.9512	2032.9701	-9.30	268	285	0	---	K.SLNTTLSASSYYLQDNTR.G
								2062.0549	2061.0476	2061.0742	-12.90	536	557	0	---	R.AGLVQAAADLYGASSAEVAAVK.Q
								2076.0586	2075.0513	2075.0800	-13.81	490	510	0	---	K.AAYLLANGGTHYGVTVNGIGK.D
								2078.0493	2077.0420	2077.0480	-2.88	490	510	0	---	K.AAYLLANGGTHYGVTVNGIGK.D + 2 Deamidated (NQ)
	**2.**	217	gi|228957019	Bacillolysin [Bacillus thuringiensis serovar pakistani str. T13001	60931											
	**3.**	211	gi|229182930	Bacillolysin [Bacillus cereus BGSC 6E1]	62730											
	**4.**	211	gi|229089662	Bacillolysin [Bacillus cereus Rock3-42]	62760											
	**5.**	211	gi|473953	hydrolase [Lactobacillus sp.]	60927											
	**6.**	211	gi|218901795	neutral protease Npr599 [Bacillus cereus AH820]	60941											
	**7.**	211	gi|228925788	Bacillolysin [Bacillus thuringiensis serovar pondicheriensis BGS(	60955											
	**8.**	210	gi|229120250	Bacillolysin [Bacillus cereus 95/8201]	62720											
	**9.**	206	gi|30260755	neutral protease [Bacillus anthracis str. Ames]	60967											
	**10.**	206	gi|196041391	neutral protease Npr599 [Bacillus cereus NVH0597-99]	60955											
	**11.**	206	gi|196046786	neutral protease Npr599 [Bacillus cereus 03BB108]	60923											
	**12.**	206	gi|225862572	neutral protease Npr599 [Bacillus cereus 03BB102]	60865											
	**13.**	206	gi|228932028	Bacillolysin [Bacillus thuringiensis serovar andalousiensis BGSC	60953											
	**14.**	206	gi|118476285	thermolysin [Bacillus thuringiensis str. Al Hakam]	60953											
	**15.**	205	gi|228913289	Bacillolysin [Bacillus thuringiensis serovar pulsiensis BGSC 4C(	62746											
	**16.**	182	gi|196035672	neutral protease Npr599 [Bacillus cereus W]	61067											
	**17.**	182	gi|52144711	bacillolysin (thermolysin-like metalloprotease, peptidase M4) [Ba	60981											
	**18.**	182	gi|49480192	bacillolysin (thermolysin-like metalloprotease, peptidase M4) [Ba	61023											
	**19.**	180	gi|228963694	Bacillolysin [Bacillus thuringiensis serovar sotto str. T04001]	60749											
	**20.**	180	gi|423363644	bacillolysin [Bacillus cereus VD022]	60807											
	**21.**	148	gi|152974346	thermolysin [Bacillus cytotoxicus NVH 391-98]	61197											
	**22.**	142	gi|423653480	bacillolysin [Bacillus cereus VD200]	60962											
	**23.**	137	gi|423578934	bacillolysin [Bacillus cereus VD014]	60932											
	**24.**	137	gi|71835110	neutral protease [Bacillus cereus]	60869											
	**25.**	132	gi|228919457	Bacillolysin [Bacillus thuringiensis serovar huazhongensis BGSC	60946											
	**26.**	120	gi|229056376	Bacillolysin [Bacillus cereus AH603]	60722											
	**27.**	120	gi|423526189	bacillolysin [Bacillus cereus HuA4-10]	60762											
	**28.**	119	gi|76364030	neutral protease [Bacillus cereus]	33748											
	**29.**	116	gi|365163641	bacillolysin [Bacillus sp. 7_6_55CFAA_CT2]	60851											
	**30.**	116	gi|423590359	bacillolysin [Bacillus cereus VD045]	60834											
	**31.**	116	gi|423645776	bacillolysin [Bacillus cereus VD166]	60833											
	**32.**	116	gi|423646665	bacillolysin [Bacillus cereus VD169]	60870											
	**33.**	116	gi|30018788	bacillolysin [Bacillus cereus ATCC 14579]	60861											
	**34.**	116	gi|229159680	Bacillolysin [Bacillus cereus R309803]	60619											
	**35.**	116	gi|423461401	bacillolysin [Bacillus cereus BAG5X2-1]	60770											

	**36.**	116	gi|423508566	bacillolysin [Bacillus cereus HuA2-1]	60765											
	**37.**	116	gi|228983801	Bacillolysin [Bacillus thuringiensis serovar tochigiensis BGSC 4`	60771											
	**38.**	114	gi|157832163	Chain A, The Structure Of Neutral Protease From Bacillus Cereu	33767											
	**39.**	114	gi|157831009	Chain A, Neutral Protease Mutant E144s	33725											
	**40.**	114	gi|423613966	bacillolysin [Bacillus cereus VD107]	60926											
	**41.**	113	gi|229114198	Bacillolysin [Bacillus cereus Rock1-3]	60690											
	**42.**	113	gi|229101357	Bacillolysin [Bacillus cereus Rock3-28]	60748											
	**43.**	113	gi|229095250	Bacillolysin [Bacillus cereus Rock3-29]	60720											
	**44.**	113	gi|423620754	bacillolysin [Bacillus cereus VD115]	60674											
	**45.**	113	gi|407708562	hypothetical protein MC28_5326 [Bacillus thuringiensis MC28]	60648											
	**46.**	112	gi|217331226	34 kDa fibrinolytic enzyme precursor [Bacillus pseudomycoides]	60883											
	**47.**	112	gi|229154303	Bacillolysin [Bacillus cereus ATCC 4342]	60925											
	**48.**	112	gi|423387746	bacillolysin [Bacillus cereus BAG1X1-2]	60817											
	**49.**	112	gi|229028394	Bacillolysin [Bacillus cereus AH1271]	60833											
	**50.**	112	gi|423421305	bacillolysin [Bacillus cereus BAG3X2-1]	60790											
**27b**	**1.**	187	gi|487962439	**bacillolysin [Bacillus cereus]**	62720	7.4e-017	17	841.4535	840.4463	840.4494	-3.68	528	534	0	10	K.VGAIYYR.A
								841.4536	840.4463	840.4494	-3.63	528	534	0	---	K.VGAIYYR.A
								1036.5344	1035.5271	1035.5535	-25.48	9	17	1	---	K.NLLGGRFMK.K + Deamidated (NQ)
								1036.5344	1035.5271	1035.5535	-25.46	9	17	1	---	K.NLLGGRFMK.K + Deamidated (NQ)
								1081.4601	1080.4528	1080.4512	1.45	476	484	0	35	K.YGDPDHYSK.R
								1081.4601	1080.4528	1080.4512	1.47	476	484	0	---	K.YGDPDHYSK.R
								1086.5463	1085.5390	1085.5393	-0.27	301	310	0	---	R.GATIFTYDAK.N
								1179.5997	1178.5924	1178.6594	-56.81	9	18	2	---	K.NLLGGRFMKK.K + Oxidation (M)
								1733.7952	1732.7879	1732.7944	-3.77	448	462	0	43	R.NPDWEIGEDIYTPGK.A
								1733.7952	1732.7879	1732.7944	-3.75	448	462	0	---	R.NPDWEIGEDIYTPGK.A
								1850.8561	1849.8488	1849.8595	-5.78	535	550	0	7	R.ANTQYFTQSTTFSQAR.A
								1850.8561	1849.8488	1849.8595	-5.77	535	550	0	---	R.ANTQYFTQSTTFSQAR.A
								1890.9553	1889.9480	1889.9047	22.9	75	91	1	---	K.AESVVFDYLNAAKGDYK.L + Deamidated (NQ)
								2033.9613	2032.9540	2032.9701	-7.93	283	300	0	21	K.SLNTTLSASSYYLQDNTR.G
								2033.9613	2032.9540	2032.9701	-7.93	283	300	0	---	K.SLNTTLSASSYYLQDNTR.G
								2062.0603	2061.0530	2061.0742	-10.28	551	572	0	42	R.AGLVQAAADLYGASSAEVAAVK.Q
								2062.0603	2061.0530	2061.0742	-10.28	551	572	0	---	R.AGLVQAAADLYGASSAEVAAVK.Q
	**2.**	186	gi|489380266	bacillolysin [Bacillus thuringiensis]	62746											
	**3.**	184	gi|446653024	bacillolysin [Bacillus cereus]	60929											
	**4.**	184	gi|446653038	bacillolysin [Bacillus cereus group]	60931											
	**5.**	184	gi|516270671	bacillolysin [Bacillus sp. WBUNB004]	60959											
	**6.**	176	gi|473953	hydrolase [Lactobacillus sp.]	60927											
	**7.**	176	gi|218901795	neutral protease Npr599 [Bacillus cereus AH820]	60941											
	**8.**	175	gi|30260755	neutral protease [Bacillus anthracis str. Ames]	60967											
	**9.**	175	gi|376264561	neutral protease [Bacillus cereus F837/76]	60923											
	**10.**	175	gi|225862572	neutral protease Npr599 [Bacillus cereus 03BB102]	60865											
	**11.**	175	gi|301052250	bacillolysin [Bacillus cereus biovar anthracis str. CI]	60953											
	**12.**	174	gi|487928602	bacillolysin [Bacillus cereus]	62730											
	**13.**	174	gi|487978018	bacillolysin [Bacillus cereus]	62760											
	**14.**	173	gi|446653048	bacillolysin [Bacillus thuringiensis]	60955											
	**15.**	172	gi|488123869	bacillolysin [Bacillus cereus]	60955											
	**16.**	162	gi|446653036	bacillolysin [Bacillus cereus]	60962											
	**17.**	157	gi|446653033	bacillolysin [Bacillus cereus]	60932											
	**18.**	152	gi|71835110	neutral protease [Bacillus cereus]	60869											
	**19.**	147	gi|488042588	bacillolysin [Bacillus cereus]	60889											
	**20.**	142	gi|488130606	bacillolysin [Bacillus cereus]	60891											
	**21.**	142	gi|488087935	bacillolysin [Bacillus cereus]	60889											
	**22.**	142	gi|507031799	bacillolysin [Bacillus cereus]	60891											
	**23.**	134	gi|118476285	thermolysin [Bacillus thuringiensis str. Al Hakam]	60953											
	**24.**	127	gi|152974346	thermolysin [Bacillus cytotoxicus NVH 391-98]	61197											
	**25.**	122	gi|493015624	bacillolysin [Bacillus pseudomycoides]	61135											
	**26.**	122	gi|517611709	bacillolysin [Bacillus sp. 95MFCvi2.1]	61137											
	**27.**	118	gi|487911202	bacillolysin [Bacillus cereus]	61067											

	**28.**	118	gi|52144711	bacillolysin (thermolysin-like metalloprotease, peptidase M4) [Ba	60981											
	**29.**	118	gi|49480192	bacillolysin (thermolysin-like metalloprotease, peptidase M4) [Ba	61023											
	**30.**	118	gi|507025683	bacillolysin [Bacillus cereus]	60809											
	**31.**	116	gi|402562373	bacillolysin (thermolysin-like metalloprotease, peptidase M4) [Ba	60749											
	**32.**	116	gi|446653049	bacillolysin [Bacillus cereus]	60807											
	**33.**	112	gi|446653032	bacillolysin [Bacillus thuringiensis]	60946											
	**34.**	109	gi|497565665	bacillolysin [Bacillus anthracis]	55760											
	**35.**	107	gi|517595375	bacillolysin [Bacillus sp. 105MF]	61031											
	**36.**	105	gi|446653040	bacillolysin [Bacillus sp. 7_6_55CFAA_CT2]	60851											
	**37.**	105	gi|446653035	bacillolysin [Bacillus cereus]	60833											
	**38.**	105	gi|446653034	bacillolysin [Bacillus cereus]	60870											
	**39.**	105	gi|30018788	bacillolysin [Bacillus cereus ATCC 14579]	60861											
	**40.**	104	gi|507062774	bacillolysin [Bacillus cereus]	60773											
	**41.**	104	gi|531064062	protease [uncultured bacterium]	33878											
	**42.**	103	gi|446653014	bacillolysin [Bacillus cereus]	60661											
	**43.**	103	gi|446653027	bacillolysin [Bacillus thuringiensis]	60705											
	**44.**	103	gi|446653026	bacillolysin [Bacillus cereus]	60692											
	**45.**	101	gi|446653039	bacillolysin [Bacillus cereus]	60834											
	**46.**	100	gi|157832163	Chain A, The Structure Of Neutral Protease From Bacillus Cereu	33767											
	**47.**	100	gi|157831009	Chain A, Neutral Protease Mutant E144s	33725											
	**48.**	100	gi|76364030	neutral protease [Bacillus cereus]	33748											
	**49.**	100	gi|446653015	bacillolysin [Bacillus cereus]	60619											
	**50.**	100	gi|446746902	bacillolysin [Bacillus cereus]	60790											
**28**	**1.**	350	gi|423367702	hypothetical protein IC3_02803 [Bacillus cereus VD142]	39489	2.3e-028	20	786.4120	785.4047	785.4647	-76.35	52	58	1	---	K.LKDVSPK.W
								835.4564	834.4491	834.4851	-43.08	302	308	0	---	K.ALDYIIK.G
								1239.6022	1238.5949	1238.6044	-7.65	318	328	0	44	K.LSNQSGYPAFR.G
								1239.6022	1238.5949	1238.6044	-7.63	318	328	0	---	K.LSNQSGYPAFR.G
								1277.6122	1276.6049	1276.7027	-76.57	124	134	0	---	R.FINSIQSLIDK.Y
								1304.5561	1303.5489	1303.5581	-7.11	341	350	0	43	K.NNFEFSNNYR.T
								1304.5562	1303.5489	1303.5581	-7.07	341	350	0	---	K.NNFEFSNNYR.T
								1305.5518	1304.5445	1304.5421	1.82	341	350	0	---	K.NNFEFSNNYR.T + Deamidated (NQ)
								1407.6899	1406.6826	1406.6653	12.3	329	340	0	---	R.GLMSWSINWDAK.N
								1636.9218	1635.9145	1635.9308	-9.97	159	173	0	60	K.NPTTPQIVNLISAIR.T
								1636.9218	1635.9145	1635.9308	-9.94	159	173	0	---	K.NPTTPQIVNLISAIR.T
								1651.7556	1650.7483	1650.7638	-9.39	59	73	0	---	K.WDVINVSFGETGGDR.S
								1651.7556	1650.7483	1650.7638	-9.38	59	73	0	43	K.WDVINVSFGETGGDR.S
								1776.8104	1775.8031	1775.8254	-12.55	74	89	0	---	R.STVEFSPVYGTDAEFK.S
								1776.8104	1775.8031	1775.8254	-12.54	74	89	0	91	R.STVEFSPVYGTDAEFK.S
								1946.9856	1945.9783	1946.0050	-13.73	35	51	0	---	K.LLVGYWHNFDNGTGIIK.L
								1964.0533	1963.0460	1963.1102	-32.71	102	121	0	---	K.VVLSIGGQNGVVLLPDNAAK.Q
								2092.1653	2091.1580	2091.2052	-22.55	101	121	1	---	K.KVVLSIGGQNGVVLLPDNAAK.Q
								2249.0435	2248.0362	2248.2539	-96.83	102	123	1	---	K.VVLSIGGQNGVVLLPDNAAKQR.F + Deamidated (NQ)
								2691.2759	2690.2686	2690.2931	-9.11	274	300	0	---	R.SDQVMIGLPAAPAAAPSGGYISPTEMK.K + 2 Oxidation (M)
	**2.**	344	gi|229191847	**Extracellular exochitinase [Bacillus cereus ATCC 10876]**	37774	9.2e-028	20	786.4120	785.4047	785.4647	-76.35	39	45	1	---	K.LKDVSPK.W
								834.4847	833.4774	833.5011	-28.35	289	295	0	---	K.ALNYIIK.G
								835.4564	834.4491	834.4851	-43.07	289	295	0	---	K.ALNYIIK.G + Deamidated (NQ)
								1239.6022	1238.5949	1238.6044	-7.65	305	315	0	44	K.LSNQSGYPAFR.G
								1239.6022	1238.5949	1238.6044	-7.63	305	315	0	---	K.LSNQSGYPAFR.G
								1277.6122	1276.6049	1276.7027	-76.57	111	121	0	---	R.FINSIQSLIDK.Y
								1304.5561	1303.5489	1303.5581	-7.11	328	337	0	43	K.NNFEFSNNYR.T
								1304.5562	1303.5489	1303.5581	-7.07	328	337	0	---	K.NNFEFSNNYR.T
								1305.5518	1304.5445	1304.5421	1.82	328	337	0	---	K.NNFEFSNNYR.T + Deamidated (NQ)
								1407.6899	1406.6826	1406.6653	12.3	316	327	0	---	R.GLMSWSINWDAK.N
								1636.9218	1635.9145	1635.9308	-9.97	146	160	0	60	K.NPTTPQIVNLISAIR.T
								1636.9218	1635.9145	1635.9308	-9.94	146	160	0	---	K.NPTTPQIVNLISAIR.T
								1651.7556	1650.7483	1650.7638	-9.39	46	60	0	---	K.WDVINVSFGETGGDR.S
								1651.7556	1650.7483	1650.7638	-9.38	46	60	0	43	K.WDVINVSFGETGGDR.S

								1776.8104	1775.8031	1775.8254	-12.55	61	76	0	---	R.STVEFSPVYGTDAEFK.S
								1776.8104	1775.8031	1775.8254	-12.54	61	76	0	91	R.STVEFSPVYGTDAEFK.S
								1946.9856	1945.9783	1946.0050	-13.73	22	38	0	---	K.LLVGYWHNFDNGTGIIK.L
								1964.0533	1963.0460	1963.1102	-32.71	89	108	0	---	K.VVLSIGGQNGVVLLPDNAAK.D
								2092.1653	2091.1580	2091.2052	-22.55	88	108	1	---	K.KVVLSIGGQNGVVLLPDNAAK.D
								2691.2759	2690.2686	2690.2931	-9.11	261	287	0	---	R.SDQVMIGLPAAPAAAPSGGYISPTEMK.K + 2 Oxidation (M)
	**3.**	342	gi|423412471	hypothetical protein IE1_01775 [Bacillus cereus BAG3O-2]	39531											
	**4.**	342	gi|228909547	Extracellular exochitinase [Bacillus thuringiensis IBL 200]	37871											
	**5.**	341	gi|228959933	Extracellular exochitinase [Bacillus thuringiensis serovar pakista	39432											
	**6.**	341	gi|229134524	Extracellular exochitinase [Bacillus cereus BDRD-ST196]	39500											
	**7.**	341	gi|423598973	hypothetical protein III_01775 [Bacillus cereus VD078]	39575											
	**8.**	328	gi|206969147	extracellular exochitinase Chi36 [Bacillus cereus AH1134]	39560											
	**9.**	319	gi|423458098	hypothetical protein IEI_01238 [Bacillus cereus BAG5X2-1]	39513											
	**10.**	319	gi|423401460	hypothetical protein ICW_01858 [Bacillus cereus BAG2X1-2]	39544											
	**11.**	319	gi|423477834	hypothetical protein IEO_03292 [Bacillus cereus BAG6X1-1]	39529											
	**12.**	298	gi|229117206	Extracellular exochitinase [Bacillus cereus Rock1-3]	38231											
	**13.**	298	gi|229098188	Extracellular exochitinase [Bacillus cereus Rock3-29]	38201											
	**14.**	297	gi|407706124	phage head-tail adaptor [Bacillus thuringiensis MC28]	38244											
	**15.**	297	gi|423378491	hypothetical protein IC9_01844 [Bacillus cereus BAG1O-2]	39573											
	**16.**	297	gi|423441547	hypothetical protein IEA_01877 [Bacillus cereus BAG4X2-1]	39543											
	**17.**	297	gi|423623204	hypothetical protein IK3_01802 [Bacillus cereus VD148]	39589											
	**18.**	296	gi|423616017	hypothetical protein IIO_01343 [Bacillus cereus VD115]	39597											
	**19.**	292	gi|229071221	Extracellular exochitinase [Bacillus cereus F65185]	37757											
	**20.**	291	gi|365159498	hypothetical protein HMPREF1014_01141 [Bacillus sp. 7_6_55	39531											
	**21.**	290	gi|423437182	hypothetical protein IE9_03363 [Bacillus cereus BAG4X12-1]	39458											
	**22.**	290	gi|228966646	Extracellular exochitinase [Bacillus thuringiensis serovar sotto st	37972											
	**23.**	288	gi|75762609	Exochitinase [Bacillus thuringiensis serovar israelensis ATCC 35	40405											
	**24.**	288	gi|402558976	extracellular exochitinase Chi36 [Bacillus thuringiensis HD-771]	39673											
	**25.**	288	gi|163941407	glycoside hydrolase family protein [Bacillus weihenstephanensis]	39473											
	**26.**	288	gi|423518356	hypothetical protein IG7_03426 [Bacillus cereus HuA2-4]	39487											
	**27.**	288	gi|402556153	extracellular exochitinase Chi36 [Bacillus cereus FRI-35]	39510											
	**28.**	285	gi|228954001	Extracellular exochitinase [Bacillus thuringiensis serovar kurstak	37771											
	**29.**	283	gi|262234624	chitinase A [Bacillus cereus]	39502											
	**30.**	283	gi|423425865	hypothetical protein IE5_03554 [Bacillus cereus BAG3X2-2]	39472											
	**31.**	282	gi|228902225	Extracellular exochitinase [Bacillus thuringiensis IBL 4222]	37954											
	**32.**	281	gi|218898822	extracellular exochitinase Chi36 [Bacillus cereus G9842]	39655											
	**33.**	281	gi|423669288	hypothetical protein IKO_02985 [Bacillus cereus VDM034]	39533											
	**34.**	275	gi|408778266	chitinase [Bacillus thuringiensis serovar israelensis]	39739											
	**35.**	261	gi|228940799	Extracellular exochitinase [Bacillus thuringiensis serovar berliner	37871											
	**36.**	253	gi|423522451	hypothetical protein IGC_01834 [Bacillus cereus HuA4-10]	40180											
	**37.**	251	gi|384187725	exochitinase [Bacillus thuringiensis serovar chinensis CT-43]	39572											
	**38.**	251	gi|423385230	hypothetical protein ICE_02976 [Bacillus cereus BAG1X1-2]	39608											
	**39.**	249	gi|118478946	glycosyl hydrolase family chitinase [Bacillus thuringiensis str. Al	40252											
	**40.**	247	gi|302148824	Chain A, Crystal Stricture Of Wild-Type Chitinase From Bacillu	36213											
	**41.**	247	gi|302148826	Chain A, Crystal Stricture Of D143a Chitinase In Complex With	36169											
	**42.**	247	gi|302148827	Chain A, Crystal Stricture Of E145q Chitinase In Complex With	36212											
	**43.**	247	gi|302148828	Chain A, Crystal Stricture Of E145qY227F CHITINASE IN CC	36196											
	**44.**	247	gi|302148829	Chain A, Crystal Stricture Of E145gY227F CHITINASE IN CC	36125											
	**45.**	245	gi|229146295	Extracellular exochitinase [Bacillus cereus BDRD-ST24]	37802											
	**46.**	245	gi|229047405	Extracellular exochitinase [Bacillus cereus AH676]	37796											
	**47.**	245	gi|229111196	Extracellular exochitinase [Bacillus cereus Rock1-15]	37797											
	**48.**	243	gi|296504228	exochitinase [Bacillus thuringiensis BMB171]	39471											
	**49.**	243	gi|423656586	hypothetical protein IKG_03574 [Bacillus cereus VD200]	39457											
	**50.**	243	gi|30021819	exochitinase [Bacillus cereus ATCC 14579]	39497											
**29**	**1.**	336	gi|156124994	**Tyr p 3 allergen [Tyrophagus putrescentiae]**	20059	5.8e-027	8	1465.7194	1464.7121	1464.7573	-30.84	94	107	0	85	R.YNTLTQGSGGQVIK.S
								1465.7194	1464.7121	1464.7573	-30.82	94	107	0	---	R.YNTLTQGSGGQVIK.S
								1467.7606	1466.7533	1466.7253	19.1	94	107	0	---	R.YNTLTQGSGGQVIK.S + 2 Deamidated (NQ)

								1866.9783	1865.9710	1866.0211	-26.85	170	188	0	128	K.EGGSSPSALQIVTVPIVGR.D
								1866.9783	1865.9710	1866.0211	-26.83	170	188	0	---	K.EGGSSPSALQIVTVPIVGR.D
								2274.1614	2273.1541	2273.2168	-27.59	40	62	0	110	R.IVGGVAATPGQAPYQVSLLYGGR.H
								2274.1614	2273.1541	2273.2168	-27.58	40	62	0	---	R.IVGGVAATPGQAPYQVSLLYGGR.H
								3330.5540	3329.5467	3329.6286	-24.58	63	93	0	---	R.HFCGGTIVSATWIVTAAHCVDGTSVSQISIR.Y
	**2.**	118	gi|51476390	hypothetical protein [Homo sapiens]	71353											
	**3.**	112	gi|119626071	albumin, isoform CRA_h [Homo sapiens]	70564											
	**4.**	112	gi|158258947	unnamed protein product [Homo sapiens]	71218											
	**5.**	112	gi|4502027	serum albumin preproprotein [Homo sapiens]	71317											
	**6.**	112	gi|23307793	serum albumin [Homo sapiens]	71344											
	**7.**	112	gi|60810141	albumin [synthetic construct]	71430											
	**8.**	111	gi|178345	alloalbumin Venezia [Homo sapiens]	71177											
	**9.**	111	gi|28592	serum albumin [Homo sapiens]	71316											
	**10.**	109	gi|55669910	Chain A, Crystal Structure Of The Ga Module Complexed With	67174											
	**11.**	108	gi|157830361	Chain A, Human Serum Albumin In A Complex With Myristic A	67988											
	**12.**	108	gi|228311905	Chain A, Crystal Structure Of Human Serum Albumin Complexe	68126											
	**13.**	108	gi|31615330	Chain A, Human Serum Albumin Mutant R218h Complexed Wit	68406											
	**14.**	108	gi|3212456	Chain A, Crystal Structure Of Human Serum Albumin	68425											
	**15.**	108	gi|122920512	Chain A, Human Serum Albumin Complexed With Myristate An	68408											
	**16.**	108	gi|332356380	albumin [Homo sapiens]	68484											
	**17.**	108	gi|31615331	Chain A, Human Serum Albumin Mutant R218p Complexed Wit	68366											
	**18.**	108	gi|78101694	Chain A, Human Serum Albumin Complexed With Myristate An	68398											
	**19.**	105	gi|426344604	PREDICTED: serum albumin [Gorilla gorilla gorilla]	71235											
	**20.**	105	gi|194391112	unnamed protein product [Homo sapiens]	61132											
	**21.**	105	gi|6013427	serum albumin precursor [Homo sapiens]	71176											
	**22.**	104	gi|189054552	unnamed protein product [Homo sapiens]	71343											
	**23.**	104	gi|194391080	unnamed protein product [Homo sapiens]	72370											
	**24.**	103	gi|353732021	HSA-GGGGS-PTH(1-34) [synthetic construct]	72837											
	**25.**	102	gi|119626074	albumin, isoform CRA_k [Homo sapiens]	48568											
	**26.**	101	gi|168988718	Chain A, Structure Of Human Serum Albumin With S-Naproxen	67773											
	**27.**	100	gi|28590	unnamed protein product [Homo sapiens]	71246											
	**28.**	98	gi|333609291	serum albumin precursor [Pongo abelii]	71430											
	**29.**	98	gi|332819547	PREDICTED: serum albumin [Pan troglodytes]	71259											
	**30.**	96	gi|343129219	IL-1Ra-GGGGS-HSA fusion protein [synthetic construct]	86065											
	**31.**	94	gi|27692693	ALB protein [Homo sapiens]	48641											
	**32.**	92	gi|11493459	PRO2619 [Homo sapiens]	58513											
	**33.**	92	gi|332233172	PREDICTED: serum albumin isoform 1 [Nomascus leucogenys]	71264											
	**34.**	92	gi|374432937	HSA-GGGGS-IL-1Ra fusion protein, partial [synthetic construct	86065											
	**35.**	90	gi|398359550	HSA-GGGGS-GH fusion protein, partial [synthetic construct]	91065											
**30**	**1.**	145	gi|229191670	**Extracellular solute-binding protein family 5 [Bacillus cereus**	63482	7.3e-008	23	758.3936	757.3863	757.4082	-28.95	532	538	0	---	R.GDAIVQR.Q
								912.4464	911.4391	911.4541	-16.48	133	139	0	---	K.DFVFAWK.R
								984.4958	983.4885	983.5076	-19.44	305	312	0	21	K.TPSTYFLR.L
								984.4958	983.4885	983.5076	-19.43	305	312	0	---	K.TPSTYFLR.L
								1038.4426	1037.4353	1037.4566	-20.54	247	254	0	---	K.NAQYWDNK.T
								1068.5829	1067.5756	1067.5553	19.1	133	140	1	---	K.DFVFAWKR.A
								1083.4926	1082.4853	1082.5033	-16.59	296	304	0	---	K.SNPDFFTQK.T
								1150.5668	1149.5595	1149.6030	-37.79	96	106	1	---	K.ATPGVAESYKK.S
								1190.6136	1189.6063	1189.6343	-23.50	258	267	0	---	K.LDEINFNVVK.D
								1248.5967	1247.5894	1247.6761	-69.50	417	427	1	---	K.VGEFLKGELEK.N
								1293.6196	1292.6123	1292.6361	-18.36	273	283	0	---	R.VNLYESGQIDR.S
								1293.6196	1292.6124	1292.6361	-18.34	273	283	0	20	R.VNLYESGQIDR.S
								1373.6639	1372.6566	1372.6874	-22.45	284	295	1	---	R.SGLTSEFVDKYK.S
								1528.7175	1527.7102	1527.7470	-24.10	543	556	0	---	K.GIVHHPVGGDYSYK.W
								1619.8053	1618.7980	1618.8388	-25.21	518	531	0	14	K.MLLEDAAIAPLYQR.G + Oxidation (M)
								1619.8053	1618.7980	1618.8388	-25.21	518	531	0	---	K.MLLEDAAIAPLYQR.G + Oxidation (M)
								1665.7496	1664.7423	1664.7756	-19.97	145	158	0	---	K.NTAAEYAYIMFDLK.N + Oxidation (M)
								2005.9156	2004.9083	2004.9674	-29.45	53	70	0	---	K.QVLNLTESQEIPSMDSAK.A + Oxidation (M)

								2078.9539	2077.9466	2078.0030	-27.13	141	158	1	---	R.AVDKNTAAEYAYIMFDLK.N + Oxidation (M)
								2087.0396	2086.0323	2086.0946	-29.87	341	360	0	---	K.GLTNVILNDGSTPADYLVPK.E
								2157.0134	2156.0061	2156.0572	-23.68	71	89	0	---	K.ATDQVSFLALNNVMEGLYR.L + Oxidation (M)
								2157.0134	2156.0062	2156.0572	-23.67	71	89	0	25	K.ATDQVSFLALNNVMEGLYR.L + Oxidation (M)
								2321.0732	2320.0659	2320.0900	-10.36	218	237	0	---	K.YGLESDTTLYNGPFTLTDWK.H
	**2.**	139	gi|206968984	oligopeptide ABC transporter, oligopeptide-binding protein [Baci	63447											
	**3.**	139	gi|228922291	Extracellular solute-binding protein family 5 [Bacillus thuringien	63451											
	**4.**	135	gi|999627	Chain B, Refined 1.8 Angstroms Resolution Crystal Structure Of	8928											
	**5.**	134	gi|229111007	Extracellular solute-binding protein family 5 [Bacillus cereus Ro	63424											
	**6.**	134	gi|229146152	Extracellular solute-binding protein family 5 [Bacillus cereus BD	63452											
	**7.**	129	gi|228966474	Extracellular solute-binding protein family 5 [Bacillus thuringien	63412											
	**8.**	129	gi|228902081	Extracellular solute-binding protein family 5 [Bacillus thuringien	63440											
	**9.**	128	gi|218898676	peptide ABC transporter oligopeptide-binding protein [Bacillus c	63398											
	**10.**	127	gi|1942351	Chain A, Crystal Structure Of The First Active Autolysate Form	13569											
	**11.**	126	gi|423616181	hypothetical protein IIO_01507 [Bacillus cereus VD115]	63455											
	**12.**	126	gi|423488675	hypothetical protein IEU_03298 [Bacillus cereus BtB2-4]	63456											
	**13.**	126	gi|423599132	hypothetical protein III_01934 [Bacillus cereus VD078]	63442											
	**14.**	125	gi|423469779	hypothetical protein IEM_01085 [Bacillus cereus BAG6O-2]	63423											
	**15.**	124	gi|118478827	oligopeptide transporter, periplasmic-binding protein [Bacillus th	66612											
	**16.**	124	gi|228928632	Extracellular solute-binding protein family 5 [Bacillus thuringien	63380											
	**17.**	124	gi|49478278	oligopeptide ABC transporter oligopeptide-binding protein [Bacil	63438											
	**18.**	123	gi|110590762	Chain A, Trypsin In Complex With Borate	24143											
	**19.**	123	gi|2914482	Chain A, Complex Of The Second Kunitz Domain Of Tissue Fac	24145											
	**20.**	123	gi|494360	Chain A, The Refined 1.6 Angstroms Resolution Crystal Structu	24143											
	**21.**	123	gi|3318722	Chain E, Leech-Derived Tryptase InhibitorTRYPSIN COMPLE	24142											
	**22.**	123	gi|157878102	Chain E, Complex Of Eeti-Ii With Porcine Trypsin	24841											
	**23.**	123	gi|136429	RecName: Full=Trypsin; Flags: Precursor	25078											
	**24.**	122	gi|242253868	trypsinogen precursor [Sus scrofa]	26549											
	**25.**	121	gi|229018773	Extracellular solute-binding protein family 5 [Bacillus cereus AH	63434											
	**26.**	120	gi|229168304	Extracellular solute-binding protein family 5 [Bacillus cereus AH	63480											
	**27.**	119	gi|423558889	hypothetical protein II3_04093 [Bacillus cereus MC67]	63674											
	**28.**	118	gi|315583496	Chain A, The Bowman-Birk Type Inhibitor From Mung Bean In	24115											
	**29.**	115	gi|423511583	hypothetical protein IG3_03080 [Bacillus cereus HuA2-1]	63588											
	**30.**	114	gi|423483194	hypothetical protein IEQ_02972 [Bacillus cereus BAG6X1-2]	63610											
	**31.**	112	gi|228959769	Extracellular solute-binding protein family 5 [Bacillus thuringien	63443											
	**32.**	103	gi|423611794	hypothetical protein IIM_02509 [Bacillus cereus VD107]	63485											
	**33.**	103	gi|423396086	hypothetical protein ICU_01780 [Bacillus cereus BAG2X1-1]	63628											
	**34.**	95	gi|423656441	hypothetical protein IKG_03429 [Bacillus cereus VD200]	63498											
	**35.**	93	gi|423635645	hypothetical protein IK7_02054 [Bacillus cereus VD156]	63508											
**31**	**1.**	113	gi|999627	Chain B, Refined 1.8 Angstroms Resolution Crystal Structure Of	8928											
	**2.**	105	gi|1942351	Chain A, Crystal Structure Of The First Active Autolysate Form	13569											
	**3.**	102	gi|110590762	Chain A, Trypsin In Complex With Borate	24143											
	**4.**	102	gi|2914482	Chain A, Complex Of The Second Kunitz Domain Of Tissue Fac	24145											
	**5.**	102	gi|494360	Chain A, The Refined 1.6 Angstroms Resolution Crystal Structu	24143											
	**6.**	102	gi|3318722	Chain E, Leech-Derived Tryptase InhibitorTRYPSIN COMPLE	24142											
	**7.**	101	gi|157878102	Chain E, Complex Of Eeti-Ii With Porcine Trypsin	24841											
	**8.**	101	gi|136429	RecName: Full=Trypsin; Flags: Precursor	25078											
	**9.**	100	gi|242253868	trypsinogen precursor [Sus scrofa]	26549											
	**10.**	99	gi|229191670	**Extracellular solute-binding protein family 5 [Bacillus cereus**	63482	0.0031	20	758.3970	757.3897	757.4082	-24.46	532	538	0	---	R.GDAIVQR.Q
								921.4432	920.4359	920.4352	0.79	365	372	1	---	K.SPDGKDFR.K
								984.5007	983.4934	983.5076	-14.47	305	312	0	13	K.TPSTYFLR.L
								984.5007	983.4934	983.5076	-14.44	305	312	0	---	K.TPSTYFLR.L
								1038.4604	1037.4531	1037.4566	-3.38	247	254	0	---	K.NAQYWDNK.T
								1068.5930	1067.5857	1067.5553	28.5	133	140	1	---	K.DFVFAWKR.A
								1083.5031	1082.4958	1082.5033	-6.89	296	304	0	---	K.SNPDFFTQK.T
								1190.6141	1189.6068	1189.6343	-23.08	258	267	0	---	K.LDEINFNVVK.D

								1248.6326	1247.6253	1247.6761	-40.72	417	427	1	---	K.VGEFLKGELEK.N
								1293.6305	1292.6232	1292.6361	-9.94	273	283	0	6	R.VNLYESGQIDR.S
								1293.6305	1292.6232	1292.6361	-9.93	273	283	0	---	R.VNLYESGQIDR.S
								1619.8168	1618.8095	1618.8388	-18.13	518	531	0	27	K.MLLEDAAIAPLYQR.G + Oxidation (M)
								1619.8168	1618.8095	1618.8388	-18.11	518	531	0	---	K.MLLEDAAIAPLYQR.G + Oxidation (M)
								1707.7554	1706.7481	1706.8475	-58.23	258	272	1	---	K.LDEINFNVVKDSGTR.V + Deamidated (NQ)
								1870.9462	1869.9389	1869.9796	-21.73	159	176	1	---	K.NAQAINEGKAELDTLGVK.A
								2087.0508	2086.0435	2086.0946	-24.50	341	360	0	---	K.GLTNVILNDGSTPADYLVPK.E
								2157.0315	2156.0242	2156.0572	-15.29	71	89	0	7	K.ATDQVSFLALNNVMEGLYR.L + Oxidation (M)
								2157.0315	2156.0242	2156.0572	-15.29	71	89	0	---	K.ATDQVSFLALNNVMEGLYR.L + Oxidation (M)
								2321.1208	2320.1135	2320.0900	10.2	218	237	0	---	K.YGLESDTTLYNGPFTLTDWK.H
								2343.0090	2342.0017	2342.2416	-102.42	518	538	1	---	K.MLLEDAAIAPLYQRGDAIVQR.Q
	**11.**	96	gi|315583496	Chain A, The Bowman-Birk Type Inhibitor From Mung Bean In	24115											
	**12.**	86	gi|206968984	oligopeptide ABC transporter, oligopeptide-binding protein [Baci	63447											
	**13.**	86	gi|228922291	Extracellular solute-binding protein family 5 [Bacillus thuringien	63451											
	**14.**	86	gi|228902081	Extracellular solute-binding protein family 5 [Bacillus thuringien	63440											

**32**	**1.**	175	gi|229188566	**1-pyrroline-5-carboxylate dehydrogenase [Bacillus cereus AT**	56445	7.3e-011	26	734.4076	733.4003	733.4123	-16.29	98	103	0	---	R.ADILFR.A
								734.4076	733.4003	733.4123	-16.28	98	103	0	---	R.ADILFR.A
								756.3906	755.3833	755.4766	-123.44	104	110	1	---	R.AAAIVRR.R
								900.4492	899.4419	899.4501	-9.13	253	260	0	---	R.FVSFTGSR.D
								900.4492	899.4419	899.4501	-9.11	253	260	0	4	R.FVSFTGSR.D
								1054.5947	1053.5874	1053.5971	-9.19	273	281	0	---	K.VNPGQIWLK.R
								1210.6782	1209.6709	1209.6982	-22.56	273	282	1	---	K.VNPGQIWLKR.V
								1298.6626	1297.6553	1297.6667	-8.73	307	319	0	---	K.SIVASAFGFSGQK.C
								1487.7115	1486.7042	1486.7238	-13.19	496	509	0	---	K.AGGPDYLALHMQAK.T + Oxidation (M)
								1510.6982	1509.6909	1509.7001	-6.06	461	472	0	---	R.EDFHVGNLYFNR.G
								1520.6930	1519.6857	1519.6943	-5.66	7	19	0	---	K.HEPFTDFSVEANK.L
								1598.7196	1597.7123	1597.7195	-4.49	78	90	0	---	K.AMQVADETFQTWR.K + Oxidation (M)
								1624.8784	1623.8711	1623.8944	-14.33	52	66	1	---	K.IVSVNPANKEELVGR.V
								1644.8210	1643.8137	1643.8267	-7.90	153	166	0	---	K.DGIPVESRPIEYNR.F
								1679.8341	1678.8268	1678.8427	-9.46	326	339	0	---	R.AVIHEDVYDHVLNR.A
								1679.8341	1678.8268	1678.8427	-9.46	326	339	0	33	R.AVIHEDVYDHVLNR.A
								1737.8434	1736.8361	1736.8383	-1.26	459	472	1	24	K.AREDFHVGNLYFNR.G
								1737.8434	1736.8361	1736.8383	-1.25	459	472	1	---	K.AREDFHVGNLYFNR.G
								1783.8877	1782.8804	1782.9048	-13.70	412	426	1	---	R.LMKEEIFGPVVAFCK.A + Oxidation (M)
								1880.9264	1879.9191	1879.9567	-20.00	29	45	0	---	K.VESYLGQDYPLIIGGEK.I
								1885.9926	1884.9853	1885.0057	-10.82	151	166	1	---	K.LKDGIPVESRPIEYNR.F
								2009.0400	2008.0327	2008.0517	-9.44	28	45	1	---	K.KVESYLGQDYPLIIGGEK.I
								2233.0808	2232.0735	2232.0256	21.5	349	370	0	---	K.VANPAVLGTNMGPVNDQAAFDK.V + 4 Deamidated (NQ)
								2321.1301	2320.1228	2320.2175	-40.82	326	345	1	---	R.AVIHEDVYDHVLNRAVELTK.E
								2367.0620	2366.0547	2366.0308	10.1	473	495	0	---	R.GCTGAIVGYQPFGGFNMSGTDSK.A + Oxidation (M)
								2889.4021	2888.3948	2888.4417	-16.21	427	453	1	---	K.AKDFDHALAIANNTEYGLTGAVITNNR.D
	**2.**	166	gi|229107970	1-pyrroline-5-carboxylate dehydrogenase [Bacillus cereus Rock1	56432											
	**3.**	166	gi|228963390	1-pyrroline-5-carboxylate dehydrogenase [Bacillus thuringiensis :	56417											
	**4.**	166	gi|30018552	1-pyrroline-5-carboxylate dehydrogenase [Bacillus cereus ATCC	56418											
	**5.**	157	gi|47569559	delta-1-pyrroline-5-carboxylate dehydrogenase, putative [Bacillu:	56404											
	**6.**	151	gi|365163390	1-pyrroline-5-carboxylate dehydrogenase [Bacillus sp. 7_6_55CI	56432											
	**7.**	151	gi|229089424	1-pyrroline-5-carboxylate dehydrogenase [Bacillus cereus Rock3	56445											
	**8.**	149	gi|228956731	1-pyrroline-5-carboxylate dehydrogenase [Bacillus thuringiensis :	56360											
	**9.**	149	gi|229165275	1-pyrroline-5-carboxylate dehydrogenase [Bacillus cereus AH62:	56421											
	**10.**	149	gi|229131298	1-pyrroline-5-carboxylate dehydrogenase [Bacillus cereus BDRD	56407											
	**11.**	149	gi|229095012	1-pyrroline-5-carboxylate dehydrogenase [Bacillus cereus Rock3	56390											
	**12.**	149	gi|229015689	1-pyrroline-5-carboxylate dehydrogenase [Bacillus cereus AH12:	56435											
	**13.**	149	gi|229009792	1-pyrroline-5-carboxylate dehydrogenase [Bacillus mycoides DSI	56407											
	**14.**	149	gi|423370432	1-pyrroline-5-carboxylate dehydrogenase [Bacillus cereus VD14:	56380											
	**15.**	149	gi|423409656	1-pyrroline-5-carboxylate dehydrogenase [Bacillus cereus BAG2	56337											

	**16.**	149	gi|423473307	1-pyrroline-5-carboxylate dehydrogenase [Bacillus cereus BAG6	56451											
	**17.**	143	gi|30260481	1-pyrroline-5-carboxylate dehydrogenase [Bacillus anthracis str.	56418											
	**18.**	143	gi|196040213	putative delta-1-pyrroline-5-carboxylate dehydrogenase [Bacillus	56446											
	**19.**	143	gi|229159449	1-pyrroline-5-carboxylate dehydrogenase [Bacillus cereus R3098	56506											
	**20.**	143	gi|384178224	1-pyrroline-5-carboxylate dehydrogenase [Bacillus thuringiensis	56432											
	**21.**	143	gi|42779419	1-pyrroline-5-carboxylate dehydrogenase [Bacillus cereus ATCC	56391											
	**22.**	143	gi|49183323	1-pyrroline-5-carboxylate dehydrogenase [Bacillus anthracis str.	56478											
	**23.**	138	gi|423613752	1-pyrroline-5-carboxylate dehydrogenase [Bacillus cereus VD10	56408											
	**24.**	137	gi|229055135	1-pyrroline-5-carboxylate dehydrogenase [Bacillus cereus AH60	56497											
	**25.**	135	gi|229083593	1-pyrroline-5-carboxylate dehydrogenase [Bacillus cereus Rock3	56365											
	**26.**	132	gi|423525901	1-pyrroline-5-carboxylate dehydrogenase [Bacillus cereus HuA4-	56412											
	**27.**	131	gi|423620090	1-pyrroline-5-carboxylate dehydrogenase [Bacillus cereus VD11	56408											
	**28.**	130	gi|163938300	1-pyrroline-5-carboxylate dehydrogenase [Bacillus weihenstepha	56425											
	**29.**	130	gi|423456090	1-pyrroline-5-carboxylate dehydrogenase [Bacillus cereus BAG5	56439											
	**30.**	127	gi|228989475	1-pyrroline-5-carboxylate dehydrogenase [Bacillus pseudomycoi	56356											
	**31.**	124	gi|75765136	Delta-1-pyrroline-5-carboxylate dehydrogenase [Bacillus thuring	40643											
	**32.**	120	gi|228995670	1-pyrroline-5-carboxylate dehydrogenase [Bacillus mycoides Roc	56371											
	**33.**	119	gi|157878102	Chain E, Complex Of Eeti-Ii With Porcine Trypsin	24841											
	**34.**	116	gi|999627	Chain B, Refined 1.8 Angstroms Resolution Crystal Structure Of	8928											
	**35.**	115	gi|229171142	1-pyrroline-5-carboxylate dehydrogenase [Bacillus cereus MM3]	56448											
	**36.**	115	gi|228906071	1-pyrroline-5-carboxylate dehydrogenase [Bacillus thuringiensis	56418											
	**37.**	114	gi|110590762	Chain A, Trypsin In Complex With Borate	24143											
	**38.**	114	gi|2914482	Chain A, Complex Of The Second Kunitz Domain Of Tissue Fac	24145											
	**39.**	114	gi|494360	Chain A, The Refined 1.6 Angstroms Resolution Crystal Structu	24143											
	**40.**	113	gi|136429	RecName: Full=Trypsin; Flags: Precursor	25078											
	**41.**	112	gi|242253868	trypsinogen precursor [Sus scrofa]	26549											
	**42.**	110	gi|1942351	Chain A, Crystal Structure Of The First Active Autolysate Form	13569											
	**43.**	108	gi|315583496	Chain A, The Bowman-Birk Type Inhibitor From Mung Bean In	24115											
	**44.**	107	gi|3318722	Chain E, Leech-Derived Tryptase InhibitorTRYPSIN COMPLE	24142											
	**45.**	106	gi|229003299	1-pyrroline-5-carboxylate dehydrogenase [Bacillus mycoides Roc	56356											
	**46.**	99	gi|423398752	1-pyrroline-5-carboxylate dehydrogenase [Bacillus cereus BAG2	56363											
**33**	**1.**	151	gi|229188566	**1-pyrroline-5-carboxylate dehydrogenase [Bacillus cereus AT**	56445	1.8e-008	23	734.4092	733.4019	733.4123	-14.10	98	103	0	---	R.ADILFR.A
								734.4092	733.4020	733.4123	-14.04	98	103	0	---	R.ADILFR.A
								900.4482	899.4409	899.4501	-10.26	253	260	0	2	R.FVSFTGSR.D
								900.4482	899.4409	899.4501	-10.24	253	260	0	---	R.FVSFTGSR.D
								1054.5770	1053.5697	1053.5971	-25.99	273	281	0	---	K.VNPGQIWLK.R
								1298.6613	1297.6540	1297.6667	-9.77	307	319	0	35	K.SIVASAFGFSGQK.C
								1298.6613	1297.6540	1297.6667	-9.73	307	319	0	---	K.SIVASAFGFSGQK.C
								1487.7065	1486.6992	1486.7238	-16.55	496	509	0	---	K.AGGPDYLALHMQAK.T + Oxidation (M)
								1510.6926	1509.6853	1509.7001	-9.77	461	472	0	---	R.EDFHVGNLYFNR.G
								1598.7163	1597.7090	1597.7195	-6.56	78	90	0	---	K.AMQVADETFQTWR.K + Oxidation (M)
								1624.8751	1623.8678	1623.8944	-16.36	52	66	1	---	K.IVSVNPANKEELVGR.V
								1644.8186	1643.8113	1643.8267	-9.36	153	166	0	---	K.DGIPVESRPIEYNR.F
								1679.8329	1678.8256	1678.8427	-10.18	326	339	0	35	R.AVIHEDVYDHVLNR.A
								1679.8329	1678.8256	1678.8427	-10.18	326	339	0	---	R.AVIHEDVYDHVLNR.A
								1737.8389	1736.8316	1736.8383	-3.84	459	472	1	---	K.AREDFHVGNLYFNR.G
								1783.8821	1782.8748	1782.9048	-16.84	412	426	1	---	R.LMKEEIFGPVVAFCK.A + Oxidation (M)
								1885.9946	1884.9873	1885.0057	-9.76	151	166	1	---	K.LKDGIPVESRPIEYNR.F
								1887.9669	1886.9596	1886.9527	3.69	395	411	0	---	K.GWFIQPTIVADVAEDAR.L
								2009.0233	2008.0160	2008.0517	-17.76	28	45	1	---	K.KVESYLGQDYPLIIGGEK.I
								2233.0640	2232.0567	2232.0256	13.9	349	370	0	---	K.VANPAVLGTNMGPVNDQAAFDK.V + 4 Deamidated (NQ)
								2249.0403	2248.0330	2248.0205	5.56	349	370	0	---	K.VANPAVLGTNMGPVNDQAAFDK.V + 4 Deamidated (NQ); Oxidation (M)
								2277.0435	2276.0362	2276.1511	-50.47	395	414	1	---	K.GWFIQPTIVADVAEDARLMK.E + Deamidated (NQ); Oxidation (M)
								2930.4119	2929.4046	2929.4458	-14.05	384	411	1	---	R.ILAGGEGDDSKGWFIQPTIVADVAEDAR.L
	**2.**	142	gi|47569559	delta-1-pyrroline-5-carboxylate dehydrogenase, putative [Bacillu	56404											
	**3.**	142	gi|229107970	1-pyrroline-5-carboxylate dehydrogenase [Bacillus cereus Rock1	56432											
	**4.**	142	gi|228963390	1-pyrroline-5-carboxylate dehydrogenase [Bacillus thuringiensis	56417											

**5.**	142	gi|30018552	1-pyrroline-5-carboxylate dehydrogenase [Bacillus cereus ATCC	56418
**6.**	140	gi|229089424	1-pyrroline-5-carboxylate dehydrogenase [Bacillus cereus Rock3	56445
**7.**	135	gi|228956731	1-pyrroline-5-carboxylate dehydrogenase [Bacillus thuringiensis :	56360
**8.**	135	gi|229095012	1-pyrroline-5-carboxylate dehydrogenase [Bacillus cereus Rock3	56390
**9.**	135	gi|423409656	1-pyrroline-5-carboxylate dehydrogenase [Bacillus cereus BAG2	56337
**10.**	135	gi|229165275	1-pyrroline-5-carboxylate dehydrogenase [Bacillus cereus AH62	56421
**11.**	135	gi|229131298	1-pyrroline-5-carboxylate dehydrogenase [Bacillus cereus BDRD	56407
**12.**	135	gi|229009792	1-pyrroline-5-carboxylate dehydrogenase [Bacillus mycoides DSI	56407
**13.**	134	gi|423613752	1-pyrroline-5-carboxylate dehydrogenase [Bacillus cereus VD10	56408
**14.**	133	gi|229055135	1-pyrroline-5-carboxylate dehydrogenase [Bacillus cereus AH60	56497
**15.**	131	gi|30260481	1-pyrroline-5-carboxylate dehydrogenase [Bacillus anthracis str.	56418
**16.**	131	gi|384178224	1-pyrroline-5-carboxylate dehydrogenase [Bacillus thuringiensis :	56432
**17.**	131	gi|365163390	1-pyrroline-5-carboxylate dehydrogenase [Bacillus sp. 7_6_55CI	56432
**18.**	131	gi|42779419	1-pyrroline-5-carboxylate dehydrogenase [Bacillus cereus ATCC	56391
**19.**	131	gi|49183323	1-pyrroline-5-carboxylate dehydrogenase [Bacillus anthracis str.	56478
**20.**	128	gi|228906071	1-pyrroline-5-carboxylate dehydrogenase [Bacillus thuringiensis	56418
**21.**	128	gi|423473307	1-pyrroline-5-carboxylate dehydrogenase [Bacillus cereus BAG6	56451
**22.**	128	gi|229015689	1-pyrroline-5-carboxylate dehydrogenase [Bacillus cereus AH12	56435
**23.**	127	gi|75765136	Delta-1-pyrroline-5-carboxylate dehydrogenase [Bacillus thuring	40643
**24.**	127	gi|423620090	1-pyrroline-5-carboxylate dehydrogenase [Bacillus cereus VD11	56408
**25.**	126	gi|163938300	1-pyrroline-5-carboxylate dehydrogenase [Bacillus weihenstephan	56425
**26.**	126	gi|423456090	1-pyrroline-5-carboxylate dehydrogenase [Bacillus cereus BAG5	56439
**27.**	124	gi|196040213	putative delta-1-pyrroline-5-carboxylate dehydrogenase [Bacillus	56446
**28.**	124	gi|229159449	1-pyrroline-5-carboxylate dehydrogenase [Bacillus cereus R3098	56506
**29.**	121	gi|423370432	1-pyrroline-5-carboxylate dehydrogenase [Bacillus cereus VD14	56380
**30.**	121	gi|229171142	1-pyrroline-5-carboxylate dehydrogenase [Bacillus cereus MM3]	56448
**31.**	118	gi|229083593	1-pyrroline-5-carboxylate dehydrogenase [Bacillus cereus Rock3	56365
**32.**	118	gi|228995670	1-pyrroline-5-carboxylate dehydrogenase [Bacillus mycoides Roc	56371
**33.**	118	gi|228989475	1-pyrroline-5-carboxylate dehydrogenase [Bacillus pseudomycoi	56356
**34.**	114	gi|423525901	1-pyrroline-5-carboxylate dehydrogenase [Bacillus cereus HuA4-	56412
**35.**	111	gi|229003299	1-pyrroline-5-carboxylate dehydrogenase [Bacillus mycoides Roc	56356
**36.**	95	gi|999627	Chain B, Refined 1.8 Angstroms Resolution Crystal Structure Of	8928
**37.**	92	gi|423398752	1-pyrroline-5-carboxylate dehydrogenase [Bacillus cereus BAG2	56363
**38.**	91	gi|242253868	trypsinogen precursor [Sus scrofa]	26549
**39.**	89	gi|1942351	Chain A, Crystal Structure Of The First Active Autolysate Form	13569
**40.**	87	gi|110590762	Chain A, Trypsin In Complex With Borate	24143
**41.**	87	gi|2914482	Chain A, Complex Of The Second Kunitz Domain Of Tissue Fac	24145
**42.**	87	gi|494360	Chain A, The Refined 1.6 Angstroms Resolution Crystal Structu	24143
**43.**	86	gi|3318722	Chain E, Leech-Derived Tryptase InhibitorTRYPSIN COMPLE	24142
**44.**	86	gi|157878102	Chain E, Complex Of Eeti-Ii With Porcine Trypsin	24841
**45.**	86	gi|136429	RecName: Full=Trypsin; Flags: Precursor	25078
**46.**	86	gi|229028149	1-pyrroline-5-carboxylate dehydrogenase [Bacillus cereus AH12	56444
