# Supplementary material for: Feces Derived Allergens of Tyrophagus putrescentiae Reared on Dried Dog Food and Evidence of the Strong Nutritional Interaction between the Mite and Bacillus cereus Producing Protease Bacillolysins and Exo-chitinases
Source: Front Physiol. 2016 Feb 24;7:53. doi: 10.3389/fphys.2016.00053 (PMC4764834; doi:10.3389/fphys.2016.00053)
Supplement: Supplementary file 2 [file Table2.PDF]

Table supplement 2 – List of proteins and details of MS/MS protein identifications in control dry dog food protein extract that served as "food" for mites

Journal name: Frontiers in Physiology – Invertebrate Physiology

Article title: Feces derived allergens of *Tyrophagus putrescentiae* reared on dried dog food and evidence of the strong nutritional interaction between the mite and *Bacillus cereus* producing protease bacillolysins and exo-chitinases

Author names: Tomas Erban*, Dagmar Rybanska, Karel Harant, Bronislava Hortova, Jan Hubert

*Corresponding author: Tomas ERBAN; Crop Research Institute; Biologically Active Substances in Crop Protection; Laboratory of Proteomics; Drnovska 507/73, Prague 6-Ruzyne; CZ-16106; Czech Republic

E-mail: arachnid@centrum.cz

A) List of proteins MS/MS identified in control dry dog food protein extract that served as "food" for mites

Spot No.	Result No.	Score	GI	Description [Taxonomy] of considered results	Mass	Expect	Queries Matched
**F01**	1.	122	gi|159793187	**alpha S1 casein, partial [Bos taurus]**	2.0e-005	13896	9
**F02**	1.	121	gi|475525322	**hypothetical protein F775_08244 [Aegilops tauschii]**	2.5e-005	15871	10
**F03**	1.	233	gi|475596183	**hypothetical protein F775_14150 [Aegilops tauschii]**	1.6e-016	19922	12
**F04**	1.	102	gi|215398470	**globulin 3 [Triticum aestivum]**	0.002	66652	12
**F05**	1.	178	gi|227808995	**dimeric alpha-amylase inhibitor [Triticum dicoccoides]**	5.0e-011	15605	13
**F06**	1.	393	gi|134034508	**monomeric alpha-amylase inhibitor [Triticum monococcum]**	1.6e-032	13658	17
**F07**	1.	322	gi|66841026	**alpha-amylase inhibitor 0.19 [Triticum aestivum]**	2.0e-022	13340	13
**F08**	1.	219	gi|54778507	**0.19 dimeric alpha-amylase inhibitor [Triticum aestivum]**	3.9e-015	13815	11
**F09**	1.	593	gi|54778507	**0.19 dimeric alpha-amylase inhibitor [Triticum aestivum]**	1.6e-052	13815	15
**F10**	1.	405	gi|66841026	**alpha-amylase inhibitor 0.19 [Triticum aestivum]**	9.9e-034	13340	13
**F11**	1.	111	gi|66841026	**alpha-amylase inhibitor 0.19 [Triticum aestivum]**	0.00025	13340	8
**F12**	1.	633	gi|66841026	**alpha-amylase inhibitor 0.19 [Triticum aestivum]**	1.6e-056	13340	15
**F13**	1.	399	gi|66841026	**alpha-amylase inhibitor 0.19 [Triticum aestivum]**	3.9e-033	13340	13
**F14**	1.	179	gi|34925030	**RecName: Full=Wheatwin-1**	3.9e-011	16024	11
**F15**	1.	177	gi|134034615	**monomeric alpha-amylase inhibitor [Triticum aestivum]**	6.3e-011	13617	11
**F16**	1.	89	gi|494377469	**regulator [Bacillus macauensis]**	0.036	25566	14
**F17**	1.	91	gi|516638230	**argininosuccinate synthase [Sinorhizobium medicae]**	0.024	44937	18

B) Details of MS/MS protein identifications in control dry dog food protein extract that served as "food" for mites

Spot No.	Result No.	Score	GI	Description [Taxonomy] - *results in bold were considered*	Mass	Expect	Queries Matched	Observed	Mr(expt)	Mr(calc)	ppm	Start	End	Miss	Ions	Peptide
**F01**	**1.**	122	gi|159793187	**alpha S1 casein, partial [Bos taurus]**	13896	2e-005	9	1267.7092	1266.7019	1266.6972	3.74	106	115	0	---	R.YLGYLEQLLR.L
								1267.7092	1266.7020	1266.6972	3.76	106	115	0	33	R.YLGYLEQLLR.L
								1337.6826	1336.6753	1336.6735	1.36	95	105	1	---	K.HIQKEDVPSER.Y
								1337.6826	1336.6753	1336.6735	1.37	95	105	1	27	K.HIQKEDVPSER.Y
								1384.7280	1383.7207	1383.7227	-1.45	38	49	0	---	R.FFVAPFPEVFGK.E
								1384.7280	1383.7208	1383.7227	-1.43	38	49	0	16	R.FFVAPFPEVFGK.E
								1759.9362	1758.9289	1758.9377	-4.99	23	37	0	---	K.HQGLPQEVLNENLLR.F
								1759.9362	1758.9289	1758.9377	-4.96	23	37	0	---	K.HQGLPQEVLNENLLR.F
								2235.2234	2234.2161	2234.2283	-5.47	19	37	1	---	K.HPIKHQGLPQEVLNENLLR.F
	**2.**	120	gi|440900746	Alpha-S1-casein, partial [Bos grunniens mutus]	24498											
	**3.**	115	gi|159793193	alpha S1 casein, partial [Bos taurus]	18728											
	**4.**	111	gi|159793197	alpha S1 casein, partial [Bos taurus]	23473											
	**5.**	111	gi|159793191	alpha S1 casein, partial [Bos taurus]	23598											
	**6.**	110	gi|225632	casein alphaS1	24477											
	**7.**	110	gi|30794348	alpha-S1-casein precursor [Bos taurus]	24570											
	**8.**	100	gi|159793209	alpha S1 casein, partial [Bos taurus]	22442											
	**9.**	92	gi|346453262	as1 casein [Bubalus bubalis]	24394											
	**10.**	92	gi|75038951	RecName: Full=Alpha-S1-casein; Flags: Precursor	24368											
	**11.**	92	gi|70905149	alpha-S1 casein [Bubalus bubalis	24440											
**F02**	**1.**	121	gi|475525322	**hypothetical protein F775_08244 [Aegilops tauschii]**	15871	2.5e-005	10	715.3736	714.3663	714.3773	-15.33	27	32	0	---	R.RPDTAR.H
								762.3860	761.3787	761.3932	-19.09	49	55	0	12	R.HPANAPR.G
								762.3860	761.3787	761.3932	-19.08	49	55	0	---	R.HPANAPR.G
								797.4325	796.4252	796.4443	-23.96	20	26	0	30	K.VPVPETR.R
								797.4325	796.4252	796.4443	-23.96	20	26	0	---	K.VPVPETR.R
								1109.5820	1108.5747	1108.5989	-21.79	37	45	1	---	R.KLEEQGLHR.L
								1205.5708	1204.5635	1204.5949	-26.03	6	15	1	---	R.VVHRDEEGHK.V
								1205.5708	1204.5635	1204.5949	-26.03	6	15	1	---	R.VVHRDEEGHK.V
								2501.0947	2500.0874	2500.1758	-35.34	72	94	0	---	K.YTWEGPEGLVDSELDPAPAAIDR.N
								2501.0947	2500.0875	2500.1758	-35.33	72	94	0	28	K.YTWEGPEGLVDSELDPAPAAIDR.N
**F03**	**1.**	233	gi|475596183	**hypothetical protein F775_14150 [Aegilops tauschii]**	19922	1.6e-016	12	756.3986	755.3913	755.3926	-1.66	186	191	0	---	R.LNQERP.-
								756.3986	755.3913	755.3926	-1.63	186	191	0	14	R.LNQERP.-
								1044.5765	1043.5692	1043.5723	-2.99	133	141	1	---	K.LRDVLSDAR.S
								1530.7498	1529.7425	1529.7620	-12.76	62	77	0	13	K.GGPAAVMQSAATLNAR.A + Oxidation (M)
								1530.7498	1529.7425	1529.7620	-12.74	62	77	0	---	K.GGPAAVMQSAATLNAR.A + Oxidation (M)
								1843.9464	1842.9391	1842.9687	-16.06	167	185	0	---	K.LDLTTTPGGVAEAVTTAAR.L
								1843.9464	1842.9391	1842.9687	-16.05	167	185	0	38	K.LDLTTTPGGVAEAVTTAAR.L
								1990.9561	1989.9488	1989.9796	-15.50	23	41	0	76	K.YGDVFDVSGELAAQPVAPR.D
								1990.9561	1989.9488	1989.9796	-15.47	23	41	0	---	K.YGDVFDVSGELAAQPVAPR.D
								2086.0710	2085.0637	2085.1066	-20.56	165	185	1	---	R.NKLDLTTTPGGVAEAVTTAAR.L
								2124.0593	2123.0520	2123.0859	-15.95	84	105	0	---	R.GQLTGPVADAGVTVTEADLPGR.R
								2124.0593	2123.0521	2123.0859	-15.94	84	105	0	39	R.GQLTGPVADAGVTVTEADLPGR.R
	**2.**	158	gi|473786240	hypothetical protein TRIUR3_05231 [Triticum urartu]	11214											
**F04**	**1.**	102	gi|215398470	**globulin 3 [Triticum aestivum]**	66652	0.002	12	701.4153	700.4080	700.4020	8.55	508	513	0	---	R.VWLAGR.N
								906.4843	905.4770	905.4607	18.1	535	541	0	---	R.EVQEVFR.A
								1045.5769	1044.5696	1044.5274	40.5	173	181	0	---	R.VAIMEVNPR.A + Deamidated (NQ); Oxidation (M)
								1045.5769	1044.5696	1044.5274	40.5	173	181	0	---	R.VAIMEVNPR.A + Deamidated (NQ); Oxidation (M)

								1822.8927	1821.8854	1821.8679	9.60	489	504	0	26	R.GSSNLQVVCFEINAER.N
								1822.8927	1821.8854	1821.8679	9.60	489	504	0	---	R.GSSNLQVVCFEINAER.N
								1906.0344	1905.0271	1905.0109	8.54	470	488	0	---	R.GSAFVVPPGHPVVEIASSR.G
								1906.0344	1905.0271	1905.0109	8.55	470	488	0	26	R.GSAFVVPPGHPVVEIASSR.G
								2225.1489	2224.1416	2224.0066	60.7	489	507	1	---	R.GSSNLQVVCFEINAERNER.V + 3 Deamidated (NQ)
								2225.1489	2224.1417	2224.0066	60.7	489	507	1	---	R.GSSNLQVVCFEINAERNER.V + 3 Deamidated (NQ)
								2426.1233	2425.1160	2425.0894	11.0	542	562	1	16	R.AKDQQDEGFVAGPEQQQEHER.G
								2426.1233	2425.1160	2425.0894	11.0	542	562	1	---	R.AKDQQDEGFVAGPEQQQEHER.G
	**2.**	102	gi|390979705	globulin-3A [Triticum aestivum]	66627											
	**3.**	87	gi|474411419	Globulin-1 S allele [Triticum urartu]	57108											
**F05**	**1.**	178	gi|227808995	**dimeric alpha-amylase inhibitor [Triticum dicoccoides]**	15605	5.00E-11	13	899.5187	898.5114	898.5488	-41.54	2	10	0	---	M.LVATPIASK.Y
								1162.6094	1161.6021	1161.6176	-13.33	107	117	0	13	K.LTAASITAVCR.L
								1162.6094	1161.6021	1161.6176	-13.31	107	117	0	---	K.LTAASITAVCR.L
								1570.7620	1569.7547	1569.7933	-24.59	43	56	0	---	K.LQCNGSQVPEAVLR.D
								1571.7590	1570.7517	1570.7773	-16.30	43	56	0	---	K.LQCNGSQVPEAVLR.D + Deamidated (NQ)
								1571.7590	1570.7518	1570.7773	-16.28	43	56	0	29	K.LQCNGSQVPEAVLR.D + Deamidated (NQ)
								1663.8019	1662.7946	1662.8287	-20.52	118	133	0	45	R.LPIVVDASGDGAYVCK.D
								1663.8019	1662.7946	1662.8287	-20.50	118	133	0	---	R.LPIVVDASGDGAYVCK.D
								1840.6979	1839.6906	1839.7338	-23.50	57	70	0	25	R.DCCQQLADISEWCR.C
								1840.6979	1839.6906	1839.7338	-23.49	57	70	0	---	R.DCCQQLADISEWCR.C
								1846.7656	1845.7583	1845.8064	-26.06	84	101	0	---	K.EHGVSEGQAGTGAFPSCR.R
								2807.2454	2806.2381	2806.4357	-70.43	107	133	1	---	K.LTAASITAVCRLPIVVDASGDGAYVCK.D
								2807.2454	2806.2381	2806.4357	-70.42	107	133	1	---	K.LTAASITAVCRLPIVVDASGDGAYVCK.D
	**2.**	169	gi|56480630	0.19 dimeric alpha-amylase inhibitor [Triticum aestivum]	13752											
	**3.**	168	gi|108597903	dimeric alpha-amylase inhibitor precursor [Triticum aestivum]	13824											
	**4.**	165	gi|65993731	dimeric alpha-amylase inhibitor [Triticum aestivum]	15606											
	**5.**	165	gi|227808851	dimeric alpha-amylase inhibitor [Triticum dicoccoides]	15658											
	**6.**	165	gi|227808853	dimeric alpha-amylase inhibitor [Triticum dicoccoides]	15618											
	**7.**	165	gi|227808867	dimeric alpha-amylase inhibitor [Triticum dicoccoides]	15578											
	**8.**	165	gi|227808912	dimeric alpha-amylase inhibitor [Triticum dicoccoides]	15638											
	**9.**	165	gi|227808928	dimeric alpha-amylase inhibitor [Triticum dicoccoides]	15605											
	**10.**	165	gi|227808974	dimeric alpha-amylase inhibitor [Triticum dicoccoides]	15592											
	**11.**	165	gi|227808999	dimeric alpha-amylase inhibitor [Triticum dicoccoides]	15584											
	**12.**	165	gi|227809150	dimeric alpha-amylase inhibitor [Triticum dicoccoides]	15578											
	**13.**	156	gi|57470930	alpha-amylase inhibitor precursor [Triticum urartu]	13849											
	**14.**	156	gi|114215888	dimeric alpha-amylase inhibitor [Aegilops sharonensis]	13926											
	**15.**	156	gi|114215810	dimeric alpha-amylase inhibitor [Triticum dicoccoides]	13995											
	**16.**	156	gi|114216010	dimeric alpha-amylase inhibitor [Aegilops longissima]	13962											
	**17.**	156	gi|114215822	dimeric alpha-amylase inhibitor [Aegilops bicornis]	13992											
	**18.**	156	gi|114215780	dimeric alpha-amylase inhibitor [Triticum dicoccoides]	13926											
	**19.**	156	gi|114215812	dimeric alpha-amylase inhibitor [Aegilops bicornis]	13962											
	**20.**	156	gi|114215958	dimeric alpha-amylase inhibitor [Aegilops searsii]	13926											
	**21.**	156	gi|114215866	dimeric alpha-amylase inhibitor [Aegilops speltoides]	13963											
	**22.**	156	gi|114215850	dimeric alpha-amylase inhibitor [Aegilops speltoides]	13843											
	**23.**	156	gi|114215834	dimeric alpha-amylase inhibitor [Aegilops speltoides]	13911											
	**24.**	156	gi|114215808	dimeric alpha-amylase inhibitor [Triticum dicoccoides]	13922											
	**25.**	156	gi|114215932	dimeric alpha-amylase inhibitor [Aegilops sharonensis]	13891											
	**26.**	156	gi|114215804	dimeric alpha-amylase inhibitor [Triticum dicoccoides]	13863											
	**27.**	156	gi|114216004	dimeric alpha-amylase inhibitor [Aegilops longissima]	13936											
	**28.**	156	gi|114215806	dimeric alpha-amylase inhibitor [Triticum dicoccoides]	13863											
	**29.**	156	gi|114215994	dimeric alpha-amylase inhibitor [Aegilops longissima]	13963											
	**30.**	156	gi|386877052	dimeric alpha-amylase inhibitor, partial [Aegilops peregrina]	13954											
	**31.**	155	gi|114215934	dimeric alpha-amylase inhibitor [Aegilops sharonensis]	13962											
	**32.**	155	gi|114215912	dimeric alpha-amylase inhibitor [Aegilops sharonensis]	13950											
	**33.**	155	gi|386877056	dimeric alpha-amylase inhibitor, partial [Aegilops longissima]	14792											
	**34.**	155	gi|386877058	dimeric alpha-amylase inhibitor, partial [Aegilops longissima]	14719											
	**35.**	154	gi|227808970	dimeric alpha-amylase inhibitor [Triticum dicoccoides]	15576											

	**36.**	154	gi|227809013	dimeric alpha-amylase inhibitor [Triticum dicoccoides]	15607											
	**37.**	154	gi|227809076	dimeric alpha-amylase inhibitor [Triticum dicoccoides]	15686											
	**38.**	154	gi|227809078	dimeric alpha-amylase inhibitor [Triticum dicoccoides]	15722											
	**39.**	154	gi|227809102	dimeric alpha-amylase inhibitor [Triticum dicoccoides]	15678											
	**40.**	154	gi|227809108	dimeric alpha-amylase inhibitor [Triticum dicoccoides]	15751											
	**41.**	154	gi|227809119	dimeric alpha-amylase inhibitor [Triticum dicoccoides]	15678											
	**42.**	154	gi|227809228	dimeric alpha-amylase inhibitor [Triticum dicoccoides]	15703											
	**43.**	154	gi|227809230	dimeric alpha-amylase inhibitor [Triticum dicoccoides]	15782											
	**44.**	154	gi|227809238	dimeric alpha-amylase inhibitor [Triticum dicoccoides]	15820											
	**45.**	154	gi|227809242	dimeric alpha-amylase inhibitor [Triticum dicoccoides]	15756											
	**46.**	154	gi|227809250	dimeric alpha-amylase inhibitor [Triticum dicoccoides]	15694											
	**47.**	154	gi|227809252	dimeric alpha-amylase inhibitor [Triticum dicoccoides]	15716											
	**48.**	154	gi|227809254	dimeric alpha-amylase inhibitor [Triticum dicoccoides]	15753											
	**49.**	154	gi|227809328	dimeric alpha-amylase inhibitor [Eremopyrum bonaepartis]	15741											
	**50.**	154	gi|227809396	dimeric alpha-amylase inhibitor [Aegilops comosa]	15660											
**F06**	**1.**	393	gi|134034508	**monomeric alpha-amylase inhibitor [Triticum monococcum]**	13658	1.6e-032	17	830.4344	829.4271	829.4116	18.7	77	83	0	31	K.EVLPGCR.K
								830.4344	829.4271	829.4116	18.7	77	83	0	---	K.EVLPGCR.K
								863.4590	862.4517	862.4331	21.6	15	22	0	---	K.VSALTGCR.A
								1037.4613	1036.4540	1036.4794	-24.45	56	64	0	---	R.CGNLSSMLR.S
								1038.4801	1037.4728	1037.4634	9.10	56	64	0	12	R.CGNLSSMLR.S + Deamidated (NQ)
								1038.4801	1037.4728	1037.4634	9.10	56	64	0	---	R.CGNLSSMLR.S + Deamidated (NQ)
								1050.5760	1049.5687	1049.5506	17.3	65	73	0	---	R.SVYQELGVR.E
								1050.5760	1049.5688	1049.5506	17.4	65	73	0	42	R.SVYQELGVR.E
								1051.5759	1050.5686	1050.5458	21.7	100	109	0	---	K.VPIPNPSGDR.A
								1054.5270	1053.5197	1053.4583	58.3	56	64	0	---	R.CGNLSSMLR.S + Deamidated (NQ); Oxidation (M)
								1174.6267	1173.6194	1173.6064	11.1	89	99	0	---	K.LTAASVPEVCK.V
								1555.8413	1554.8340	1554.8188	9.78	27	40	0	---	K.LQCVGSQVPEAVLR.D
								1555.8413	1554.8340	1554.8188	9.79	27	40	0	69	K.LQCVGSQVPEAVLR.D
								1611.7034	1610.6961	1610.6824	8.51	1	14	0	71	-.SGPWSWCDPATGYK.V
								1611.7034	1610.6961	1610.6824	8.53	1	14	0	---	-.SGPWSWCDPATGYK.V
								1981.8053	1980.7980	1980.7877	5.23	41	55	0	75	R.DCCQQLADINNEWCR.C
								1981.8053	1980.7980	1980.7877	5.23	41	55	0	---	R.DCCQQLADINNEWCR.C
	**2.**	391	gi|134034521	monomeric alpha-amylase inhibitor [Triticum monococcum]	13717											
	**3.**	391	gi|134034506	monomeric alpha-amylase inhibitor [Triticum monococcum]	13659											
	**4.**	378	gi|134034615	monomeric alpha-amylase inhibitor [Triticum aestivum]	13617											
	**5.**	377	gi|134034577	monomeric alpha-amylase inhibitor [Triticum aestivum]	13668											
	**6.**	365	gi|134034537	monomeric alpha-amylase inhibitor [Triticum monococcum]	13699											
	**7.**	364	gi|134034613	monomeric alpha-amylase inhibitor [Triticum aestivum]	13638											
	**8.**	334	gi|134034565	monomeric alpha-amylase inhibitor [Aegilops tauschii]	13749											
	**9.**	333	gi|223520	inhibitor,alpha amylase	13961											
	**10.**	331	gi|134034545	monomeric alpha-amylase inhibitor [Aegilops bicornis]	13704											
	**11.**	320	gi|134034539	monomeric alpha-amylase inhibitor [Triticum monococcum]	13643											
	**12.**	299	gi|2894148	monomeric alpha-amylase inhibitor [Triticum aestivum]	17187											
	**13.**	299	gi|229614977	monomeric alpha-amylase inhibitor [Triticum dicoccoides]	17173											
	**14.**	299	gi|229615063	monomeric alpha-amylase inhibitor [Triticum dicoccoides]	17203											
	**15.**	299	gi|229615203	monomeric alpha-amylase inhibitor [Triticum dicoccoides]	17143											
	**16.**	299	gi|229615474	monomeric alpha-amylase inhibitor [Triticum dicoccoides]	17206											
	**17.**	299	gi|229615476	monomeric alpha-amylase inhibitor [Triticum dicoccoides]	17177											
	**18.**	299	gi|229615540	monomeric alpha-amylase inhibitor [Triticum dicoccoides]	17233											
	**19.**	299	gi|229615658	monomeric alpha-amylase inhibitor [Triticum dicoccoides]	17173											
	**20.**	299	gi|229615664	monomeric alpha-amylase inhibitor [Triticum dicoccoides]	17201											
	**21.**	298	gi|110282974	RecName: Full=Alpha-amylase inhibitor 0.28; AltName: Full=C	17358											
	**22.**	296	gi|229615119	monomeric alpha-amylase inhibitor [Triticum dicoccoides]	17272											
	**23.**	289	gi|134034641	monomeric alpha-amylase inhibitor [Triticum aestivum]	13587											
	**24.**	288	gi|227809342	dimeric alpha-amylase inhibitor [Heteranthelium piliferum]	15595											
	**25.**	287	gi|229614961	monomeric alpha-amylase inhibitor [Triticum dicoccoides]	17104											
	**26.**	287	gi|229614963	monomeric alpha-amylase inhibitor [Triticum dicoccoides]	17074											
	**27.**	287	gi|229614993	monomeric alpha-amylase inhibitor [Triticum dicoccoides]	17102											

	**28.**	287	gi|229615039	monomeric alpha-amylase inhibitor [Triticum dicoccoides]	17118											
	**29.**	287	gi|229615085	monomeric alpha-amylase inhibitor [Triticum dicoccoides]	17106											
	**30.**	287	gi|229615107	monomeric alpha-amylase inhibitor [Triticum dicoccoides]	17074											
	**31.**	287	gi|229615123	monomeric alpha-amylase inhibitor [Triticum dicoccoides]	17044											
	**32.**	287	gi|229615147	monomeric alpha-amylase inhibitor [Triticum dicoccoides]	17030											
	**33.**	287	gi|229615171	monomeric alpha-amylase inhibitor [Triticum dicoccoides]	17056											
	**34.**	287	gi|229615179	monomeric alpha-amylase inhibitor [Triticum dicoccoides]	17130											
	**35.**	287	gi|229615183	monomeric alpha-amylase inhibitor [Triticum dicoccoides]	17130											
	**36.**	287	gi|229615253	monomeric alpha-amylase inhibitor [Triticum dicoccoides]	17003											
	**37.**	287	gi|229615259	monomeric alpha-amylase inhibitor [Triticum dicoccoides]	17132											
	**38.**	287	gi|229615322	monomeric alpha-amylase inhibitor [Triticum dicoccoides]	17054											
	**39.**	287	gi|229615336	monomeric alpha-amylase inhibitor [Triticum dicoccoides]	17108											
	**40.**	287	gi|229615380	monomeric alpha-amylase inhibitor [Triticum dicoccoides]	17103											
	**41.**	287	gi|229615470	monomeric alpha-amylase inhibitor [Triticum dicoccoides]	17072											
	**42.**	287	gi|229615542	monomeric alpha-amylase inhibitor [Triticum dicoccoides]	17044											
	**43.**	287	gi|229615590	monomeric alpha-amylase inhibitor [Triticum dicoccoides]	17118											
	**44.**	287	gi|229615618	monomeric alpha-amylase inhibitor, partial [Triticum dicoccoides	17230											
	**45.**	287	gi|229615290	monomeric alpha-amylase inhibitor [Triticum dicoccoides]	17162											
	**46.**	287	gi|229615199	monomeric alpha-amylase inhibitor [Triticum dicoccoides]	17231											
	**47.**	286	gi|229615151	monomeric alpha-amylase inhibitor [Triticum dicoccoides]	17176											
	**48.**	286	gi|229615398	monomeric alpha-amylase inhibitor [Triticum dicoccoides]	17070											
	**49.**	285	gi|229615173	monomeric alpha-amylase inhibitor [Triticum dicoccoides]	17130											
	**50.**	285	gi|229614991	monomeric alpha-amylase inhibitor [Triticum dicoccoides]	17072											
**F07**	**1.**	322	gi|66841026	**alpha-amylase inhibitor 0.19 [Triticum aestivum]**	13340	2e-025	13	1162.6298	1161.6225	1161.6176	4.22	85	95	0	16	K.LTAASITAVCR.L
								1162.6298	1161.6225	1161.6176	4.25	85	95	0	---	K.LTAASITAVCR.L
								1570.7950	1569.7877	1569.7933	-3.56	21	34	0	---	R.LQCNGSQVPEAVLR.D
								1570.7950	1569.7878	1569.7933	-3.54	21	34	0	44	R.LQCNGSQVPEAVLR.D
								1571.7892	1570.7819	1570.7773	2.93	21	34	0	---	R.LQCNGSQVPEAVLR.D + Deamidated (NQ)
								1612.7430	1611.7357	1611.7390	-2.02	62	77	0	---	K.EHGAQEGQAGTGAFPR.C
								1612.7430	1611.7358	1611.7390	-2.00	62	77	0	96	K.EHGAQEGQAGTGAFPR.C
								1663.8334	1662.8261	1662.8287	-1.58	96	111	0	77	R.LPIVVDASGDGAYVCK.D
								1663.8334	1662.8261	1662.8287	-1.56	96	111	0	---	R.LPIVVDASGDGAYVCK.D
								1862.7721	1861.7648	1861.7658	-0.53	35	48	0	29	R.DCCQQLAHISEWCR.C
								1862.7721	1861.7648	1861.7658	-0.53	35	48	0	---	R.DCCQQLAHISEWCR.C
								2807.3357	2806.3284	2806.4357	-38.24	85	111	1	---	K.LTAASITAVCRLPIVVDASGDGAYVCK.D
								2807.3357	2806.3284	2806.4357	-38.24	85	111	1	---	K.LTAASITAVCRLPIVVDASGDGAYVCK.D
	**2.**	321	gi|123963	RecName: Full=Alpha-amylase inhibitor 0.19; AltName: Full=0.	13899											
	**3.**	321	gi|108597921	dimeric alpha-amylase inhibitor precursor, partial [Triticum aesti	14030											
	**4.**	320	gi|386877048	dimeric alpha-amylase inhibitor, partial [Aegilops kotschyi]	14199											
	**5.**	320	gi|386877062	dimeric alpha-amylase inhibitor, partial [Aegilops geniculata]	14543											
	**6.**	320	gi|386877046	dimeric alpha-amylase inhibitor, partial [Aegilops tauschii]	14670											
	**7.**	319	gi|386877044	dimeric alpha-amylase inhibitor, partial [Aegilops tauschii]	14805											
	**8.**	319	gi|386877050	dimeric alpha-amylase inhibitor, partial [Aegilops tauschii]	14826											
	**9.**	319	gi|386877060	dimeric alpha-amylase inhibitor, partial [Aegilops longissima]	14954											
	**10.**	318	gi|65993781	dimeric alpha-amylase inhibitor [Triticum aestivum]	15688											
	**11.**	318	gi|227809005	dimeric alpha-amylase inhibitor [Triticum dicoccoides]	15730											
	**12.**	318	gi|227809300	dimeric alpha-amylase inhibitor [Aegilops uniaristata]	15758											
	**13.**	318	gi|227809314	dimeric alpha-amylase inhibitor [Aegilops tauschii]	15762											
	**14.**	318	gi|227809364	dimeric alpha-amylase inhibitor [Aegilops tauschii]	15742											
	**15.**	318	gi|227809384	dimeric alpha-amylase inhibitor [Aegilops tauschii]	15791											
	**16.**	318	gi|386877038	dimeric alpha-amylase inhibitor [Triticum aestivum]	15702											
	**17.**	317	gi|54778521	0.19 dimeric alpha-amylase inhibitor [Aegilops tauschii]	13881											
	**18.**	316	gi|475613321	Alpha-amylase inhibitor 0.19 [Aegilops tauschii]	17198											
	**19.**	278	gi|54778515	0.19 dimeric alpha-amylase inhibitor [Aegilops tauschii]	13869											
	**20.**	278	gi|114215934	dimeric alpha-amylase inhibitor [Aegilops sharonensis]	13962											
	**21.**	278	gi|114215808	dimeric alpha-amylase inhibitor [Triticum dicoccoides]	13922											
	**22.**	278	gi|114215932	dimeric alpha-amylase inhibitor [Aegilops sharonensis]	13891											
	**23.**	278	gi|114215804	dimeric alpha-amylase inhibitor [Triticum dicoccoides]	13863											

	**24.**	278	gi|114215806	dimeric alpha-amylase inhibitor [Triticum dicoccoides]	13863											
	**25.**	277	gi|386877056	dimeric alpha-amylase inhibitor, partial [Aegilops longissima]	14792											
	**26.**	277	gi|386877058	dimeric alpha-amylase inhibitor, partial [Aegilops longissima]	14719											
	**27.**	276	gi|65993829	dimeric alpha-amylase inhibitor [Triticum aestivum]	15722											
	**28.**	276	gi|227809078	dimeric alpha-amylase inhibitor [Triticum dicoccoides]	15722											
	**29.**	276	gi|227809102	dimeric alpha-amylase inhibitor [Triticum dicoccoides]	15678											
	**30.**	276	gi|227809250	dimeric alpha-amylase inhibitor [Triticum dicoccoides]	15694											
	**31.**	276	gi|227809252	dimeric alpha-amylase inhibitor [Triticum dicoccoides]	15716											
	**32.**	276	gi|227809254	dimeric alpha-amylase inhibitor [Triticum dicoccoides]	15753											
	**33.**	276	gi|227809316	dimeric alpha-amylase inhibitor [Aegilops tauschii]	15704											
	**34.**	276	gi|227809370	dimeric alpha-amylase inhibitor [Aegilops tauschii]	15756											
	**35.**	276	gi|227809431	dimeric alpha-amylase inhibitor [Hordeum vulgare]	15605											
	**36.**	262	gi|54778503	0.19 dimeric alpha-amylase inhibitor [Triticum aestivum]	13827											
	**37.**	260	gi|65993925	dimeric alpha-amylase inhibitor [Triticum aestivum]	15702											
	**38.**	260	gi|227809156	dimeric alpha-amylase inhibitor [Triticum dicoccoides]	15716											
	**39.**	260	gi|227809366	dimeric alpha-amylase inhibitor [Aegilops tauschii]	15714											
	**40.**	238	gi|114216000	dimeric alpha-amylase inhibitor [Aegilops longissima]	13831											
	**41.**	221	gi|114215840	dimeric alpha-amylase inhibitor [Aegilops speltoides]	13933											
	**42.**	221	gi|386877054	dimeric alpha-amylase inhibitor, partial [Aegilops peregrina]	14146											
	**43.**	217	gi|386877068	dimeric alpha-amylase inhibitor, partial [Triticum aestivum]	14416											
	**44.**	217	gi|452055914	dimeric alpha-amylase inhibitor, partial [Aegilops geniculata]	14748											
	**45.**	212	gi|114215880	dimeric alpha-amylase inhibitor [Aegilops speltoides]	13964											
	**46.**	212	gi|114215868	dimeric alpha-amylase inhibitor [Aegilops speltoides]	13929											
	**47.**	209	gi|227809415	dimeric alpha-amylase inhibitor [Henrardia persica]	15658											
	**48.**	198	gi|227809348	dimeric alpha-amylase inhibitor [Secale cereale]	16037											
	**49.**	198	gi|227809350	dimeric alpha-amylase inhibitor [Secale cereale]	16058											
	**50.**	189	gi|255988225	dimeric alpha-amylase inhibitor [Triticum aestivum]	15666											
**F08**	**1.**	219	gi|54778507	**0.19 dimeric alpha-amylase inhibitor [Triticum aestivum]**	13815	3.9e-015	11	1306.7683	1305.7610	1305.7591	1.47	14	25	0	---	K.VPALPGCRPVLK.L
								1306.7683	1305.7610	1305.7591	1.47	14	25	0	16	K.VPALPGCRPVLK.L
								1570.7964	1569.7891	1569.7933	-2.68	26	39	0	27	K.LQCNGSQVPEAVLR.E
								1570.7964	1569.7891	1569.7933	-2.67	26	39	0	---	K.LQCNGSQVPEAVLR.E
								1571.7931	1570.7858	1570.7773	5.41	26	39	0	---	K.LQCNGSQVPEAVLR.E + Deamidated (NQ)
								1677.8475	1676.8402	1676.8444	-2.47	101	116	0	---	K.LPIVIDASGDGAYVCK.D
								1677.8475	1676.8403	1676.8444	-2.45	101	116	0	66	K.LPIVIDASGDGAYVCK.D
								1854.7482	1853.7409	1853.7495	-4.63	40	53	0	27	R.ECCQQLADISEWCR.C
								1854.7482	1853.7409	1853.7495	-4.62	40	53	0	---	R.ECCQQLADISEWCR.C
								1887.8357	1886.8284	1886.8330	-2.42	67	84	0	42	K.EHGVQEGQAGTGAFPSCR.R
								1887.8357	1886.8284	1886.8330	-2.41	67	84	0	---	K.EHGVQEGQAGTGAFPSCR.R
	**2.**	219	gi|54778511	0.19 dimeric alpha-amylase inhibitor [Triticum aestivum]	13757											
	**3.**	217	gi|65993852	dimeric alpha-amylase inhibitor [Triticum aestivum]	15558											
	**4.**	217	gi|65993807	dimeric alpha-amylase inhibitor [Triticum aestivum]	15588											
	**5.**	217	gi|65993941	dimeric alpha-amylase inhibitor [Triticum aestivum]	15631											
	**6.**	180	gi|114215762	dimeric alpha-amylase inhibitor [Triticum dicoccoides]	13743											
	**7.**	178	gi|227808839	dimeric alpha-amylase inhibitor [Triticum dicoccoides]	15588											
	**8.**	178	gi|227808857	dimeric alpha-amylase inhibitor [Triticum dicoccoides]	15562											
	**9.**	178	gi|227808876	dimeric alpha-amylase inhibitor [Triticum dicoccoides]	15588											
	**10.**	178	gi|227808906	dimeric alpha-amylase inhibitor [Triticum dicoccoides]	15574											
	**11.**	178	gi|227809001	dimeric alpha-amylase inhibitor [Triticum dicoccoides]	15573											
	**12.**	178	gi|227809055	dimeric alpha-amylase inhibitor [Triticum dicoccoides]	15558											
	**13.**	178	gi|227809172	dimeric alpha-amylase inhibitor [Triticum dicoccoides]	15548											
	**14.**	178	gi|227809192	dimeric alpha-amylase inhibitor [Triticum dicoccoides]	15604											
	**15.**	178	gi|227809294	dimeric alpha-amylase inhibitor [Triticum timopheevii subsp. arn	15588											
	**16.**	154	gi|114215876	dimeric alpha-amylase inhibitor [Aegilops speltoides]	13998											
	**17.**	154	gi|114215874	dimeric alpha-amylase inhibitor [Aegilops speltoides]	13981											
	**18.**	154	gi|386877064	dimeric alpha-amylase inhibitor, partial [Triticum durum]	14503											
	**19.**	153	gi|227808997	dimeric alpha-amylase inhibitor [Triticum dicoccoides]	15575											
	**20.**	153	gi|227809204	dimeric alpha-amylase inhibitor [Triticum dicoccoides]	15564											
	**21.**	142	gi|227809045	dimeric alpha-amylase inhibitor [Triticum dicoccoides]	15558											

	**22.**	142	gi|227809222	dimeric alpha-amylase inhibitor [Triticum dicoccoides]	15602											
	**23.**	140	gi|65993756	dimeric alpha-amylase inhibitor [Triticum aestivum]	15821											
	**24.**	140	gi|227809457	dimeric alpha-amylase inhibitor [Thinopyrum bessarabicum]	15628											
	**25.**	128	gi|114215782	dimeric alpha-amylase inhibitor [Triticum dicoccoides]	13773											
	**26.**	128	gi|114215844	dimeric alpha-amylase inhibitor [Aegilops speltoides]	13847											
	**27.**	127	gi|227809110	dimeric alpha-amylase inhibitor [Triticum dicoccoides]	15604											
	**28.**	127	gi|255988225	dimeric alpha-amylase inhibitor [Triticum aestivum]	15666											
	**29.**	111	gi|227809324	dimeric alpha-amylase inhibitor [Eremopyrum bonaepartis]	15637											
	**30.**	111	gi|227809330	dimeric alpha-amylase inhibitor [Eremopyrum bonaepartis]	15680											
	**31.**	104	gi|114215784	dimeric alpha-amylase inhibitor [Triticum dicoccoides]	13771											
	**32.**	104	gi|57470938	alpha-amylase inhibitor precursor [Aegilops speltoides]	13871											
	**33.**	104	gi|57470935	alpha-amylase inhibitor precursor [Aegilops speltoides]	13956											
	**34.**	104	gi|114215936	dimeric alpha-amylase inhibitor [Aegilops searsii]	13950											
	**35.**	104	gi|114215966	dimeric alpha-amylase inhibitor [Aegilops searsii]	13980											
	**36.**	104	gi|114215980	dimeric alpha-amylase inhibitor [Aegilops searsii]	13963											
	**37.**	104	gi|114215952	dimeric alpha-amylase inhibitor [Aegilops searsii]	13908											
	**38.**	104	gi|452055908	dimeric alpha-amylase inhibitor, partial [Triticum urartu]	14386											
	**39.**	104	gi|386877068	dimeric alpha-amylase inhibitor, partial [Triticum aestivum]	14416											
	**40.**	104	gi|452055910	dimeric alpha-amylase inhibitor, partial [Triticum urartu]	14572											
	**41.**	103	gi|452055914	dimeric alpha-amylase inhibitor, partial [Aegilops geniculata]	14748											
	**42.**	103	gi|386877040	dimeric alpha-amylase inhibitor, partial [Triticum aestivum]	15014											
	**43.**	103	gi|227809426	dimeric alpha-amylase inhibitor [Agropyron desertorum]	15752											
	**44.**	103	gi|227809112	dimeric alpha-amylase inhibitor [Triticum dicoccoides]	15602											
	**45.**	103	gi|227809278	dimeric alpha-amylase inhibitor [Triticum timopheevii subsp. arn	15588											
	**46.**	96	gi|227809286	dimeric alpha-amylase inhibitor [Triticum timopheevii subsp. arn	15513											
	**47.**	96	gi|227809290	dimeric alpha-amylase inhibitor [Triticum timopheevii subsp. arn	15571											
**F09**	**1.**	593	gi|54778507	**0.19 dimeric alpha-amylase inhibitor [Triticum aestivum]**	13815	1.6e-052	15	1306.7712	1305.7639	1305.7591	3.69	14	25	0	---	K.VPALPGCRPVLK.L
								1306.7712	1305.7640	1305.7591	3.72	14	25	0	39	K.VPALPGCRPVLK.L
								1538.6600	1537.6527	1537.6615	-5.70	54	66	0	---	R.CGALYSMLDSMYK.E
								1538.6600	1537.6528	1537.6615	-5.68	54	66	0	38	R.CGALYSMLDSMYK.E
								1563.6741	1562.6668	1562.6686	-1.16	1	13	0	---	-.SGPWMCYPGYAFK.V
								1570.7974	1569.7901	1569.7933	-2.04	26	39	0	---	K.LQCNGSQVPEAVLR.E
								1571.7886	1570.7813	1570.7773	2.52	26	39	0	70	K.LQCNGSQVPEAVLR.E + Deamidated (NQ)
								1571.7886	1570.7813	1570.7773	2.54	26	39	0	---	K.LQCNGSQVPEAVLR.E + Deamidated (NQ)
								1579.6726	1578.6653	1578.6636	1.12	1	13	0	---	-.SGPWMCYPGYAFK.V + Oxidation (M)
								1677.8438	1676.8365	1676.8444	-4.70	101	116	0	149	K.LPIVIDASGDGAYVCK.D
								1677.8438	1676.8365	1676.8444	-4.67	101	116	0	---	K.LPIVIDASGDGAYVCK.D
								1854.7489	1853.7416	1853.7495	-4.24	40	53	0	111	R.ECCQQLADISEWCR.C
								1854.7489	1853.7416	1853.7495	-4.24	40	53	0	---	R.ECCQQLADISEWCR.C
								1887.8331	1886.8258	1886.8330	-3.79	67	84	0	---	K.EHGVQEGQAGTGAFPSCR.R
								1887.8331	1886.8259	1886.8330	-3.78	67	84	0	117	K.EHGVQEGQAGTGAFPSCR.R
	**2.**	593	gi|54778511	0.19 dimeric alpha-amylase inhibitor [Triticum aestivum]	13757											
	**3.**	576	gi|65993807	dimeric alpha-amylase inhibitor [Triticum aestivum]	15588											
	**4.**	576	gi|65993941	dimeric alpha-amylase inhibitor [Triticum aestivum]	15631											
	**5.**	526	gi|65993852	dimeric alpha-amylase inhibitor [Triticum aestivum]	15558											
	**6.**	481	gi|114215762	dimeric alpha-amylase inhibitor [Triticum dicoccoides]	13743											
	**7.**	464	gi|227808839	dimeric alpha-amylase inhibitor [Triticum dicoccoides]	15588											
	**8.**	464	gi|227808857	dimeric alpha-amylase inhibitor [Triticum dicoccoides]	15562											
	**9.**	464	gi|227808876	dimeric alpha-amylase inhibitor [Triticum dicoccoides]	15588											
	**10.**	464	gi|227808906	dimeric alpha-amylase inhibitor [Triticum dicoccoides]	15574											
	**11.**	464	gi|227809001	dimeric alpha-amylase inhibitor [Triticum dicoccoides]	15573											
	**12.**	464	gi|227809055	dimeric alpha-amylase inhibitor [Triticum dicoccoides]	15558											
	**13.**	464	gi|227809172	dimeric alpha-amylase inhibitor [Triticum dicoccoides]	15548											
	**14.**	464	gi|227809192	dimeric alpha-amylase inhibitor [Triticum dicoccoides]	15604											
	**15.**	464	gi|227809294	dimeric alpha-amylase inhibitor [Triticum timopheevii subsp. arn	15588											
	**16.**	415	gi|65993756	dimeric alpha-amylase inhibitor [Triticum aestivum]	15821											
	**17.**	415	gi|227809457	dimeric alpha-amylase inhibitor [Thinopyrum bessarabicum]	15628											
	**18.**	414	gi|386877064	dimeric alpha-amylase inhibitor, partial [Triticum durum]	14503											

	**19.**	413	gi|227808997	dimeric alpha-amylase inhibitor [Triticum dicoccoides]	15575											
	**20.**	413	gi|227809204	dimeric alpha-amylase inhibitor [Triticum dicoccoides]	15564											
	**21.**	392	gi|227809045	dimeric alpha-amylase inhibitor [Triticum dicoccoides]	15558											
	**22.**	367	gi|114215876	dimeric alpha-amylase inhibitor [Aegilops speltoides]	13998											
	**23.**	367	gi|114215874	dimeric alpha-amylase inhibitor [Aegilops speltoides]	13981											
	**24.**	350	gi|114215782	dimeric alpha-amylase inhibitor [Triticum dicoccoides]	13773											
	**25.**	350	gi|114215844	dimeric alpha-amylase inhibitor [Aegilops speltoides]	13847											
	**26.**	344	gi|227809222	dimeric alpha-amylase inhibitor [Triticum dicoccoides]	15602											
	**27.**	335	gi|227809110	dimeric alpha-amylase inhibitor [Triticum dicoccoides]	15604											
	**28.**	324	gi|255988225	dimeric alpha-amylase inhibitor [Triticum aestivum]	15666											
	**29.**	318	gi|114215784	dimeric alpha-amylase inhibitor [Triticum dicoccoides]	13771											
	**30.**	303	gi|227809112	dimeric alpha-amylase inhibitor [Triticum dicoccoides]	15602											
	**31.**	303	gi|227809278	dimeric alpha-amylase inhibitor [Triticum timopheevii subsp. arn	15588											
	**32.**	298	gi|57470938	alpha-amylase inhibitor precursor [Aegilops speltoides]	13871											
	**33.**	298	gi|57470935	alpha-amylase inhibitor precursor [Aegilops speltoides]	13956											
	**34.**	292	gi|227809324	dimeric alpha-amylase inhibitor [Eremopyrum bonaepartis]	15637											
	**35.**	292	gi|227809330	dimeric alpha-amylase inhibitor [Eremopyrum bonaepartis]	15680											
	**36.**	287	gi|114215936	dimeric alpha-amylase inhibitor [Aegilops searsii]	13950											
	**37.**	287	gi|114215966	dimeric alpha-amylase inhibitor [Aegilops searsii]	13980											
	**38.**	287	gi|114215980	dimeric alpha-amylase inhibitor [Aegilops searsii]	13963											
	**39.**	287	gi|114215952	dimeric alpha-amylase inhibitor [Aegilops searsii]	13908											
	**40.**	286	gi|452055908	dimeric alpha-amylase inhibitor, partial [Triticum urartu]	14386											
	**41.**	286	gi|452055910	dimeric alpha-amylase inhibitor, partial [Triticum urartu]	14572											
	**42.**	285	gi|386877040	dimeric alpha-amylase inhibitor, partial [Triticum aestivum]	15014											
	**43.**	276	gi|386877068	dimeric alpha-amylase inhibitor, partial [Triticum aestivum]	14416											
	**44.**	276	gi|452055914	dimeric alpha-amylase inhibitor, partial [Aegilops geniculata]	14748											
	**45.**	264	gi|227809286	dimeric alpha-amylase inhibitor [Triticum timopheevii subsp. arn	15513											
	**46.**	264	gi|227809290	dimeric alpha-amylase inhibitor [Triticum timopheevii subsp. arn	15571											
	**47.**	253	gi|227809021	dimeric alpha-amylase inhibitor [Triticum dicoccoides]	15805											
	**48.**	238	gi|227809426	dimeric alpha-amylase inhibitor [Agropyron desertorum]	15752											
	**49.**	221	gi|227809282	dimeric alpha-amylase inhibitor [Triticum timopheevii subsp. arn	15606											
	**50.**	206	gi|114215872	dimeric alpha-amylase inhibitor [Aegilops speltoides]	13792											
**F10**	**1.**	405	gi|66841026	**alpha-amylase inhibitor 0.19 [Triticum aestivum]**	13340	9.9e-034	13	1162.6239	1161.6166	1161.6176	-0.83	85	95	0	29	K.LTAASITAVCR.L
								1162.6239	1161.6166	1161.6176	-0.83	85	95	0	---	K.LTAASITAVCR.L
								1538.6528	1537.6455	1537.6615	-10.38	49	61	0	---	R.CGALYSMLDSMYK.E
								1570.7949	1569.7876	1569.7933	-3.63	21	34	0	---	R.LQCNGSQVPEAVLR.D
								1571.7819	1570.7746	1570.7773	-1.75	21	34	0	81	R.LQCNGSQVPEAVLR.D + Deamidated (NQ)
								1571.7819	1570.7746	1570.7773	-1.72	21	34	0	---	R.LQCNGSQVPEAVLR.D + Deamidated (NQ)
								1612.7393	1611.7320	1611.7390	-4.34	62	77	0	92	K.EHGAQEGQAGTGAFPR.C
								1612.7393	1611.7320	1611.7390	-4.32	62	77	0	---	K.EHGAQEGQAGTGAFPR.C
								1663.8264	1662.8191	1662.8287	-5.77	96	111	0	---	R.LPIVVDASGDGAYVCK.D
								1663.8264	1662.8191	1662.8287	-5.76	96	111	0	114	R.LPIVVDASGDGAYVCK.D
								1862.7626	1861.7553	1861.7658	-5.65	35	48	0	25	R.DCCQQLAHISEWCR.C
								1862.7626	1861.7553	1861.7658	-5.63	35	48	0	---	R.DCCQQLAHISEWCR.C
								2807.3271	2806.3198	2806.4357	-41.31	85	111	1	---	K.LTAASITAVCRLPIVVDASGDGAYVCK.D
	**2.**	404	gi|123963	RecName: Full=Alpha-amylase inhibitor 0.19; AltName: Full=0.	13899											
	**3.**	404	gi|108597921	dimeric alpha-amylase inhibitor precursor, partial [Triticum aesti	14030											
	**4.**	403	gi|386877048	dimeric alpha-amylase inhibitor, partial [Aegilops kotschyi]	14199											
	**5.**	403	gi|386877062	dimeric alpha-amylase inhibitor, partial [Aegilops geniculata]	14543											
	**6.**	403	gi|386877046	dimeric alpha-amylase inhibitor, partial [Aegilops tauschii]	14670											
	**7.**	402	gi|386877044	dimeric alpha-amylase inhibitor, partial [Aegilops tauschii]	14805											
	**8.**	402	gi|386877050	dimeric alpha-amylase inhibitor, partial [Aegilops tauschii]	14826											
	**9.**	402	gi|386877060	dimeric alpha-amylase inhibitor, partial [Aegilops longissima]	14954											
	**10.**	401	gi|65993781	dimeric alpha-amylase inhibitor [Triticum aestivum]	15688											
	**11.**	401	gi|227809005	dimeric alpha-amylase inhibitor [Triticum dicoccoides]	15730											
	**12.**	401	gi|227809314	dimeric alpha-amylase inhibitor [Aegilops tauschii]	15762											
	**13.**	401	gi|227809364	dimeric alpha-amylase inhibitor [Aegilops tauschii]	15742											
	**14.**	401	gi|386877038	dimeric alpha-amylase inhibitor [Triticum aestivum]	15702											

	**15.**	400	gi|54778521	0.19 dimeric alpha-amylase inhibitor [Aegilops tauschii]	13881											
	**16.**	398	gi|475613321	Alpha-amylase inhibitor 0.19 [Aegilops tauschii]	17198											
	**17.**	389	gi|227809300	dimeric alpha-amylase inhibitor [Aegilops uniaristata]	15758											
	**18.**	389	gi|227809384	dimeric alpha-amylase inhibitor [Aegilops tauschii]	15791											
	**19.**	367	gi|54778515	0.19 dimeric alpha-amylase inhibitor [Aegilops tauschii]	13869											
	**20.**	364	gi|65993829	dimeric alpha-amylase inhibitor [Triticum aestivum]	15722											
	**21.**	364	gi|227809316	dimeric alpha-amylase inhibitor [Aegilops tauschii]	15704											
	**22.**	364	gi|227809370	dimeric alpha-amylase inhibitor [Aegilops tauschii]	15756											
	**23.**	355	gi|114215934	dimeric alpha-amylase inhibitor [Aegilops sharonensis]	13962											
	**24.**	355	gi|114215808	dimeric alpha-amylase inhibitor [Triticum dicoccoides]	13922											
	**25.**	355	gi|114215932	dimeric alpha-amylase inhibitor [Aegilops sharonensis]	13891											
	**26.**	355	gi|114215804	dimeric alpha-amylase inhibitor [Triticum dicoccoides]	13863											
	**27.**	355	gi|114215806	dimeric alpha-amylase inhibitor [Triticum dicoccoides]	13863											
	**28.**	354	gi|386877056	dimeric alpha-amylase inhibitor, partial [Aegilops longissima]	14792											
	**29.**	354	gi|386877058	dimeric alpha-amylase inhibitor, partial [Aegilops longissima]	14719											
	**30.**	353	gi|227809078	dimeric alpha-amylase inhibitor [Triticum dicoccoides]	15722											
	**31.**	353	gi|227809102	dimeric alpha-amylase inhibitor [Triticum dicoccoides]	15678											
	**32.**	353	gi|227809250	dimeric alpha-amylase inhibitor [Triticum dicoccoides]	15694											
	**33.**	353	gi|227809252	dimeric alpha-amylase inhibitor [Triticum dicoccoides]	15716											
	**34.**	353	gi|227809254	dimeric alpha-amylase inhibitor [Triticum dicoccoides]	15753											
	**35.**	353	gi|227809431	dimeric alpha-amylase inhibitor [Hordeum vulgare]	15605											
	**36.**	310	gi|54778503	0.19 dimeric alpha-amylase inhibitor [Triticum aestivum]	13827											
	**37.**	307	gi|65993925	dimeric alpha-amylase inhibitor [Triticum aestivum]	15702											
	**38.**	307	gi|227809156	dimeric alpha-amylase inhibitor [Triticum dicoccoides]	15716											
	**39.**	307	gi|227809366	dimeric alpha-amylase inhibitor [Aegilops tauschii]	15714											
	**40.**	306	gi|114216000	dimeric alpha-amylase inhibitor [Aegilops longissima]	13831											
	**41.**	299	gi|114215868	dimeric alpha-amylase inhibitor [Aegilops speltoides]	13929											
	**42.**	296	gi|227809415	dimeric alpha-amylase inhibitor [Henrardia persica]	15658											
	**43.**	288	gi|114215880	dimeric alpha-amylase inhibitor [Aegilops speltoides]	13964											
	**44.**	275	gi|227809348	dimeric alpha-amylase inhibitor [Secale cereale]	16037											
	**45.**	275	gi|227809350	dimeric alpha-amylase inhibitor [Secale cereale]	16058											
	**46.**	274	gi|386877054	dimeric alpha-amylase inhibitor, partial [Aegilops peregrina]	14146											
	**47.**	272	gi|227809344	dimeric alpha-amylase inhibitor [Secale cereale]	15945											
	**48.**	272	gi|227809119	dimeric alpha-amylase inhibitor [Triticum dicoccoides]	15678											
	**49.**	266	gi|386877042	dimeric alpha-amylase inhibitor, partial [Triticum aestivum]	11900											
	**50.**	264	gi|386877068	dimeric alpha-amylase inhibitor, partial [Triticum aestivum]	14416											
**F11**	**1.**	111	gi|66841026	**alpha-amylase inhibitor 0.19 [Triticum aestivum]**	13340	0.00025	8	1571.7830	1570.7757	1570.7773	-1.05	21	34	0	27	R.LQCNGSQVPEAVLR.D + Deamidated (NQ)
								1571.7830	1570.7757	1570.7773	-1.02	21	34	0	---	R.LQCNGSQVPEAVLR.D + Deamidated (NQ)
								1612.7384	1611.7311	1611.7390	-4.88	62	77	0	43	K.EHGAQEGQAGTGAFPR.C
								1612.7384	1611.7311	1611.7390	-4.88	62	77	0	---	K.EHGAQEGQAGTGAFPR.C
								1862.7573	1861.7500	1861.7658	-8.48	35	48	0	---	R.DCCQQLAHISEWCR.C
								1862.7573	1861.7500	1861.7658	-8.47	35	48	0	12	R.DCCQQLAHISEWCR.C
								2807.3130	2806.3057	2806.4357	-46.33	85	111	1	---	K.LTAASITAVCRLPIVVDASGDGAYVCK.D
								2807.3130	2806.3057	2806.4357	-46.33	85	111	1	---	K.LTAASITAVCRLPIVVDASGDGAYVCK.D
	**2.**	111	gi|123963	RecName: Full=Alpha-amylase inhibitor 0.19; AltName: Full=0.	13899											
	**3.**	110	gi|108597921	dimeric alpha-amylase inhibitor precursor, partial [Triticum aesti	14030											
	**4.**	110	gi|386877048	dimeric alpha-amylase inhibitor, partial [Aegilops kotschyi]	14199											
	**5.**	110	gi|386877062	dimeric alpha-amylase inhibitor, partial [Aegilops geniculata]	14543											
	**6.**	110	gi|386877046	dimeric alpha-amylase inhibitor, partial [Aegilops tauschii]	14670											
	**7.**	110	gi|386877044	dimeric alpha-amylase inhibitor, partial [Aegilops tauschii]	14805											
	**8.**	110	gi|386877050	dimeric alpha-amylase inhibitor, partial [Aegilops tauschii]	14826											
	**9.**	109	gi|386877060	dimeric alpha-amylase inhibitor, partial [Aegilops longissima]	14954											
	**10.**	109	gi|65993781	dimeric alpha-amylase inhibitor [Triticum aestivum]	15688											
	**11.**	109	gi|227809005	dimeric alpha-amylase inhibitor [Triticum dicoccoides]	15730											
	**12.**	109	gi|227809300	dimeric alpha-amylase inhibitor [Aegilops uniaristata]	15758											
	**13.**	109	gi|227809314	dimeric alpha-amylase inhibitor [Aegilops tauschii]	15762											
	**14.**	109	gi|227809364	dimeric alpha-amylase inhibitor [Aegilops tauschii]	15742											
	**15.**	109	gi|227809384	dimeric alpha-amylase inhibitor [Aegilops tauschii]	15791											

	**16.**	109	gi|386877038	dimeric alpha-amylase inhibitor [Triticum aestivum]	15702											
	**17.**	107	gi|475613321	Alpha-amylase inhibitor 0.19 [Aegilops tauschii]	17198											
	**18.**	107	gi|54778521	0.19 dimeric alpha-amylase inhibitor [Aegilops tauschii]	13881											
	**19.**	100	gi|386877068	dimeric alpha-amylase inhibitor, partial [Triticum aestivum]	14416											
	**20.**	100	gi|452055914	dimeric alpha-amylase inhibitor, partial [Aegilops geniculata]	14748											
	**21.**	99	gi|255988225	dimeric alpha-amylase inhibitor [Triticum aestivum]	15666											
	**22.**	97	gi|123956	RecName: Full=Alpha-amylase/trypsin inhibitor CM2; AltName:	16020											
	**23.**	89	gi|114215916	dimeric alpha-amylase inhibitor [Aegilops sharonensis]	13775											
	**24.**	89	gi|54778515	0.19 dimeric alpha-amylase inhibitor [Aegilops tauschii]	13869											
	**25.**	89	gi|114215934	dimeric alpha-amylase inhibitor [Aegilops sharonensis]	13962											
	**26.**	89	gi|114215808	dimeric alpha-amylase inhibitor [Triticum dicoccoides]	13922											
	**27.**	89	gi|114215932	dimeric alpha-amylase inhibitor [Aegilops sharonensis]	13891											
	**28.**	89	gi|114215804	dimeric alpha-amylase inhibitor [Triticum dicoccoides]	13863											
	**29.**	89	gi|114215806	dimeric alpha-amylase inhibitor [Triticum dicoccoides]	13863											
	**30.**	88	gi|386877056	dimeric alpha-amylase inhibitor, partial [Aegilops longissima]	14792											
	**31.**	88	gi|386877058	dimeric alpha-amylase inhibitor, partial [Aegilops longissima]	14719											
	**32.**	88	gi|65993829	dimeric alpha-amylase inhibitor [Triticum aestivum]	15722											
	**33.**	88	gi|227809078	dimeric alpha-amylase inhibitor [Triticum dicoccoides]	15722											
	**34.**	88	gi|227809102	dimeric alpha-amylase inhibitor [Triticum dicoccoides]	15678											
	**35.**	88	gi|227809250	dimeric alpha-amylase inhibitor [Triticum dicoccoides]	15694											
	**36.**	88	gi|227809252	dimeric alpha-amylase inhibitor [Triticum dicoccoides]	15716											
	**37.**	88	gi|227809254	dimeric alpha-amylase inhibitor [Triticum dicoccoides]	15753											
	**38.**	88	gi|227809316	dimeric alpha-amylase inhibitor [Aegilops tauschii]	15704											
	**39.**	88	gi|227809370	dimeric alpha-amylase inhibitor [Aegilops tauschii]	15756											
	**40.**	88	gi|227809431	dimeric alpha-amylase inhibitor [Hordeum vulgare]	15605											
**F12**	**1.**	633	gi|66841026	**alpha-amylase inhibitor 0.19 [Triticum aestivum]**	13340	1.6e-056	15	1162.6224	1161.6151	1161.6176	-2.12	85	95	0	---	K.LTAASITAVCR.L
								1162.6224	1161.6152	1161.6176	-2.08	85	95	0	66	K.LTAASITAVCR.L
								1538.6411	1537.6338	1537.6615	-17.99	49	61	0	---	R.CGALYSMLDSMYK.E
								1538.6411	1537.6338	1537.6615	-17.98	49	61	0	37	R.CGALYSMLDSMYK.E
								1554.6592	1553.6519	1553.6564	-2.88	49	61	0	---	R.CGALYSMLDSMYK.E + Oxidation (M)
								1570.7858	1569.7785	1569.7933	-9.43	21	34	0	---	R.LQCNGSQVPEAVLR.D
								1571.7729	1570.7656	1570.7773	-7.45	21	34	0	---	R.LQCNGSQVPEAVLR.D + Deamidated (NQ)
								1571.7730	1570.7657	1570.7773	-7.42	21	34	0	92	R.LQCNGSQVPEAVLR.D + Deamidated (NQ)
								1612.7275	1611.7202	1611.7390	-11.64	62	77	0	---	K.EHGAQEGQAGTGAFPR.C
								1612.7275	1611.7203	1611.7390	-11.61	62	77	0	141	K.EHGAQEGQAGTGAFPR.C
								1663.8167	1662.8094	1662.8287	-11.63	96	111	0	124	R.LPIVVDASGDGAYVCK.D
								1663.8167	1662.8094	1662.8287	-11.60	96	111	0	---	R.LPIVVDASGDGAYVCK.D
								1862.7498	1861.7425	1861.7658	-12.53	35	48	0	111	R.DCCQQLAHISEWCR.C
								1862.7498	1861.7425	1861.7658	-12.51	35	48	0	---	R.DCCQQLAHISEWCR.C
								2466.1035	2465.0962	2465.1785	-33.36	96	119	1	---	R.LPIVVDASGDGAYVCKDVAAYPDA.-
	**2.**	632	gi|123963	RecName: Full=Alpha-amylase inhibitor 0.19; AltName: Full=0.	13899											
	**3.**	632	gi|108597921	dimeric alpha-amylase inhibitor precursor, partial [Triticum aesti	14030											
	**4.**	631	gi|386877048	dimeric alpha-amylase inhibitor, partial [Aegilops kotschyi]	14199											
	**5.**	631	gi|386877062	dimeric alpha-amylase inhibitor, partial [Aegilops geniculata]	14543											
	**6.**	630	gi|386877046	dimeric alpha-amylase inhibitor, partial [Aegilops tauschii]	14670											
	**7.**	630	gi|386877044	dimeric alpha-amylase inhibitor, partial [Aegilops tauschii]	14805											
	**8.**	630	gi|386877050	dimeric alpha-amylase inhibitor, partial [Aegilops tauschii]	14826											
	**9.**	630	gi|386877060	dimeric alpha-amylase inhibitor, partial [Aegilops longissima]	14954											
	**10.**	628	gi|65993781	dimeric alpha-amylase inhibitor [Triticum aestivum]	15688											
	**11.**	628	gi|227809005	dimeric alpha-amylase inhibitor [Triticum dicoccoides]	15730											
	**12.**	628	gi|227809314	dimeric alpha-amylase inhibitor [Aegilops tauschii]	15762											
	**13.**	628	gi|227809364	dimeric alpha-amylase inhibitor [Aegilops tauschii]	15742											
	**14.**	628	gi|386877038	dimeric alpha-amylase inhibitor [Triticum aestivum]	15702											
	**15.**	628	gi|54778521	0.19 dimeric alpha-amylase inhibitor [Aegilops tauschii]	13881											
	**16.**	626	gi|475613321	Alpha-amylase inhibitor 0.19 [Aegilops tauschii]	17198											
	**17.**	581	gi|227809156	dimeric alpha-amylase inhibitor [Triticum dicoccoides]	15716											
	**18.**	579	gi|227809300	dimeric alpha-amylase inhibitor [Aegilops uniaristata]	15758											
	**19.**	579	gi|227809384	dimeric alpha-amylase inhibitor [Aegilops tauschii]	15791											

	**20.**	528	gi|54778503	0.19 dimeric alpha-amylase inhibitor [Triticum aestivum]	13827											
	**21.**	525	gi|65993925	dimeric alpha-amylase inhibitor [Triticum aestivum]	15702											
	**22.**	525	gi|227809366	dimeric alpha-amylase inhibitor [Aegilops tauschii]	15714											
	**23.**	508	gi|54778515	0.19 dimeric alpha-amylase inhibitor [Aegilops tauschii]	13869											
	**24.**	505	gi|65993829	dimeric alpha-amylase inhibitor [Triticum aestivum]	15722											
	**25.**	505	gi|227809316	dimeric alpha-amylase inhibitor [Aegilops tauschii]	15704											
	**26.**	505	gi|227809370	dimeric alpha-amylase inhibitor [Aegilops tauschii]	15756											
	**27.**	483	gi|386877068	dimeric alpha-amylase inhibitor, partial [Triticum aestivum]	14416											
	**28.**	483	gi|452055914	dimeric alpha-amylase inhibitor, partial [Aegilops geniculata]	14748											
	**29.**	476	gi|227809415	dimeric alpha-amylase inhibitor [Henrardia persica]	15658											
	**30.**	467	gi|114215868	dimeric alpha-amylase inhibitor [Aegilops speltoides]	13929											
	**31.**	459	gi|114215932	dimeric alpha-amylase inhibitor [Aegilops sharonensis]	13891											
	**32.**	459	gi|114215804	dimeric alpha-amylase inhibitor [Triticum dicoccoides]	13863											
	**33.**	459	gi|114215806	dimeric alpha-amylase inhibitor [Triticum dicoccoides]	13863											
	**34.**	458	gi|386877056	dimeric alpha-amylase inhibitor, partial [Aegilops longissima]	14792											
	**35.**	458	gi|386877058	dimeric alpha-amylase inhibitor, partial [Aegilops longissima]	14719											
	**36.**	457	gi|227809078	dimeric alpha-amylase inhibitor [Triticum dicoccoides]	15722											
	**37.**	457	gi|227809102	dimeric alpha-amylase inhibitor [Triticum dicoccoides]	15678											
	**38.**	457	gi|227809250	dimeric alpha-amylase inhibitor [Triticum dicoccoides]	15694											
	**39.**	457	gi|227809252	dimeric alpha-amylase inhibitor [Triticum dicoccoides]	15716											
	**40.**	457	gi|227809431	dimeric alpha-amylase inhibitor [Hordeum vulgare]	15605											
	**41.**	449	gi|114215934	dimeric alpha-amylase inhibitor [Aegilops sharonensis]	13962											
	**42.**	449	gi|114215808	dimeric alpha-amylase inhibitor [Triticum dicoccoides]	13922											
	**43.**	447	gi|227809254	dimeric alpha-amylase inhibitor [Triticum dicoccoides]	15753											
	**44.**	420	gi|114215880	dimeric alpha-amylase inhibitor [Aegilops speltoides]	13964											
	**45.**	416	gi|255988225	dimeric alpha-amylase inhibitor [Triticum aestivum]	15666											
	**46.**	383	gi|114216000	dimeric alpha-amylase inhibitor [Aegilops longissima]	13831											
	**47.**	370	gi|227809119	dimeric alpha-amylase inhibitor [Triticum dicoccoides]	15678											
	**48.**	361	gi|386877040	dimeric alpha-amylase inhibitor, partial [Triticum aestivum]	15014											
	**49.**	357	gi|114215840	dimeric alpha-amylase inhibitor [Aegilops speltoides]	13933											
	**50.**	357	gi|386877054	dimeric alpha-amylase inhibitor, partial [Aegilops peregrina]	14146											
**F13**	**1.**	399	gi|66841026	**alpha-amylase inhibitor 0.19 [Triticum aestivum]**	13340	3.9e-033	13	1162.6127	1161.6054	1161.6176	-10.49	85	95	0	41	K.LTAASITAVCR.L
								1162.6127	1161.6054	1161.6176	-10.47	85	95	0	---	K.LTAASITAVCR.L
								1570.7692	1569.7619	1569.7933	-20.00	21	34	0	---	R.LQCNGSQVPEAVLR.D
								1571.7609	1570.7536	1570.7773	-15.12	21	34	0	66	R.LQCNGSQVPEAVLR.D + Deamidated (NQ)
								1571.7609	1570.7536	1570.7773	-15.09	21	34	0	---	R.LQCNGSQVPEAVLR.D + Deamidated (NQ)
								1612.7191	1611.7118	1611.7390	-16.85	62	77	0	---	K.EHGAQEGQAGTGAFPR.C
								1612.7191	1611.7118	1611.7390	-16.84	62	77	0	87	K.EHGAQEGQAGTGAFPR.C
								1663.8049	1662.7976	1662.8287	-18.70	96	111	0	---	R.LPIVVDASGDGAYVCK.D
								1663.8049	1662.7977	1662.8287	-18.68	96	111	0	128	R.LPIVVDASGDGAYVCK.D
								1862.7421	1861.7348	1861.7658	-16.66	35	48	0	24	R.DCCQQLAHISEWCR.C
								1862.7421	1861.7348	1861.7658	-16.64	35	48	0	---	R.DCCQQLAHISEWCR.C
								2807.2820	2806.2747	2806.4357	-57.38	85	111	1	---	K.LTAASITAVCRLPIVVDASGDGAYVCK.D
								2807.2820	2806.2747	2806.4357	-57.38	85	111	1	---	K.LTAASITAVCRLPIVVDASGDGAYVCK.D
	**2.**	398	gi|123963	RecName: Full=Alpha-amylase inhibitor 0.19; AltName: Full=0.	13899											
	**3.**	398	gi|108597921	dimeric alpha-amylase inhibitor precursor, partial [Triticum aesti	14030											
	**4.**	397	gi|386877048	dimeric alpha-amylase inhibitor, partial [Aegilops kotschyi]	14199											
	**5.**	397	gi|386877062	dimeric alpha-amylase inhibitor, partial [Aegilops geniculata]	14543											
	**6.**	396	gi|386877046	dimeric alpha-amylase inhibitor, partial [Aegilops tauschii]	14670											
	**7.**	396	gi|386877044	dimeric alpha-amylase inhibitor, partial [Aegilops tauschii]	14805											
	**8.**	396	gi|386877050	dimeric alpha-amylase inhibitor, partial [Aegilops tauschii]	14826											
	**9.**	396	gi|386877060	dimeric alpha-amylase inhibitor, partial [Aegilops longissima]	14954											
	**10.**	395	gi|65993781	dimeric alpha-amylase inhibitor [Triticum aestivum]	15688											
	**11.**	395	gi|227809005	dimeric alpha-amylase inhibitor [Triticum dicoccoides]	15730											
	**12.**	395	gi|227809300	dimeric alpha-amylase inhibitor [Aegilops uniaristata]	15758											
	**13.**	395	gi|227809314	dimeric alpha-amylase inhibitor [Aegilops tauschii]	15762											
	**14.**	395	gi|227809364	dimeric alpha-amylase inhibitor [Aegilops tauschii]	15742											
	**15.**	395	gi|227809384	dimeric alpha-amylase inhibitor [Aegilops tauschii]	15791											

	**16.**	395	gi|386877038	dimeric alpha-amylase inhibitor [Triticum aestivum]	15702											
	**17.**	394	gi|54778521	0.19 dimeric alpha-amylase inhibitor [Aegilops tauschii]	13881											
	**18.**	393	gi|475613321	Alpha-amylase inhibitor 0.19 [Aegilops tauschii]	17198											
	**19.**	375	gi|227809156	dimeric alpha-amylase inhibitor [Triticum dicoccoides]	15716											
	**20.**	362	gi|54778515	0.19 dimeric alpha-amylase inhibitor [Aegilops tauschii]	13869											
	**21.**	362	gi|114215934	dimeric alpha-amylase inhibitor [Aegilops sharonensis]	13962											
	**22.**	362	gi|114215808	dimeric alpha-amylase inhibitor [Triticum dicoccoides]	13922											
	**23.**	362	gi|114215932	dimeric alpha-amylase inhibitor [Aegilops sharonensis]	13891											
	**24.**	362	gi|114215804	dimeric alpha-amylase inhibitor [Triticum dicoccoides]	13863											
	**25.**	362	gi|114215806	dimeric alpha-amylase inhibitor [Triticum dicoccoides]	13863											
	**26.**	361	gi|386877056	dimeric alpha-amylase inhibitor, partial [Aegilops longissima]	14792											
	**27.**	361	gi|386877058	dimeric alpha-amylase inhibitor, partial [Aegilops longissima]	14719											
	**28.**	359	gi|65993829	dimeric alpha-amylase inhibitor [Triticum aestivum]	15722											
	**29.**	359	gi|227809078	dimeric alpha-amylase inhibitor [Triticum dicoccoides]	15722											
	**30.**	359	gi|227809102	dimeric alpha-amylase inhibitor [Triticum dicoccoides]	15678											
	**31.**	359	gi|227809250	dimeric alpha-amylase inhibitor [Triticum dicoccoides]	15694											
	**32.**	359	gi|227809252	dimeric alpha-amylase inhibitor [Triticum dicoccoides]	15716											
	**33.**	359	gi|227809254	dimeric alpha-amylase inhibitor [Triticum dicoccoides]	15753											
	**34.**	359	gi|227809316	dimeric alpha-amylase inhibitor [Aegilops tauschii]	15704											
	**35.**	359	gi|227809370	dimeric alpha-amylase inhibitor [Aegilops tauschii]	15756											
	**36.**	359	gi|227809431	dimeric alpha-amylase inhibitor [Hordeum vulgare]	15605											
	**37.**	330	gi|54778503	0.19 dimeric alpha-amylase inhibitor [Triticum aestivum]	13827											
	**38.**	327	gi|65993925	dimeric alpha-amylase inhibitor [Triticum aestivum]	15702											
	**39.**	327	gi|227809366	dimeric alpha-amylase inhibitor [Aegilops tauschii]	15714											
	**40.**	300	gi|114216000	dimeric alpha-amylase inhibitor [Aegilops longissima]	13831											
	**41.**	299	gi|227809348	dimeric alpha-amylase inhibitor [Secale cereale]	16037											
	**42.**	299	gi|227809350	dimeric alpha-amylase inhibitor [Secale cereale]	16058											
	**43.**	299	gi|114215880	dimeric alpha-amylase inhibitor [Aegilops speltoides]	13964											
	**44.**	299	gi|114215868	dimeric alpha-amylase inhibitor [Aegilops speltoides]	13929											
	**45.**	296	gi|227809415	dimeric alpha-amylase inhibitor [Henrardia persica]	15658											
	**46.**	296	gi|386877054	dimeric alpha-amylase inhibitor, partial [Aegilops peregrina]	14146											
	**47.**	284	gi|114215840	dimeric alpha-amylase inhibitor [Aegilops speltoides]	13933											
	**48.**	283	gi|227809403	dimeric alpha-amylase inhibitor [Aegilops comosa]	15714											
	**49.**	283	gi|227809409	dimeric alpha-amylase inhibitor [Aegilops comosa]	15758											
	**50.**	275	gi|114215958	dimeric alpha-amylase inhibitor [Aegilops searsii]	13926											
**F14**	**1.**	179	gi|34925030	**RecName: Full=Wheatwin-1; AltName: Full=Pathogenesis-rel**	16024	3.9e-011	11	1700.8341	1699.8268	1699.8676	-23.96	87	102	0	---	K.CLQVTNPATGAQITAR.I
								1700.8341	1699.8268	1699.8676	-23.95	87	102	0	50	K.CLQVTNPATGAQITAR.I
								2082.8484	2081.8411	2081.8836	-20.42	67	86	0	26	K.YGWTAFCGPAGAHGQASCGK.C
								2082.8484	2081.8411	2081.8836	-20.42	67	86	0	---	K.YGWTAFCGPAGAHGQASCGK.C
								2267.0220	2266.0147	2266.0576	-18.95	103	122	0	54	R.IVDQCANGGLDLDWDTVFTK.I
								2267.0220	2266.0147	2266.0576	-18.94	103	122	0	---	R.IVDQCANGGLDLDWDTVFTK.I
								2268.0090	2267.0017	2267.0416	-17.61	103	122	0	---	R.IVDQCANGGLDLDWDTVFTK.I + Deamidated (NQ)
								2712.1965	2711.1892	2711.2398	-18.66	123	145	0	---	K.IDTNGIGYQQGHLNVNYQFVDCR.D + Deamidated (NQ)
								2826.2251	2825.2178	2825.2828	-22.98	123	146	1	13	K.IDTNGIGYQQGHLNVNYQFVDCRD.-
								2826.2251	2825.2178	2825.2828	-22.98	123	146	1	---	K.IDTNGIGYQQGHLNVNYQFVDCRD.-
								2827.2251	2826.2178	2826.2668	-17.32	123	146	1	---	K.IDTNGIGYQQGHLNVNYQFVDCRD.- + Deamidated (NQ)
	**2.**	158	gi|6048567	PR-4 [Triticum aestivum]	13430											
	**3.**	146	gi|453118	wheatwin1=barwin homolog [Triticum aestivum=wheat, San Pas	13929											
	**4.**	127	gi|326524784	predicted protein [Hordeum vulgare subsp. vulgare]	16142											
	**5.**	119	gi|114832	RecName: Full=Barwin	14071											
	**6.**	119	gi|159162134	Chain A, Three-Dimensional Structure In Solution Of Barwin, A	14072											
	**7.**	117	gi|475510915	Wheatwin-2 [Aegilops tauschii]	16024											
	**8.**	116	gi|34925032	RecName: Full=Wheatwin-2; AltName: Full=Pathogenesis-relate	16256											
	**9.**	96	gi|6048569	PR-4 [Triticum aestivum]	13442											
**F15**	**1.**	177	gi|134034615	**monomeric alpha-amylase inhibitor [Triticum aestivum]**	13617	6.3e-011	11	830.4114	829.4041	829.4116	-9.04	77	83	0	---	K.EVLPGCR.K
								830.4114	829.4042	829.4116	-8.99	77	83	0	8	K.EVLPGCR.K

1050.5478	1049.5406	1049.5506	-9.51	65	73	0	15	R.SVYQELGVR.E
1050.5479	1049.5406	1049.5506	-9.46	65	73	0	---	R.SVYQELGVR.E
1051.5446	1050.5373	1050.5346	2.63	65	73	0	---	R.SVYQELGVR.E + Deamidated (NQ)
1555.7920	1554.7847	1554.8188	-21.93	27	40	0	34	K.LQCVGSQVPEAVLR.D
1555.7920	1554.7847	1554.8188	-21.93	27	40	0	---	K.LQCVGSQVPEAVLR.D
1611.6543	1610.6470	1610.6824	-21.96	1	14	0	49	-.SGPWSWCDPATGYK.V
1611.6543	1610.6470	1610.6824	-21.96	1	14	0	---	-.SGPWSWCDPATGYK.V
1981.7433	1980.7360	1980.7877	-26.07	41	55	0	25	R.DCCQQLADINNEWCR.C
1981.7433	1980.7360	1980.7877	-26.07	41	55	0	---	R.DCCQQLADINNEWCR.C

**2.**	177	gi|134034521	monomeric alpha-amylase inhibitor [Triticum monococcum]	13717
**3.**	177	gi|134034506	monomeric alpha-amylase inhibitor [Triticum monococcum]	13659
**4.**	177	gi|134034613	monomeric alpha-amylase inhibitor [Triticum aestivum]	13638
**5.**	177	gi|134034508	monomeric alpha-amylase inhibitor [Triticum monococcum]	13658
**6.**	177	gi|134034577	monomeric alpha-amylase inhibitor [Triticum aestivum]	13668
**7.**	177	gi|134034537	monomeric alpha-amylase inhibitor [Triticum monococcum]	13699
**8.**	160	gi|134034565	monomeric alpha-amylase inhibitor [Aegilops tauschii]	13749
**9.**	160	gi|223520	inhibitor,alpha amylase	13961
**10.**	156	gi|134034545	monomeric alpha-amylase inhibitor [Aegilops bicornis]	13704
**11.**	139	gi|134034641	monomeric alpha-amylase inhibitor [Triticum aestivum]	13587
**12.**	139	gi|134034539	monomeric alpha-amylase inhibitor [Triticum monococcum]	13643
**13.**	123	gi|283465829	putative alpha-amylase inhibitor 0.28, partial [Triticum aestivum	13743
**14.**	113	gi|227809342	dimeric alpha-amylase inhibitor [Heteranthelium piliferum]	15595
**15.**	112	gi|2894148	monomeric alpha-amylase inhibitor [Triticum aestivum]	17187
**16.**	112	gi|229614961	monomeric alpha-amylase inhibitor [Triticum dicoccoides]	17104
**17.**	112	gi|229614963	monomeric alpha-amylase inhibitor [Triticum dicoccoides]	17074
**18.**	112	gi|229614967	monomeric alpha-amylase inhibitor [Triticum dicoccoides]	17074
**19.**	112	gi|229614977	monomeric alpha-amylase inhibitor [Triticum dicoccoides]	17173
**20.**	112	gi|229614983	monomeric alpha-amylase inhibitor [Triticum dicoccoides]	17104
**21.**	112	gi|229614993	monomeric alpha-amylase inhibitor [Triticum dicoccoides]	17102
**22.**	112	gi|229615039	monomeric alpha-amylase inhibitor [Triticum dicoccoides]	17118
**23.**	112	gi|229615043	monomeric alpha-amylase inhibitor [Triticum dicoccoides]	17044
**24.**	112	gi|229615063	monomeric alpha-amylase inhibitor [Triticum dicoccoides]	17203
**25.**	112	gi|229615085	monomeric alpha-amylase inhibitor [Triticum dicoccoides]	17106
**26.**	112	gi|229615087	monomeric alpha-amylase inhibitor [Triticum dicoccoides]	17044
**27.**	112	gi|229615107	monomeric alpha-amylase inhibitor [Triticum dicoccoides]	17074
**28.**	112	gi|229615111	monomeric alpha-amylase inhibitor [Triticum dicoccoides]	17076
**29.**	112	gi|229615115	monomeric alpha-amylase inhibitor [Triticum dicoccoides]	17094
**30.**	112	gi|229615123	monomeric alpha-amylase inhibitor [Triticum dicoccoides]	17044
**31.**	112	gi|229615147	monomeric alpha-amylase inhibitor [Triticum dicoccoides]	17030
**32.**	112	gi|229615151	monomeric alpha-amylase inhibitor [Triticum dicoccoides]	17176
**33.**	112	gi|229615171	monomeric alpha-amylase inhibitor [Triticum dicoccoides]	17056
**34.**	112	gi|229615179	monomeric alpha-amylase inhibitor [Triticum dicoccoides]	17130
**35.**	112	gi|229615183	monomeric alpha-amylase inhibitor [Triticum dicoccoides]	17130
**36.**	112	gi|229615199	monomeric alpha-amylase inhibitor [Triticum dicoccoides]	17231
**37.**	112	gi|229615203	monomeric alpha-amylase inhibitor [Triticum dicoccoides]	17143
**38.**	112	gi|229615233	monomeric alpha-amylase inhibitor [Triticum dicoccoides]	17072
**39.**	112	gi|229615253	monomeric alpha-amylase inhibitor [Triticum dicoccoides]	17003
**40.**	112	gi|229615259	monomeric alpha-amylase inhibitor [Triticum dicoccoides]	17132
**41.**	112	gi|229615322	monomeric alpha-amylase inhibitor [Triticum dicoccoides]	17054
**42.**	112	gi|229615328	monomeric alpha-amylase inhibitor [Triticum dicoccoides]	17086
**43.**	112	gi|229615336	monomeric alpha-amylase inhibitor [Triticum dicoccoides]	17108
**44.**	112	gi|229615380	monomeric alpha-amylase inhibitor [Triticum dicoccoides]	17103
**45.**	112	gi|229615398	monomeric alpha-amylase inhibitor [Triticum dicoccoides]	17070
**46.**	112	gi|229615470	monomeric alpha-amylase inhibitor [Triticum dicoccoides]	17072
**47.**	112	gi|229615474	monomeric alpha-amylase inhibitor [Triticum dicoccoides]	17206
**48.**	112	gi|229615476	monomeric alpha-amylase inhibitor [Triticum dicoccoides]	17177
**49.**	112	gi|229615540	monomeric alpha-amylase inhibitor [Triticum dicoccoides]	17233
**50.**	112	gi|229615542	monomeric alpha-amylase inhibitor [Triticum dicoccoides]	17044

**F16**	**1.**	89	gi|494377469	**regulator [Bacillus macauensis]**	25566	0.036	14	762.4091	761.4018	761.4469	-59.29	202	207	1	---	K.LCKLTK.A
								762.4091	761.4018	761.4469	-59.24	202	207	1	---	K.LCKLTK.A
								856.5206	855.5133	855.4814	37.3	148	154	0	---	R.EIQLTPR.E
								856.5206	855.5134	855.4814	37.4	148	154	0	---	R.EIQLTPR.E
								870.5372	869.5299	869.5698	-45.87	74	81	0	---	K.AILILTAR.D
								913.5411	912.5338	912.5029	33.9	165	172	1	---	R.NKGQVLPR.E + 2 Deamidated (NQ)
								913.5411	912.5339	912.5029	34.0	165	172	1	11	R.NKGQVLPR.E + 2 Deamidated (NQ)
								1851.8000	1850.7927	1850.8947	-55.08	42	57	0	---	R.ASEFDVLILDWMMPGK.T
								2239.0825	2238.0752	2238.0090	29.6	23	41	0	---	K.NQYHVEWVHNGEDALEAAR.A + Deamidated (NQ)
								2239.0825	2238.0752	2238.0090	29.6	23	41	0	4	K.NQYHVEWVHNGEDALEAAR.A + Deamidated (NQ)
								2278.0918	2277.0845	2277.2515	-73.30	2	21	1	---	M.NILAAEDDLQLGRLIVHMLK.K + Oxidation (M)
								2278.0918	2277.0845	2277.2515	-73.30	2	21	1	---	M.NILAAEDDLQLGRLIVHMLK.K + Oxidation (M)
								2411.1548	2410.1475	2410.2599	-46.65	1	21	1	---	-.MNILAAEDDLQLGRLIVHMLK.K + 2 Deamidated (NQ); Oxidation (M)
								2411.1548	2410.1475	2410.2599	-46.64	1	21	1	---	-.MNILAAEDDLQLGRLIVHMLK.K + 2 Deamidated (NQ); Oxidation (M)
**F17**	**1.**	91	gi|516638230	**argininosuccinate synthase [Sinorhizobium medicae]**	44937	0.024	18	832.5038	831.4965	831.4273	83.3	353	360	0	---	K.GNVMVVGR.E + Deamidated (NQ)
								837.4882	836.4809	836.4868	-7.09	105	111	0	5	K.HLIDIAR.K
								837.4882	836.4809	836.4868	-7.04	105	111	0	---	K.HLIDIAR.K
								856.5424	855.5351	855.5290	7.17	392	398	1	---	K.LNALRLR.T + Deamidated (NQ)
								856.5424	855.5351	855.5290	7.19	392	398	1	19	K.LNALRLR.T + Deamidated (NQ)
								965.5118	964.5045	964.5818	-80.08	105	112	1	---	K.HLIDIARK.T
								965.5118	964.5046	964.5818	-80.04	105	112	1	---	K.HLIDIARK.T
								1036.5461	1035.5388	1035.5237	14.7	62	69	0	---	K.EIYIEDVR.E
								1036.5461	1035.5389	1035.5237	14.7	62	69	0	---	K.EIYIEDVR.E
								1065.5317	1064.5244	1064.5390	-13.67	158	166	0	---	R.TDLLEFAEK.H
								1065.5317	1064.5245	1064.5390	-13.63	158	166	0	---	R.TDLLEFAEK.H
								1074.5417	1073.5344	1073.5005	31.6	75	82	0	---	K.DFVFPMFR.A + Oxidation (M)
								1074.5418	1073.5345	1073.5005	31.7	75	82	0	---	K.DFVFPMFR.A + Oxidation (M)
								1232.6145	1231.6072	1231.6343	-21.99	353	364	1	---	K.GNVMVVGRESGK.S
								1308.6754	1307.6681	1307.6721	-3.04	156	166	1	---	K.SRTDLLEFAEK.H
								1383.7021	1382.6948	1382.6830	8.54	379	391	1	---	R.GAYDQKDAAGFIK.L
								1638.8743	1637.8670	1637.8446	13.7	242	256	1	---	R.MSAATLLAKLNEYGR.D + Deamidated (NQ)
								1657.8098	1656.8025	1656.7631	23.8	371	384	1	---	K.LVTFEDDRGAYDQK.D + Deamidated (NQ)
	**2.**	87	gi|383856370	PREDICTED: spectrin beta chain, brain 4-like [Megachile rotun	495973											
	**3.**	87	gi|339444837	hypothetical protein EGYY_12790 [Eggerthella sp. YY7918]	52311											
